# Clathrin adaptors drive phase separation in endocytosis and trafficking

**DOI:** 10.1038/s42003-026-11043-9

**Published:** 2026-09-26

**Authors:** George Draper-Barr, Janina Schiller, Katharina Veith, Stephan Niebling, David Ruiz-Carrillo, Ziqiang Huang, Christian Tischer, Roland Thuenauer, Lucas A. Defelipe, Maria García-Alai

**Affiliations:** 1 European Molecular Biology Laboratory, Hamburg, Germany; 2 Centre for Structural Systems Biology (CSSB), Deutsches Elektronen-Synchrotron DESY, Hamburg, Germany; 3 EMBL Imaging Centre, European Molecular Biology Laboratory, Heidelberg, Germany; 4 European Molecular Biology Laboratory, Data Science Centre, Heidelberg, Germany; 5 Technology Platform Light Microscopy (TPLM), Universität Hamburg (UHH), Hamburg, Germany

**Keywords:** Endocytosis, Structural biology

## Abstract

Liquid–liquid phase separation (LLPS) underlies the formation of biomolecular condensates that organize processes, including endocytosis and membrane trafficking. Although LLPS has been implicated in clathrin-mediated pathways, the contribution of adaptor proteins to condensate formation and function remains unclear. Here we show that two yeast adaptors, Ent5 and Sla2, undergo LLPS in vitro and this property correlates with their roles in membrane trafficking and condensate recruitment in vivo. Ent5 condensation is driven by a dynamic helix within its disordered region that acts as a molecular “sticker”. Deletion of this 249-287 helix disrupts membrane-associated condensation and leads to altered coating dynamics in vivo, with Ent5-dependent transport events becoming delayed. In contrast, Sla2 behaves as a driver and as a client of LLPS, with its coiled-coil (CC) region mediating condensation. Together, these findings reveal that endocytic adaptors can promote condensate formation through distinct structural features, coupling clathrin binding and membrane association through phase separation.

## Introduction

Biological condensates form in cells when macromolecules, primarily proteins or nucleic acids, undergo liquid-liquid phase separation (LLPS), transitioning from an initially solubilized form into at least two distinct phases. This process is driven by a shift in interaction preferences: from macromolecule-water interactions to water-water and macromolecule-macromolecule interactions. In vitro, the simplest case of LLPS involves the formation of a dense phase in the form of round droplets, surrounded by a dilute aqueous environment^1^. These dense droplets retain liquid-like properties, allowing for molecular exchange with the dilute phase, fusion with other droplets, and growth^2^. Although LLPS is described as a spontaneous phenomenon, it is tightly regulated by the unique characteristics of each macromolecule, as well as by solvent composition, macromolecular concentration, temperature, and other environmental factors^1,3,4^.

When LLPS-formed, protein-rich condensates come into close contact with the surface of the cell or organelle membrane, they can adhere to it, “wetting” the membrane and leading to its deformation. This interaction plays a key role in biologically relevant processes such as budding, nanotube formation, compartmentalization, and membrane fission^5^. Moreover, LLPS driven by intrinsically disordered proteins (IDPs) can exert steric pressure, due to their large hydrodynamic radii, sufficient to induce membrane bending^6^.

IDPs play diverse functional roles in biomolecular condensates. They can drive phase separation, tune the physical and biochemical properties of condensates, suppress aberrant aggregation, and act as regulatory elements that modulate condensate behavior in response to post-translational modifications^6^. IDPs contain “*stickers and spacers”: stickers* are responsible for percolation and form reversible interactions through specific residues or motifs in intrinsically disordered regions (IDRs). These may include inducible secondary structural elements, such as helices or beta strands, which can further influence percolation and network stability^7^. *Spacers*, in contrast, modulate solubility and influence sticker-sticker cooperativity. IDRs contribute uniquely to LLPS by enabling multivalent molecular interactions with a high degree of regulatory flexibility^8,9^. In addition, Ramirez et al.^10^ have shown that coiled-coil (CC) structures possess physical features that enable them to drive the LLPS of proteins. CC-containing proteins can potentially utilize both polymeric and multimeric multivalency mechanisms to promote LLPS through their CC domains. Among these, multimerization has a strong effect on LLPS propensity, while polymer multivalency has a weaker influence.

When LLPS is coupled with percolation, specific interactions facilitate the formation of small percolating clusters composed of one or more types of macromolecules^9,11^. Macromolecules essential for LLPS are termed *drivers*, as they can undergo phase separation independently. In contrast, *clients* are molecules that do not phase-separate on their own but can partition into condensates formed by drivers^12,13^.

Clathrin-mediated endocytosis requires the concerted action of membrane-binding proteins, known as adaptors, which link the membrane, the clathrin scaffold, and the actin polymerization machinery. This coordinated interaction results in membrane invagination and vesicle formation. Ede1, the yeast homolog of mammalian Eps15, is an early-arriving endocytic protein and a key initiation factor, although it does not bind directly to clathrin. In the absence of Ede1, most other early endocytic proteins lose their punctate localization, and endocytic uptake is significantly reduced. When present above a threshold concentration, Ede1 forms condensates that recruit other endocytic proteins and exhibit properties characteristic of phase-separated liquid droplets. The central region of Ede1, which contains a coiled-coil domain, is essential for both condensate formation and Ede1’s function in endocytosis^14^.

In order to understand whether the clathrin scaffold could function as a lattice embedded within a phase-separating environment, and which adaptors might nucleate or regulate this process, we conducted a screening experiment to investigate the role of LLPS in clathrin-mediated endocytosis and trafficking proteins. We selected several clathrin-binding adaptors based on their N-terminal domain interactions with clathrin heavy chain (NTD-CHC), as defined by Defelipe et al.^15^, and with clathrin light chain (CLC)^16^.

In this study, we report that Ent5, an epsin involved in trafficking, and Sla2, a key component of the endocytic mid-coat, both undergo LLPS. We further characterize their roles as drivers and clients. Ent5 consists of an Epsin N-Terminal Homology (ENTH) domain, which has been associated with phosphatidylinositol-3,5-bisphosphate (PI(3,5)P₂)-dependent membrane recruitment^17–20^, followed by an IDR^21^. We demonstrate that Ent5 promotes phase separation through a *sticker*-inducible α-helix and is capable of recruiting clathrin and Ent3. In contrast, Sla2, the yeast homolog of mammalian Hip1r, can be recruited by Ede1 condensates^14^ as a client but also induces LLPS and thus behaves as a driver. Truncation and complementation analyzes indicate that the central coiled-coil-containing and Regulatory and Mechanical Domain (REND) regions contribute to this activity, particularly in a membrane environment.

## Results

### Ent5 and Sla2 form liquid condensates in vitro

The clathrin triskelion is a three-legged structure composed of three clathrin heavy chains (CHC) and three CLCs. It self-assembles into a polyhedral lattice, forming a scaffold on the inner surface of the cell membrane: the clathrin coat^22^. The N-terminal domain of CHC (NTD-CHC) is responsible for interacting with proteins that contain a Clathrin Binding Motif (CBM)^15^. CHC lacks significant IDRs and, accordingly, is not predicted to form condensates spontaneously. In contrast, CLC contains an unstructured region known to interact with Sla2^16^ and a long ɑ-helix that binds to the distal domain of CHC. Unlike CHC, CLC is predicted to spontaneously undergo phase separation (see Table 1).

**Table 1:** Proteins involved in endocytosis and trafficking

Name	UniProt ID	IDR	Membrane binding	Function	LLPS prediction^a^
Apl2	P36000	Yes	Yes	AP1 Complex - TGN Trafficking	No
Apl5	Q08951	Yes	Yes	AP3 Complex - Golgi	No
CHC	P22137	No	No	Formation of Protein Coat	No
CLC	P17891	Yes	No	Regulation of Coat Assembly	Yes
Ede1	P34216	Yes	No	Endocytosis initiation factor	Yes
Ent1	Q12518	Yes	Yes	Adaptor protein in Endocytosis	Yes
Ent2	Q05785	Yes	Yes	Adaptor protein in Endocytosis	Yes
Ent3	P47160	Yes	Yes	Adaptor protein in TGN Trafficking	Yes
Ent5	Q03769	Yes	Yes	Adaptor protein in TGN and endosomal Trafficking	Yes
Gga1	Q06336	Yes	Yes	TGN Trafficking	No
Gga2	P38817	Yes	Yes	TGN Trafficking	Yes
Sla1	P32790	Yes	No	Cytoskeletal protein binding protein; required for assembly of the cortical actin cytoskeleton	Yes
Sla2	P33338	Yes	Yes	Core component of the endocytic machinery at the plasma membrane. Connects the membrane with the actin cytoskeleton	No
Swa2	Q06677	Yes	No	Cofactor for the uncoating of clathrin-coated vesicles (CCVs) by Hsp70-type chaperones	Yes
Yap1801	P38856	Yes	Yes	Clathrin Adaptor Protein	Yes
Yap1802	P53309	Yes	Yes	Clathrin Adaptor Protein	Yes

^a^Prediction from FuzDrop. Probability of Spontaneous LLPS > 0.6 is considered predictive of condensate formation.

To test the hypothesis that clathrin adaptors are prone to induce LLPS, we performed a combined analysis using AI-based modeling for IDR prediction and in vitro phase separation experiments of adaptors known to bind clathrin and participate in endocytosis or trans-Golgi network (TGN) trafficking. Our screening identified several proteins with the potential to undergo phase separation, based on predictions from FuzDrop^23,24^, with a probability of spontaneous LLPS greater than 0.6 (see Table 1, and Supplementary Table S1). Supplementary Table S1 includes information about the IDRs, specifically highlighting the “Droplet-promoting regions”, those predicted residues to be involved in intramolecular interactions such as percolation. These regions could potentially act as LLPS “*stickers*”.

Ede1, known to form condensates spontaneously due to its extensive IDRs^14^, was detected as a positive hit in our screening. Ent1 and Ent2 are epsin-family adaptors that contribute to endocytic coat assembly through N-terminal ENTH domains and C-terminal IDRs containing CBMs^15^. Yap1801 and Yap1802 are AP180/CALM-like clathrin adaptors involved in cargo sorting and clathrin recruitment during endocytosis^24^. All four proteins are predicted to spontaneously form condensates (Table 1).

Sla2 contains an N-terminal AP180 N-terminal homology (ANTH) domain followed by an IDR, a central coiled-coil-containing region that includes the REND domain, a talin/Hip1R/Sla2p actin-tethering C-terminal homology (THATCH) domain, and a C-terminal regulatory helix (Supplementary Fig. 1). Despite containing an IDR, Sla2 is not predicted to spontaneously phase separate according to our computational analysis. Nevertheless, Sla2 participates in a highly interconnected interaction network within the endocytic coat. Previous work showed that the coiled-coil-containing region of Sla2 interacts with the endocytic scaffold Pan1 with an affinity comparable to that of its interaction with CLC, while the Sla2 IDR mediates interactions with Sla1^16^. These observations suggest that Sla2 contains multiple regions capable of contributing to interaction valency despite its modest LLPS prediction score. In contrast, Sla1, which cooperates with Sla2 in regulating actin polymerization, contains three Src homology 3 (SH3) domains, a membrane-interacting pleckstrin homology (PH) domain, and an IDR^16^ and is predicted to spontaneously phase separate (Table 1).

Among other targets predicted to undergo spontaneous phase separation, we identified Swa2, an auxilin-like protein involved in clathrin uncoating. Swa2 includes three tetratricopeptide repeat (TPR) domains (which interact with Hsp70-type chaperones), a J domain that stimulates the ATPase activity of the chaperone, and several IDRs.

Regarding proteins involved in intracellular trafficking, Ent3 and Ent5 both contain N-terminal ENTH domains and C-terminal IDRs, similar to Ent1 and Ent2. However, Ent3 and Ent5 function primarily in the trans-Golgi network and endosomal trafficking pathways rather than at the plasma membrane. Consistent with their subcellular localization, their recruitment has been linked to PI(3,5)P₂-containing membranes. Both Ent3 and Ent5 are predicted to spontaneously phase separate.

Gga1 and Gga2, trans-Golgi adaptor proteins, recognize linear motifs (DxxLL) through their Vps27, Hrs, and STAM (VPS) domains and contain Gamma-adaptin Ear (GAE) domains that mediate protein-protein interactions^25,26^. Although both possess long IDRs, Gga2 is predicted to spontaneously phase separate, while Gga1 is not. This difference can be partly explained by droplet-promoting predictions from FuzDrop: whereas Gga2 has long droplet-promoting regions (Supplementary Table S1), Gga1 lacks them.

Lastly, Apl2 (AP-1 subunit beta) and Apl5 (AP-3 subunit delta) both contain multiple Armadillo-like repeats and relatively short IDRs. Neither Apl2 nor Apl5 is predicted to spontaneously phase separate, likely due to the insufficient length and biophysical properties of their IDRs.

From the candidates identified in Table 1 (see also Supplementary Table S1), we selected four membrane-associated adaptors involved in endocytosis or membrane trafficking for experimental validation.

Among the epsins, we focused on Ent1, Ent3, and Ent5, all of which contain ENTH membrane-binding domains. Because Ent1 and Ent2 are closely related paralogues with overlapping functions, we selected Ent1 as a representative of this family. Ent3 and Ent5 were included because they function in intracellular trafficking pathways and represent a distinct branch of the yeast epsins.

**Fig. 1:**
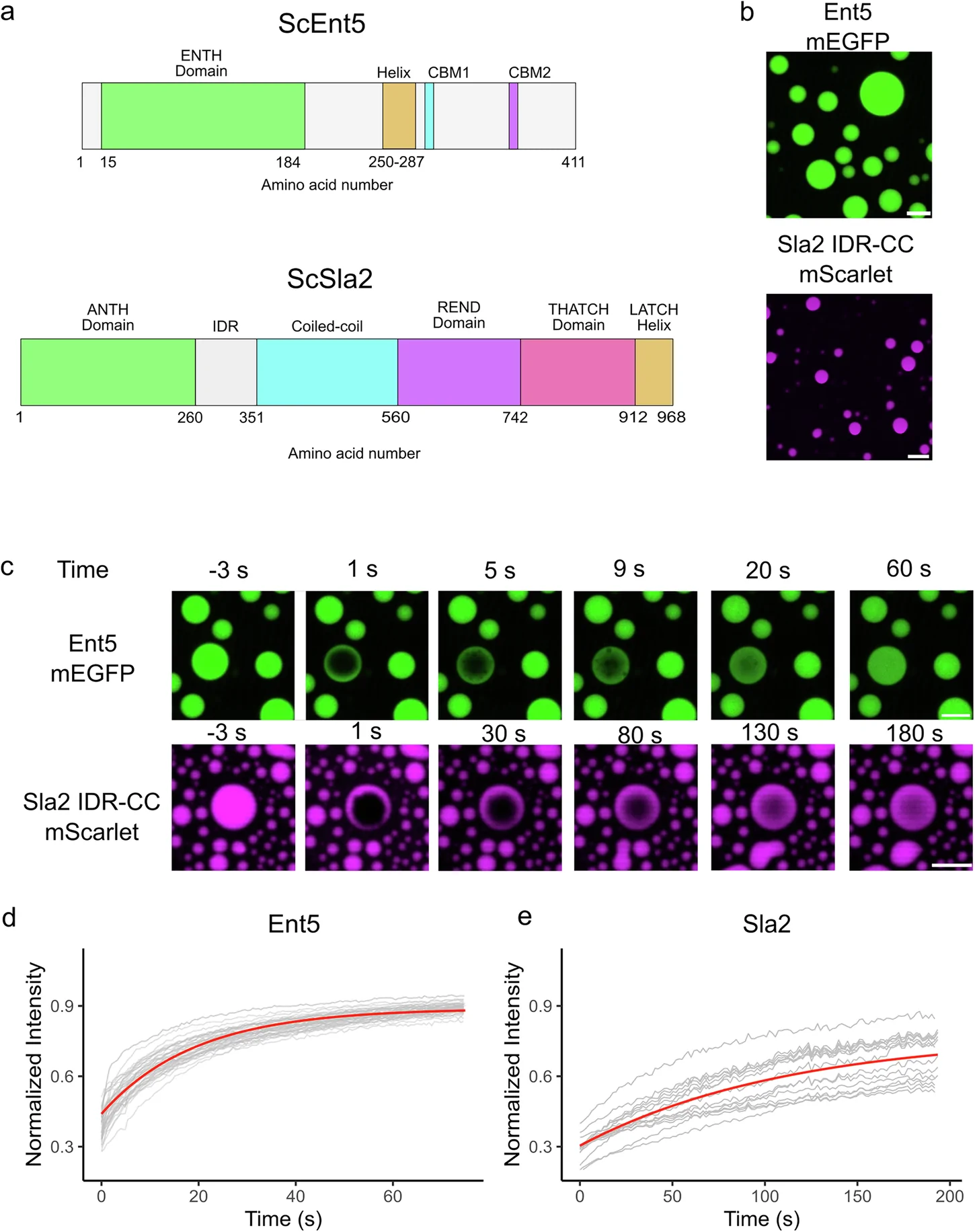
Ent5 and Sla2 form liquid condensates in vitro. **a** Domain architecture of Ent5 and Sla2 from *Saccharomyces cerevisiae*. Ent5 is formed by a membrane binding ENTH domain (15-184) and an IDR (211-411), which is composed of a predicted central helix (249-287), Clathrin Binding Motif (CBM) 1 (LIDL, 293-296) and 2 (LLDW, 355-358). Sla2 is composed of a membrane binding ANTH domain (1-260), IDR (260-351), Coiled-coil (351-560), REND domain (560-742), THATCH domain (742-912) and a LATCH helix (912-968). **b** Exemplar images of condensates at room temperature (21 °C) for Ent5 full-length N-terminally tagged with mEGFP (upper panel) and a truncated Sla2 (IDR and CC) tagged C-terminally with mScarlet (bottom panel). Ent5 droplets existed at 18 µM protein concentration in buffer (100 mM Tris pH 8, 20 mM NaCl, and 0.5 mM TCEP). Sla2 droplets existed at 25 µM protein concentration in the presence of PEG (2.5% PEG 8000, 30 mM HEPES pH 8, 150 mM NaCl, 0.5 mM TCEP). The white scale bar represents 5 µm. **c** Representative FRAP time course of droplets for Ent5-mEGFP (upper panels) and Sla2 IDR-CC-mScarlet (lower panels). Upper panels show confocal images of an Ent5-mEGFP droplet before photobleaching (–3 s) and at 1, 5, 9, 20, and 60 s after bleaching; lower panels show confocal images of a Sla2 IDR-CC-mScarlet droplet before photobleaching (−3  s) and at 1, 30, 80, 130, and 180 s after bleaching. Normalized intensity recovery plots for individual droplets are shown in grey, *n* = 45 for Ent5 (**d**) and n = 24 for Sla2 (**e**). A simple diffusion model fit is overlaid in red. Ent5 recovered with a time constant of (19.2 ± 0.2) s (**d**). Sla2 recovered with a time constant of (112.2 ± 2.4) s (**e**).

The initial screening step consisted of turbidity measurements using a microplate reader, an example of which is shown in Supplementary Fig. 2a. This assay provides a rapid and sensitive method to identify proteins capable of forming higher-order assemblies. Because turbidity reflects ensemble light scattering by particles whose size, number, and distribution can vary across replicate wells, these measurements inherently exhibit greater technical variability than spectroscopic readouts and were therefore used as an initial screening approach. However, because both LLPS and irreversible aggregation can produce increased light scattering, turbidity measurements alone are insufficient to distinguish between these states. We therefore complemented the screen with nanoDSF and static light scattering measurements, simultaneously monitoring protein unfolding (*T*_*m*_) and aggregate formation (*T*_agg_) (Supplementary Fig. 2b–e). These experiments revealed that Ent5 displayed behaviour consistent with reversible assembly formation, exhibiting cooperative unfolding in the presence of PEG with closely matching *T*_*m*_ and *T*_agg_ values. The observed turbidity therefore corresponds to a phase-separated state, consistent with LLPS as confirmed by fluorescence microscopy (Supplementary Fig. 2f). In contrast, no comparable signal was observed for Ent1 or Ent3 under the conditions tested. Finally, more detailed phase-separation analyzes were performed by systematically varying protein concentration, macromolecular crowding (PEG 8000), and ionic strength (Supplementary Fig. 2g,h). Together, these experiments identified Ent5 as capable of undergoing LLPS in vitro and therefore as a suitable candidate for further mechanistic investigation (Fig. 1).

**Fig. 2:**
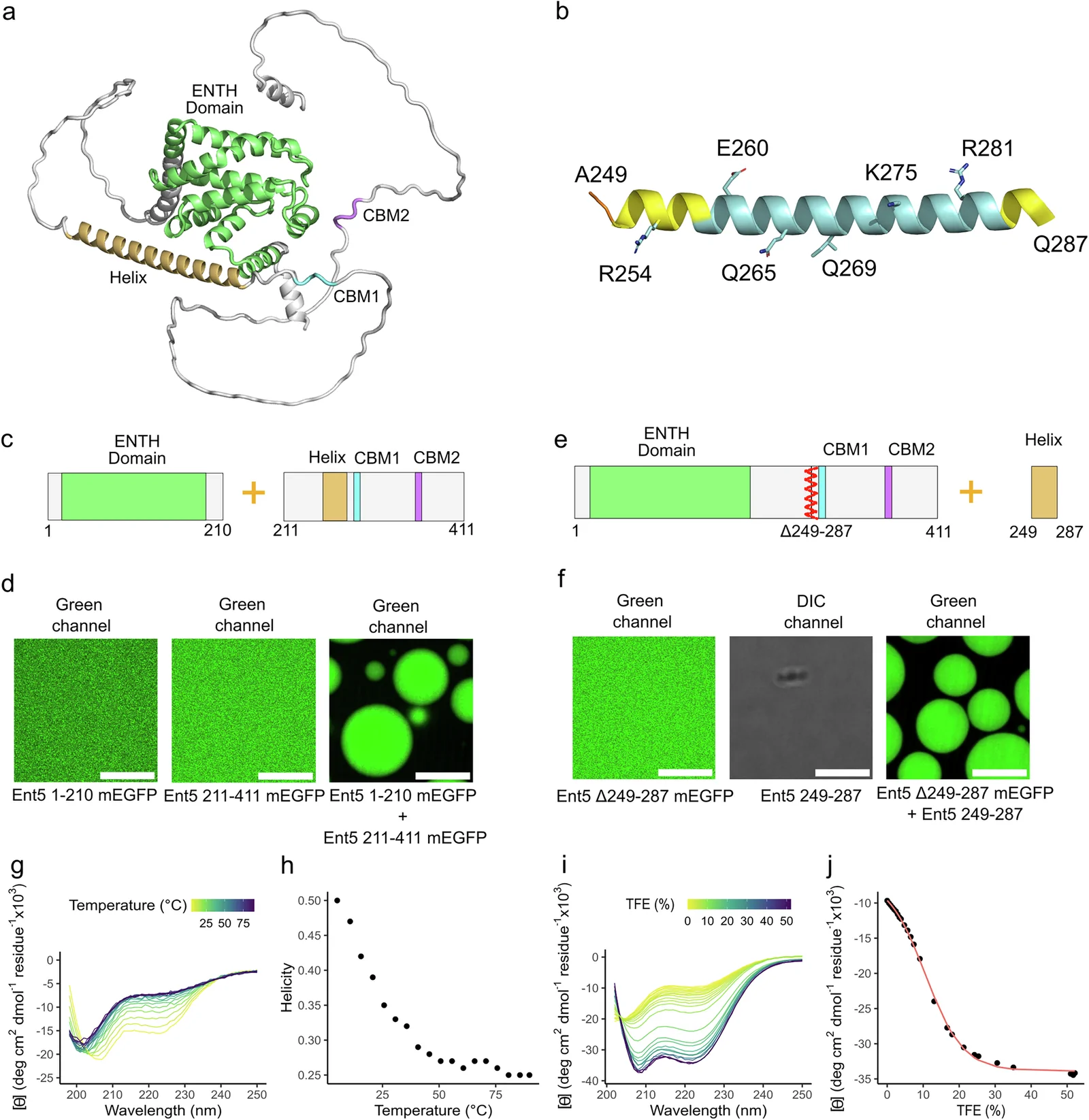
The Ent5 ENTH domain and IDR region containing a predicted helix (residues 249–287) cooperate to promote condensate formation. **a** AlphaFold model of Ent5: In green the ENTH domain, the predicted helix in orange, CBM1 in cyan and CBM2 in purple. **b** Zoom into the predicted Ent5 helix (249-287). In light blue the model confidence prediction is high (90 > pLDDT > 70) and lower in yellow (70 > pLDDT > 50). **c** Constructs used for the complementation experiment: ENTH domain (residues 1-210) and the IDR region (residues 211-411). **d** Ent5 ENTH domain (16 µM, left panel) and the IDR region (16 µM, middle panel) in 100 mM Tris (pH 8), 20 mM NaCl, and 0.5 mM TCEP at room temperature (21 °C) do not phase separate. When the two constructs are mixed (both at 16 µM) the sample forms condensates (right panel). **e** Constructs used for the complementation experiment: Ent5 Δ249–287 and Ent5 helix (249-287) peptide. **f** Ent5 Δ249–287 (16 µM, left panel) and Ent5 helix (249-287) (520 µM, right panel) do not phase separate (same buffer conditions as used in **d**). When the two constructs are mixed at these concentrations the sample forms condensates (right panel). **g** Circular Dichroism (CD) spectra of Ent5 helix (249-287) were recorded over a temperature ramp from 5 to 90 °C. **h** The percentage contribution of helical secondary structure, calculated from the molar ellipticity spectra using the Peptide Helicity module of ChiraKit^44^, is plotted against temperature. **i** Far UV CD spectra for the Ent5 helix (249-287) were recorded in the presence of TFE titrated from 0 to 52% (v/v) in 10 mM Tris (pH 7.5). **j** Molar ellipticity at 222 nm plotted against TFE percentage. Data were fitted with Eq. 2 using the Custom Analysis module of ChiraKit^44^ and the following parameters (and their corresponding percentage errors calculated as absolute values): θ_TFE_ = −6470.5 ± 532.7,θ_water_ = −33,867 ± 159.5, Δ*G*_water_ = −1.19 ± 0.07 kcal/mol, *m* = 11.23 ± 0.44. White scale bars (5 µm) in **d** and **f** indicate the scale for all microscopy images. **d**, **f** Representative of three independent experiments.

The AlphaFold3 model of Ent5 contains the predicted ENTH domain and a long IDR (see Figs. 1a and 2a). Interestingly, a well-predicted helix with an average predicted Local Distance Difference Test (pLDDT) of 74.36 is observed within the IDR and is not present in Ent3 (see Supplementary Fig. S3). Ent5 is found only in Phyla Ascomycota and Basidiomycota Dikarya (higher fungi, including *Saccharomyces cerevisiae*) and has been implicated in the trafficking of chitin synthase Chs3 (UniProt ID P29465)^27^. The 249-287 helix is located upstream of the clathrin-binding motifs CBM1 and CBM2^27^ (see Figs. 1a and 2b).

Ent5 undergoes phase separation in response to changes in salt concentration and the presence of crowding agents (Fig. 1, and Supplementary Fig. 2) at low micromolar concentrations. To confirm that the phase separation behavior of Ent5 is not due to the fluorescent tag, we performed differential interference Contrast imaging without the mEGFP fusion (see Supplementary Fig. 4). During visualization, frequent fusion events between individual droplets were observed, indicating their liquid nature. To assess the liquidity of the condensates, Fluorescence Recovery After Photobleaching (FRAP) was performed. Briefly, individual droplets within the field of view were selectively photobleached using a high-intensity laser, and fluorescence intensity was recorded over time to monitor recovery. The rate and uniformity of fluorescence return reflect the mobility of fluorescent molecules, consistent with a liquid-like state (Fig. 1b, c). The recovery kinetics were fitted using a simple diffusion model, with a fast recovery of fluorescence (Tau: ~19 s).

To identify the protein regions involved in LLPS, we designed two constructs of Ent5: one corresponding to the ENTH domain and one to the IDR containing the 249-287 helix (Fig. 2c). Our results indicate that only the full-length protein undergoes LLPS. Interestingly, mixing the ENTH domain and IDR constructs (at concentrations at which the full-length protein phase separates) results in LLPS, suggesting that the two domains complement each other (Fig. 2c). However, the individual constructs do not form droplets under the same conditions.

In order to test the existence of the AlphaFold-predicted helix experimentally, we performed Far-UV Circular Dichroism (CD) analysis on a peptide containing residues 249-287. Our results show that the peptide is primarily alpha-helical in solution (Fig. 2g–j). A temperature ramp experiment revealed a decrease in helical content, with a maximum of 50% helix formation observed at 5 °C. In the presence of Trifluoroethanol (TFE), helical regions were further stabilized, reaching 98% helical content. Proline substitutions in the peptide (see “Methods”), which disrupt alpha-helices, significantly reduced the helical propensity of the region (see Supplementary Fig. 5).

To test whether this inducible helix behaves as a sticker and is therefore responsible for LLPS, we generated a deletion mutant lacking the residues that adopt the helical conformation (Ent5 Δ249–287) (Fig. 2e). LLPS was abolished in Ent5 Δ249–287 but could be restored by supplementation with higher concentrations of the Ent5 (249–287) peptide.

Together, these results support a model in which residues 249–287 function as a sticker-like element within Ent5. Although neither the isolated Ent5 helix (249–287) peptide nor Ent5 Δ249–287 undergoes efficient phase separation individually, combining both components restores condensate formation. This activity in trans is consistent with a role for the helical region in increasing the effective interaction valency of Ent5 through transient intermolecular contacts that become productive when a sufficient local concentration is reached. However, the molecular identity of these contacts remains to be determined, and both heterotypic interactions with other regions of Ent5 and homotypic helix–helix interactions remain possible.

**Fig. 3:**
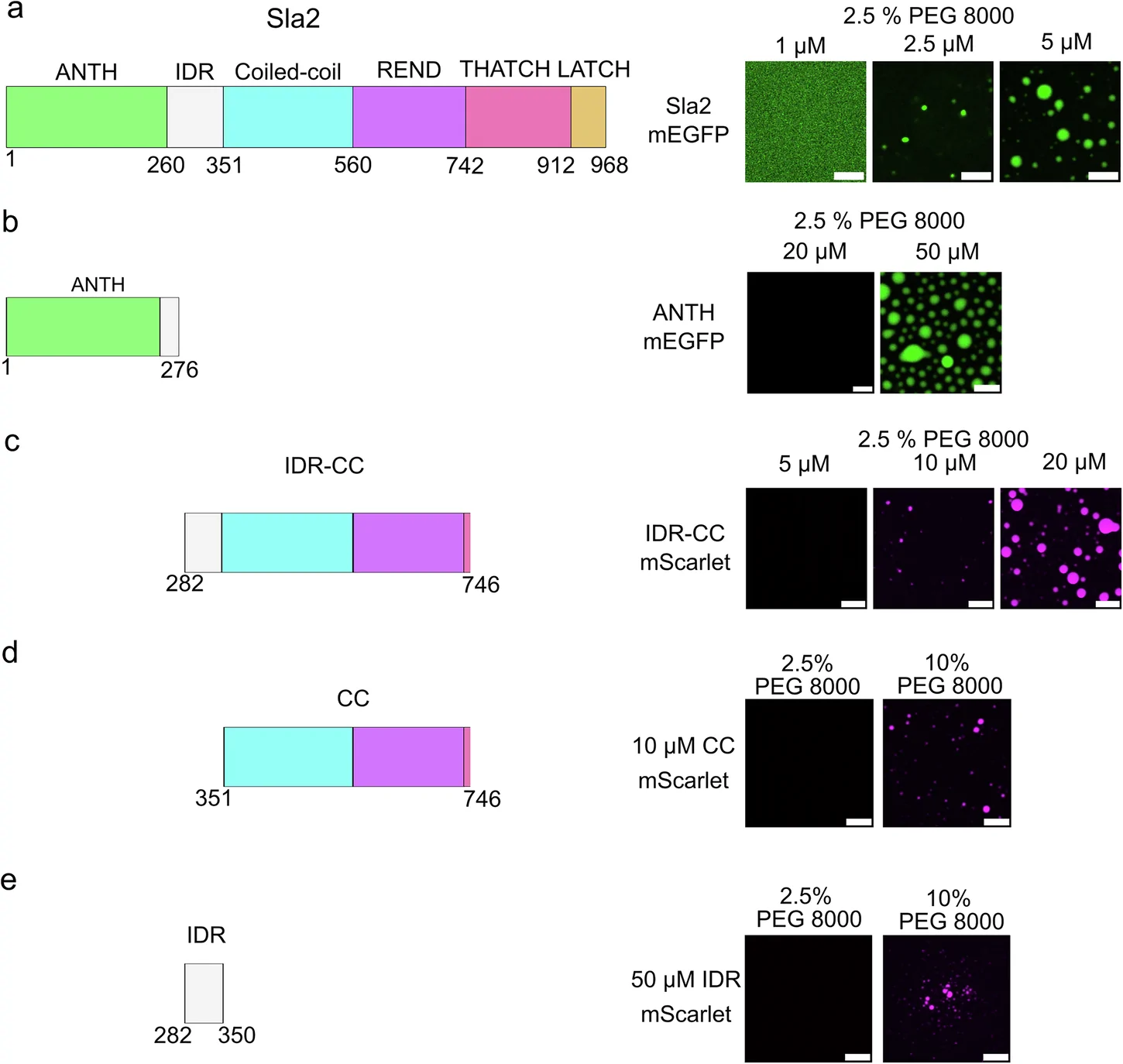
Condensate formation of Sla2 can be triggered by different regions. **a** Sla2-mEGFP forms condensates at 2.5 μM (smaller droplets) and at 5 μM (larger droplets). Experiments were performed at room temperature (RT, 21 °C) in 30 mM HEPES pH 8, 150 mM NaCl, and 0.5 mM TCEP. **b** Sla2 ANTH-mEGFP domain forms droplets at 50 μM in the presence of 2.5% (w/v) PEG 8000, in 30 mM HEPES pH 8, 150 mM NaCl, 0.5 mM TCEP at RT. **c** Sla2 IDR-CC–mScarlet (residues 282-746) forms condensates at 10 μM (smaller droplets) and at 20 μM (larger droplets) under the same PEG buffer conditions as in **b**. **d** Sla2 CC-mScarlet (residues 351-746) forms droplets in the presence of 10% (w/v) PEG 8000 at RT. **e** Sla2 IDR-mScarlet (residues 282-350) forms condensates at 50 μM in the presence of 10% (w/v) PEG 8000. White scale bars in **a**–**e** represent 5 µm. **a** to **e** Representative of three independent experiments.

Although Sla2 had a borderline LLPS prediction score, we prioritised it because of its central role in endocytosis and its extensive interaction network with epsins and other endocytic adaptors. Sla2 has previously been shown to be recruited to phase-separated Ede1 condensates in *S. cerevisiae*, and this has been proposed to depend on coiled-coil-containing regions of Ede1 and/or Sla2^14^. Here, we demonstrate that a full-length Sla2 construct forms condensates from ~2.5 µM in the presence of crowding agents (Fig. 3a).

To identify the protein regions responsible for LLPS, we designed truncated Sla2 constructs and tested them in vitro (Fig. 3). Each region was expressed with a C-terminal fluorescent tag (either mEGFP or mScarlet). The Sla2 ANTH domain formed condensates at 20–50 µM, while the Sla2 construct containing the IDR, coiled-coil, and REND regions (residues 261–746; hereafter referred to as IDR-CC) formed larger, fluid condensates at a lower threshold (~10 µM). The IDR construct (residues 261–350; hereafter referred to as IDR) and the construct containing the coiled-coil and REND regions (residues 351–746; hereafter referred to as CC) formed condensates in 10% (w/v) PEG 8000 at concentrations of 50 µM and 10 µM, respectively, suggesting that the coiled-coil and REND regions make a major contribution to condensate formation. Our results further indicate that both the IDR-CC and CC constructs form liquid condensates in the presence of 2.5% PEG 8000. These condensates exhibit uniform fluorescence recovery in FRAP experiments, confirming their liquid-like nature (Fig. 3c).

To further assess whether condensation of the Sla2 IDR-CC construct reflects reversible LLPS rather than progression towards an amyloid-like aggregated state, we monitored Thioflavin T (ThT) fluorescence over 7 days using two ThT concentrations in the presence or absence of molecular crowding (Supplementary Fig. 6). In contrast to the amyloid-forming control peptide Temporin-1CEa, Sla2 IDR-CC-mScarlet did not exhibit a time-dependent increase in ThT fluorescence. Consistent with this observation, negative-stain electron microscopy performed after 140 h incubation did not reveal fibrillar or amyloid-like structures (Supplementary Fig. 6e). Together, these results indicate that condensation of the Sla2 coiled-coil-containing region does not progress to detectable amyloid formation under the conditions tested and is consistent with the formation of dynamic condensates rather than irreversible aggregates.

Similar to the complementation observed between the ENTH domain and IDR in Ent5 (Fig. 2), addition of the Sla2 ANTH domain to the IDR-CC construct results in a synergistic enhancement of Sla2 condensation (Supplementary Fig. 7). The droplet-to-solution fluorescence intensity ratio (*I*_*D*_/*I*_*S*_) for Sla2 IDR-CC in the presence of the membrane-binding domain is 15 at 5 µM ANTH and 10 at 20 µM ANTH. These values are several-fold higher than those observed for Sla2 IDR-CC alone. Additionally, the ANTH domain *I*_*D*_/*I*_*S*_ increases from 5.5 to 7.4. In conclusion, the combination of the ANTH and IDR-CC regions exhibits an enhanced capacity for partitioning into condensates.

### Ent5 behaves as an LLPS driver

To investigate the biophysical properties of Ent5 in LLPS, we analyzed its kinetics of condensation in vitro. Mass photometry was used to monitor the presence of nanoclusters in equilibrium at low (5 mM) and high (100 mM) NaCl concentrations (see Supplementary Fig. 8). Ent5 monomeric peaks (~91–97 kDa) were observed under all conditions. In addition, we detected a population of higher‑molecular‑weight species (>200 kDa) for the wild‑type construct specifically under low‑salt conditions. Although these assemblies represent only a low‑abundance fraction of the total population and should not be overinterpreted quantitatively, their appearance was reproducible and correlated with conditions that promote condensate formation. In contrast, Ent5 Δ249-287 showed reduced signal in this higher‑mass range.

To further investigate the transition from monomers to higher oligomeric forms and to understand the kinetics of this assembly process, we performed Stopped-Flow light scattering and Stopped-Flow Small-Angle X-ray Scattering (SAXS) experiments (see Supplementary Fig. 9). We aimed to quantify the speed of this transition and identify potential growth phases during nanocluster formation. We initiated the assembly by rapidly mixing Ent5, initially stabilized in a high-salt buffer, with a low-salt buffer to trigger the formation of larger molecular weight (MW) species. Both light scattering and SAXS are suitable for probing nanocluster formation, covering different size regimes of nanoclusters. Light absorption measurements at 600 nm (Supplementary Fig. 9a) revealed a biexponential kinetic profile, characterized by a rapid initial phase (τ₁ = 108 ± 7 ms) followed by a slower secondary phase (τ₂ = 843 ± 42 ms). This suggests that the assembly process proceeds through at least two distinct kinetic steps. To further characterize the emergence of nanoclusters, we employed SAXS experiments. The SAXS trace in the Guinier region displayed complex behavior, which could not be fitted with a simple exponential curve. However, a qualitative analysis indicated a half-time of approximately 200 ms, suggesting a rapid and structured growth phase (Supplementary Fig. 9b).

Given the central role of multivalent interactions in phase separation, we next examined how Ent5 engages clathrin. Ent5 contains two predicted clathrin-binding motifs (CBM1 and CBM2; Fig. 1a), one of which (CBM1) has previously been shown to bind the N-terminal domain of clathrin heavy chain (NTD-CHC) with low micromolar affinity^15^. To determine whether CBM2 also functions as a clathrin-binding motif, we solved the crystal structure of CBM2 in complex with *S. cerevisiae* NTD-CHC. The CBM2 peptide engages simultaneously with the three canonical binding sites: Clathrin box, Arrestin box, and W-box (Supplementary Fig. 10, and Table 2). The overall backbone binding mode is conserved between CBM1 and CBM2, although CBM2 engages NTD-CHC through distinct side-chain interactions. This demonstrates that the overall recognition mode is maintained despite differences in the underlying molecular contacts, highlighting the adaptability of Ent5 clathrin-binding motifs and providing a structural basis for multivalent clathrin engagement. In the Clathrin box site (Asn89), the bulky Trp residue at position +4 cannot occupy the Leu+4 pocket as in CBM1; instead, it stacks against Phe91 through a π–π interaction and forms a hydrogen bond between its indole nitrogen and Asp−1, likely stabilizing its conformation. In the Arrestin box, Trp+4 occupies the same site as Leu+4 in CBM1, enabled by a repositioning of Arg235, which also permits a cation–π interaction between Arg235 and Trp+4. In the W-box site, CBM2 Trp+4 forms an additional hydrogen bond with Gln175, an interaction absent in CBM1, replacing an internal Leu+4–Leu+1–Pro−2 contact. Together, these results establish that Ent5 contains two functional clathrin-binding motifs capable of engaging NTD-CHC through distinct but conserved interaction modes. The presence of two clathrin-binding sites expands the interaction valency of Ent5 and provides a structural framework for multivalent clathrin engagement. In the context of our LLPS model, such multivalency could facilitate clathrin recruitment and retention within Ent5-containing condensates.

**Table 2:** X-ray data collection and refinement statistics

	NTD-CHC in complex with Ent5-CBM2 (9S6D)
Data collection	
Space group	C121
Cell dimensions	
*a*, *b*, *c* (Å)	96.34 129.88 74.53
α, β, γ (°)	90.0 96.67 90.0
Resolution (Å)	35.56–1.7 (1.761–1.7)
*R*_sym_ or *R*_merge_	0.07639 (1.21)
*I* / σ*I*	12.88 (1.69)
Completeness (%)	97.47 (96.15)
Redundancy	7.1 (7.2)
Refinement	
Resolution (Å)	1.7
No. reflections	97,197 (9569)
*R*_work_ / *R*_free_	0.1830 / 0.2114
No. atoms	6471
Protein	6080
Water	391
*B*-factors	
Protein	42.42
Water	42.69
R.m.s. deviations	
Bond lengths (Å)	0.011
Bond angles (°)	1.84

Statistics for the highest-resolution shell are shown in parentheses.

**Fig. 4:**
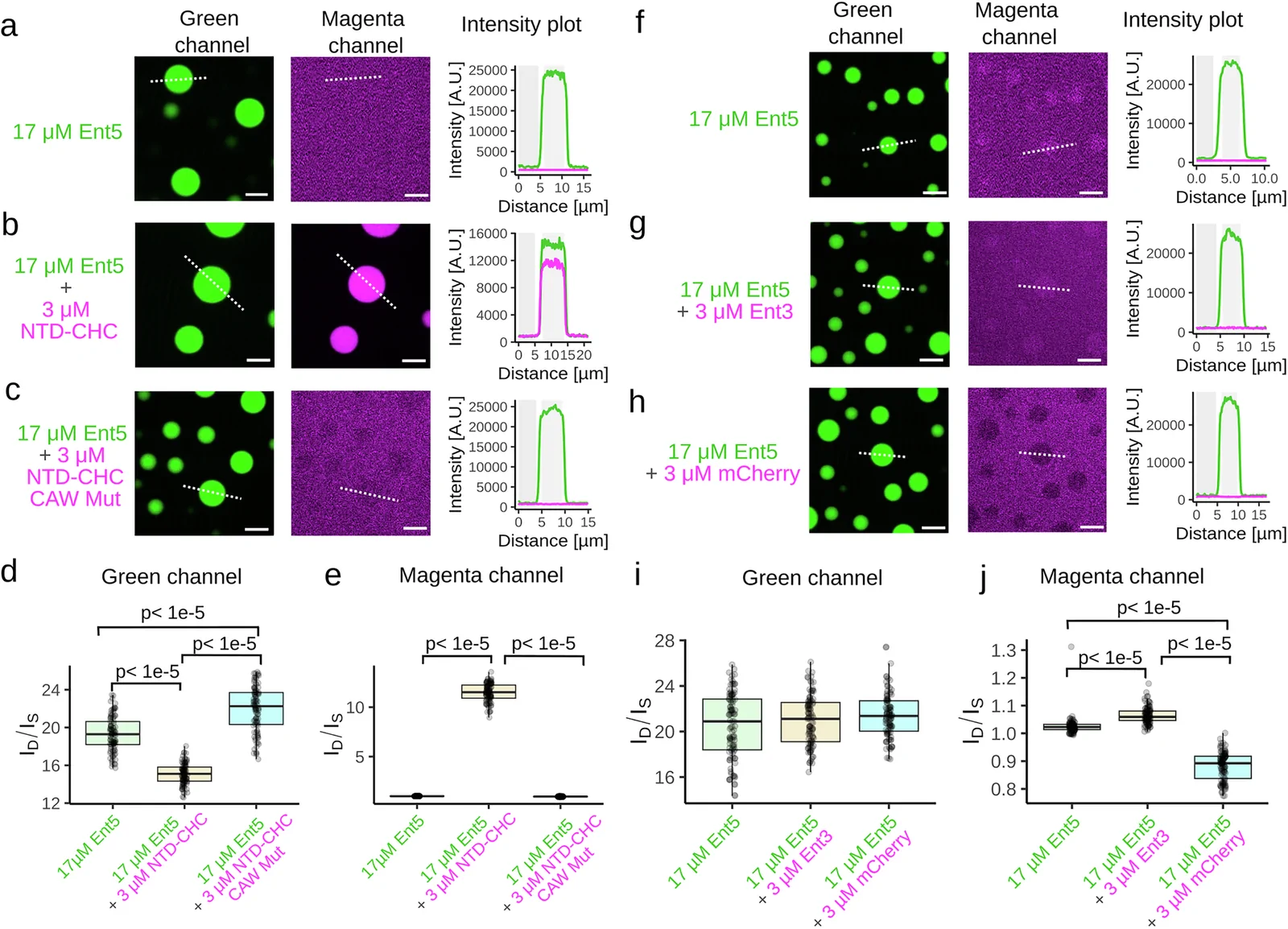
Recruitment of clathrin heavy chain and Ent3 to Ent5 droplets. **a**–**e** Recruitment of Clathrin Heavy Chain. **a** 17 μM Ent5 imaged in the green channel (488 nm excitation) phase separates without any detectable signal in the magenta channel (561 nm excitation). **b** 17 μM Ent5 in the presence of 3 μM NTD-CHC-mScarlet. **c** 17 μM Ent5 in the presence of 3 μM of NTD-CHC CAW mutant (Clathrin, Arrestin and W-Box mutant, incapable of binding CBM1 and CBM2 regions from Ent5, see “Methods”). In each microscopy panel, a representative image (*n* = 30) is shown with a central line drawn to quantify the difference in protein concentration between the droplet (I_D_, intensity in the droplet) and the surrounding solvent (I_S_, intensity in the solvent). **d**, **e** Intensity ratios (I_D_/I_S_) were quantified for the green channel (Ent5; panel **d**) and the magenta channel (NTD-CHC; panel **e**) under three conditions: 17 μM Ent5-mEGFP, 17 μM Ent5-mEGFP with 3 μM NTD-CHC-mScarlet, and 17 μM Ent5-mEGFP with 3 μM NTD-CHC CAW. Statistical tests were performed using two-sided Yuen trimmed means, corrected for multiple comparisons by the Holm–Bonferroni method; “*p*” denotes the corrected *p*-value. **f**–**j** Recruitment of Ent3. **f** 17 μM Ent5 imaged alone does not show signal in the magenta channel. **g** 17 μM Ent5 in the presence of 3 μM Ent3-mCherry. **h** A control experiment showing 17 μM Ent5 and 3 μM mCherry shows no increase in the magenta signal. Intensity ratios (*I*_*D*_/*I*_*S*_) were quantified for the green channel (Ent5-mEGFP; **i**) and the magenta channel (Ent3-mCherry or mCherry; **j**) under three conditions: 17 μM Ent5-mEGFP alone, 17 μM Ent5-mEGFP with 3 μM Ent3-mCherry, and 17 μM Ent5-mEGFP with 3 μM mCherry. Statistical tests were conducted using two-sided Yuen trimmed means, corrected for multiple comparisons by the Holm–Bonferroni method. White scale bars in all microscopy panels represent 5 μm. Buffer: 100 mM TRIS, 20 mM NaCl, 0.5 mM TCEP. *n* = 99 (number of droplets) on three independent experiments.

Proteins can be classified as drivers or clients in LLPS depending on their role in initiating phase separation and recruiting other partners. Since Ent5 has two active CBMs and can undergo phase separation under multiple in vitro conditions, we hypothesize that it acts as a driver of LLPS. We have then tested Ent5’s capacity for recruiting clients (see Fig. 4). We performed recruitment experiments following Ent5-mEGFP in the presence of NTD-CHC-mScarlet and we observed that the second one is selectively recruited to Ent5 droplets (see Fig. 4b). The partitioning of Ent5 into droplets is reduced in the presence of NTD-CHC, with the ID/IS decreasing from 19.24 to 15.06 (Fig. 4d), consistent with Ent5 remaining enriched in the dense phase but partitioning less strongly into droplets when NTD-CHC is present. We have then performed the same experiment introducing mutations to the Clathrin-, Arrestin- and W-Box boxes of NTD-CHC (CHC CAW mutant, see Methods). Because Ent5 cannot bind NTD-CHC CAW through its CBM1 and CBM2 regions, NTD-CHC CAW is not recruited to the droplets (see Fig. 4c). Our results suggest that NTD-CHC is recruited into Ent5 droplets by a well-defined, high-affinity interaction between the NTD-CHC and the CBMs in Ent5, yielding strong and efficient partitioning.

Ent3 is an epsin involved in the recruitment of Gga and AP-1 proteins during trans-Golgi network trafficking^26^ and does not contain a CBM. In order to test Ent5’s capacity as a LLPS driver, we performed fluorescence microscopy colocalization experiments with Ent5-mEGFP and Ent3-mCherry to test the recruitment of Ent3 to Ent5 droplets (see Fig. 4f–j, and Supplementary Fig. 11). Our results indicate that Ent5 increases Ent3 concentration in the droplet phase (Fig. 4j) but Ent3 does not change the concentration of Ent5 (Fig. 4i). Actually, the recruitment of Ent3 to Ent5 droplets depends on the Ent5 concentration, which is consistent with Ent3 being a LLPS client (Supplementary Fig. 11a). As a control, we show that mCherry alone does not colocalize within Ent5 droplets, meaning it does not behave as a LLPS client (Supplementary Fig. 11b). In contrast to what has been observed for NTD-CHC, Ent3 partitions into Ent5 droplets but does so via an unknown mechanism.

### Sla2 behaves both as an LLPS driver and client

**Fig. 5:**
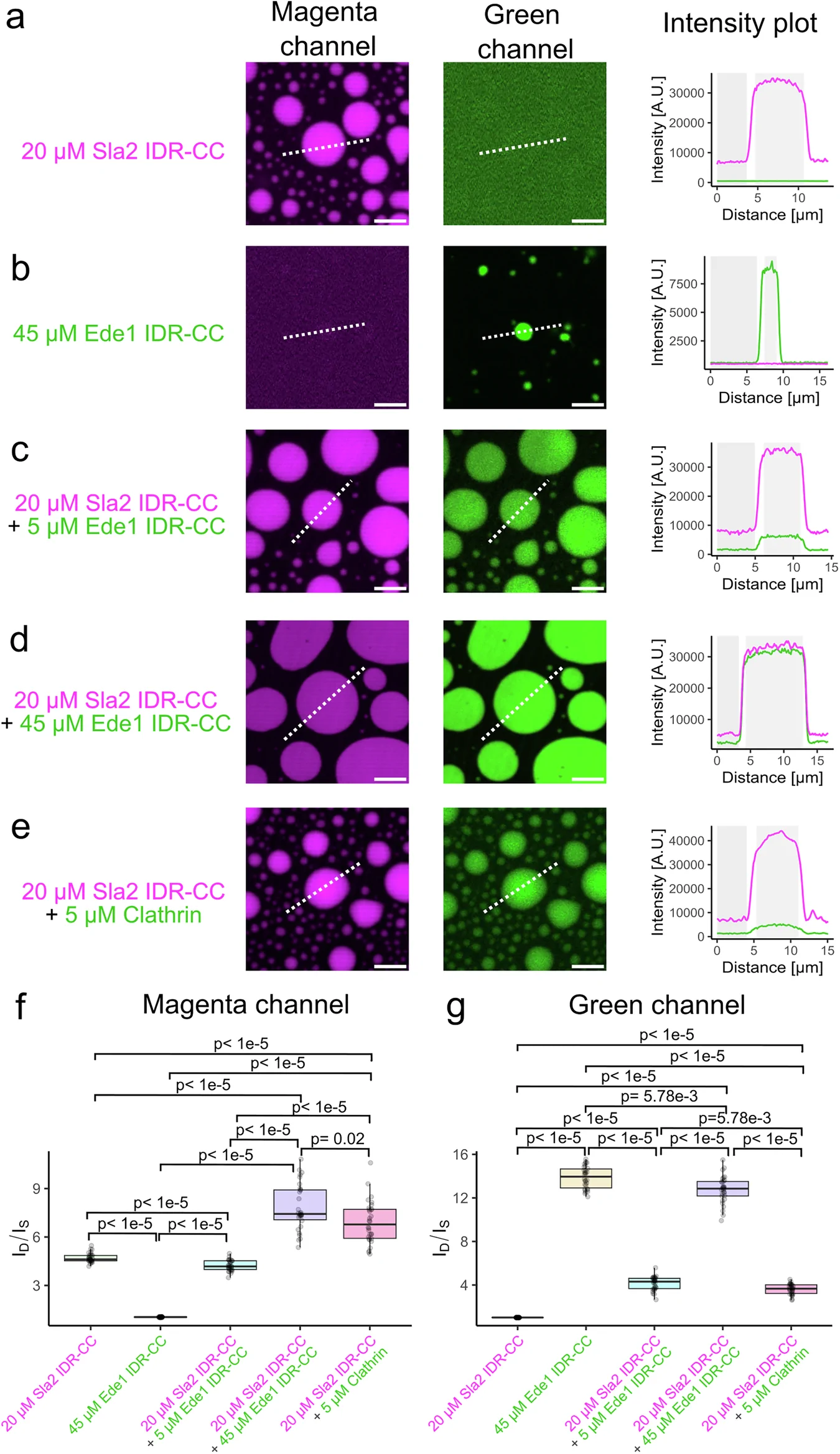
Co-localization and recruitment of Sla2 with Ede1 and Clathrin in condensates. **a**–**e** Representative droplets for each sample are shown for the magenta (left panels) and the green (middle panels) channels. All experiments were performed in the presence of 2.5% (w/v) PEG 8000. Measurements across the white dotted lines are used to quantify fluorescence intensities between the droplet (*I*_*D*_) and the surrounding solvent (*I*_*S*_). Intensity profiles are plotted on the right panels. Images correspond to Sla2 IDR-CC-mScarlet (**a**, **c**–**e**) for Ede1-mEGFP (**b**–**d**) or Clathrin-mEGFP (**e**). **f** Average intensity ratios (I_D_/I_S_) measured from the magenta channel for *n* = 30 droplets. Statistical tests were conducted using two-sided Yuen trimmed means, corrected for multiple comparisons by the Holm–Bonferroni method; “*p*” denotes the corrected *p*-value. **g** Average intensity ratios (*I*_*D*_/*I*_*S*_) measured from the green channel for *n* = 30 droplets. Statistical tests were conducted using two-sided Yuen trimmed means, corrected for multiple comparisons by the Holm–Bonferroni method; “*p*” denotes the corrected *p*-value. White scale bars in all microscopy panels represent 5 µm.

Through the same analysis as done on Ent5 with client proteins, we have performed Sla2 experiments to test the interaction with protein partners in the droplet phase. Ede1, homologous to *Hs*Eps15, has been described as a driver in phase separation and its central region, residues 366-900 (Ede1 IDR-CC), has been implicated in the formation of condensates at ~5 µM protein concentration^28^. We therefore tested the co-localization of Ede1 IDR-CC and Sla2 IDR-CC in the presence of 2.5% (w/v) PEG 8000 (see Fig. 5a–d).

Under our experimental conditions, yeast Ede1 IDR-CC did not undergo detectable phase separation at 5 µM, whereas robust condensate formation was observed at approximately 45 µM. Although 5 µM Ede1 alone remained soluble, addition of 20 µM Sla2 IDR-CC resulted in recruitment of Ede1 into Sla2 condensates (ID/IS ≈ 4), indicating that Sla2 can recruit Ede1 under conditions where Ede1 alone does not undergo detectable phase separation. Conversely, Sla2 IDR-CC alone formed condensates at 20 µM with an ID/IS ratio of 4.7. When mixed with 45 µM Ede1 IDR-CC, the partitioning of Sla2 increased to an ID/IS ratio of 7.8, whereas Ede1 exhibited only a small but statistically significant decrease in partitioning. Together, these findings suggest that the relative scaffold contribution within mixed condensates is concentration-dependent.

It has been shown that Sla2 binds CLC through two binding sites^16^. Utilizing the full-length CLC and CHC distal leg (residues 1172-1572) heterodimer construct conjugated to mNeonGreen (named the “Clathrin” construct), we have measured the recruitment of Clathrin to Sla2 IDR-CC–mScarlet droplets (see Fig. 5e). The *I*_*D*_/*I*_*S*_ of Sla2 is significantly increased from 4.7 to 6.9 (Fig. 5f) and the *I*_*D*_/*I*_*S*_ of Clathrin from 1.01 to 3.6 (Fig. 5g), indicating recruitment of Clathrin to Sla2 droplets.

### Ent5 and Ent1/Sla2 condensation is promoted by the membrane environment

**Fig. 6:**
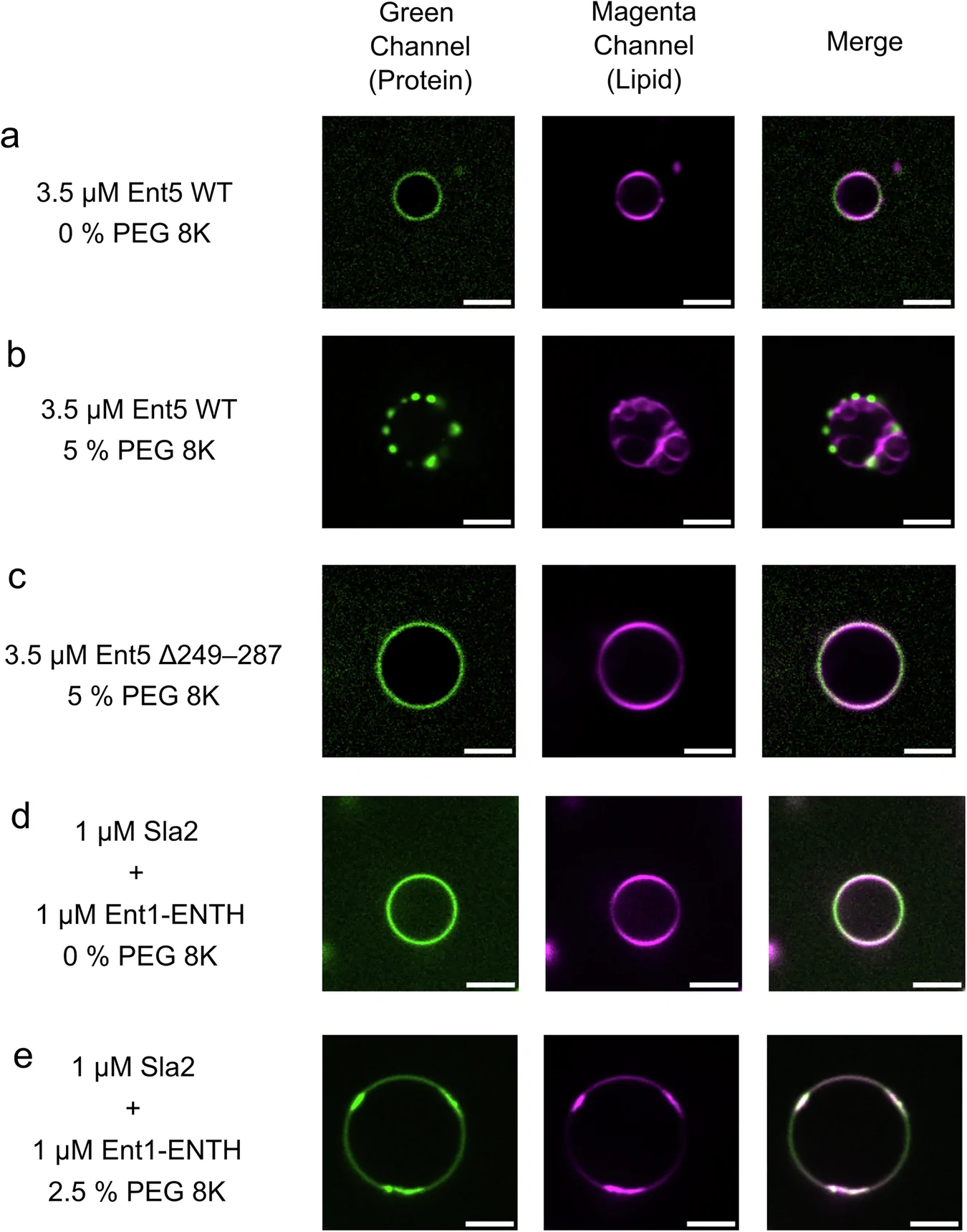
Ent5 and Sla2 form membrane-associated assemblies on GUVs containing signaling lipids. **a**–**c** Preformed GUVs, containing 8% PI(3,5)P_2_, were incubated at 21 °C with 3.5 μM Ent5-mEGFP. Fluorescence images were acquired in two channels: the protein is visualized in the green channel (mEGFP) and the lipid in the magenta channel (Cy5.5). Three conditions are shown: **a** 0% (w/v) PEG 8000, **b** 5% (w/v) PEG 8000 and **c** 3.5 μM Ent5 Δ249-287-mEGFP in the presence of 5% (w/v) PEG 8000. **d**, **e** Preformed GUVs containing 8% PI(4,5)P_2_ were incubated at 21 °C with a mixture of 1 μM Sla2–mEGFP and 1 μM Ent1-ENTH. Two conditions are displayed: **d** 0% (w/v) PEG 8000 and **e** 2.5% (w/v) PEG 8000. White scale bars in all images represent 5 μm.

We have used Giant Unilamellar Vesicles (GUVs) as a vehicle for determining the recruitment of both Ent5 and Sla2 condensates to membrane environments (see Fig. 6). In the case of Ent5, GUVs were prepared with 8% PI(3,5)P_2_ and 0.1% Cy5.5-labelled PE (1,2-distearoyl-sn-glycero-3-phosphoethanolamine-N) to visualize the membrane in fluorescence microscopy. 5% PEG 8000 was chosen for the condensate-inducing variable, as salt modification is not possible due to destroying the GUVs when changing the molality of the external solution as opposed to the internal sucrose solution. Our results show that Ent5 forms localized larger patches corresponding to membrane-associated condensates at 3.5 µM protein concentration in the presence of the crowding agent (see Fig. 6b). This phenotype is not observed for similar concentrations of Ent5 Δ249-287 mutant in the presence of 5% PEG 8000 (see Fig. 6c), displaying a behavior similar to the WT Ent5 in the absence of a crowding agent (see Fig. 6a). Therefore, our results suggest that the Ent5 helix (249-287) is required for condensation once Ent5 is bound to the membrane. Notably, membrane-associated condensates were also observed at an Ent5 concentration of 500 nM (Supplementary Fig. 12), consistent with membrane recruitment increasing the local surface density of proteins in two dimensions and thereby lowering the apparent concentration threshold for phase separation.

Sla2 binds PI(4,5)P₂ when forming a complex with the epsins Ent1 and Ent2^29^ through their ANTH and ENTH domains, respectively, forming a high-affinity complex in the nanomolar range^30^ that induces membrane deformation^31^. In order to test the formation of condensates onto the membrane environment, GUVs were prepared containing 8% PI(4,5)P_2_ and 0.1% Cy5.5. We mixed full-length Sla2-mEGFP with equimolar amounts of Ent1 ENTH domain at a protein concentration of 1 µM in the GUV mix. At the protein concentrations used in this study, Sla2-mEGFP was uniformly recruited to the membrane without detectable membrane remodeling. In our experiments, we observed uniform recruitment of Sla2-mEGFP to the membrane, but no clustering (see Fig. 6d). In the presence of crowding agents, local condensates of increased protein concentration are seen, but no tubular deformation of the membrane (see Fig. 6e).

### Removal of the Ent5 249-287 helix prolongs the lifetime of Gga2-dependent coating

**Fig. 7:**
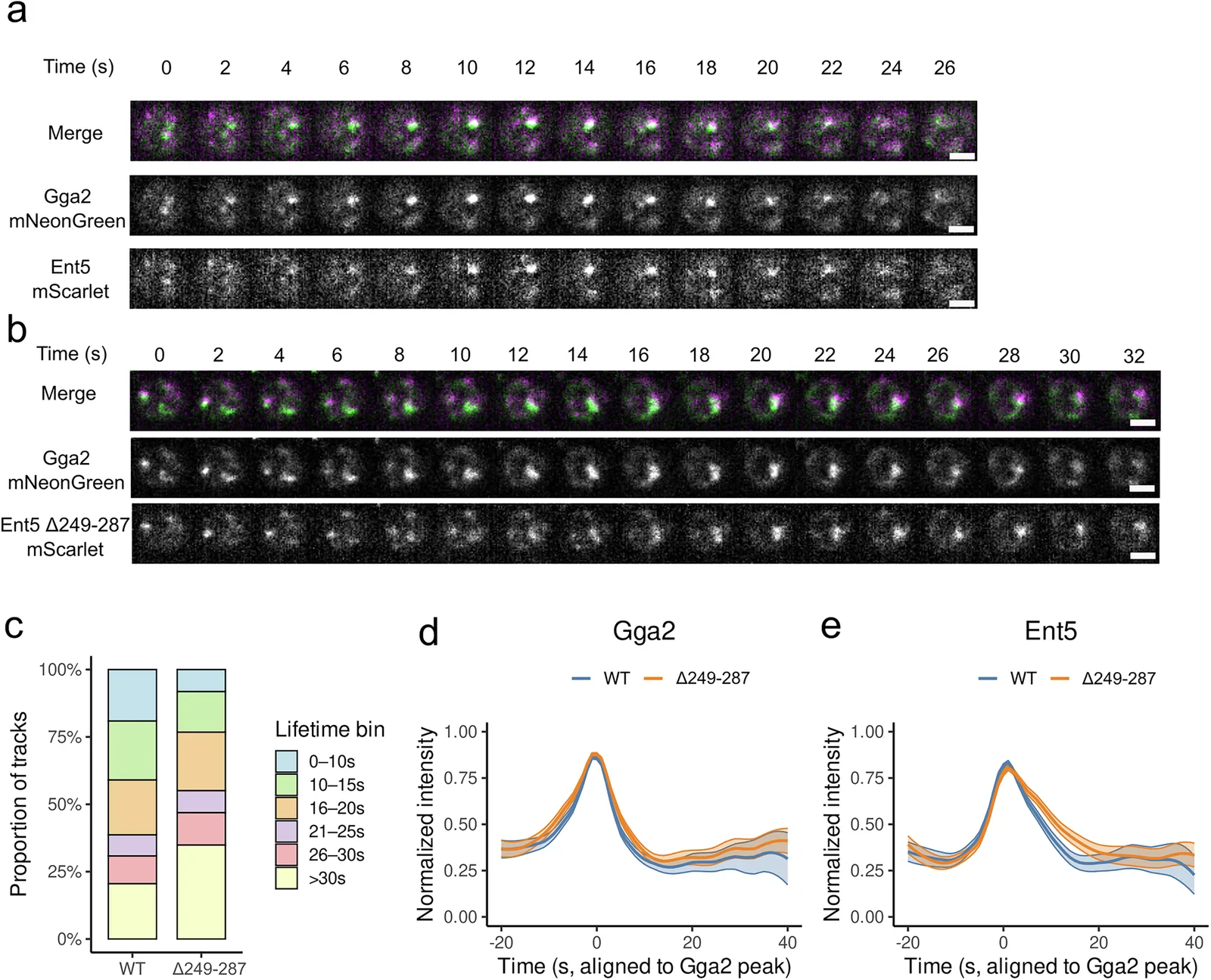
Removal of the Ent5 249-287 helix prolongs Gga2-associated coating events in *S. cerevisiae*. Representative time-lapse montages centered on tracked spots for WT (**a**) and Ent5 Δ249–287 (**b**). Gga2 (Green), Ent5 (Magenta); merged composites shown. Scale bar, 2 µm. **c** Proportional stacked bar plots showing the distribution of track lifetimes across six duration bins (0–10, 10–15, 16–20, 21–25, 26–30, >30 s) for WT (left) and Ent5 Δ249–287 (right). Statistical comparison using a χ² test of independence on the full contingency table: χ² = 54.43, d = 5, *p* = 1.71 × 10⁻^10^. Mean normalized intensity profiles of Gga2 (**d**) and Ent5 (**e**) for WT (blue) and Ent5 Δ249–287 (orange). Traces are aligned to the Gga2 intensity peak (*t* = 0), with each curve showing the LOESS fit to the dataset mean (solid line) and 95% confidence interval (shaded region). Individual trajectories were not smoothed prior to averaging. Sample sizes: WT, *n* = 639; Ent5 Δ249–287, *n* = 525.

We found that a dynamic helix (residues 249-287) of Ent5 behaves as a *sticker* for LLPS in vitro. To test its functional relevance in vivo, we generated a *S. cerevisiae* strain with an Ent5 Δ249-287 construct and asked whether the removal of the inducible helix would alter adaptor-mediated trafficking. To read out trafficking dynamics, we quantified the lifetimes of Gga2 and Ent5 puncta together as a proxy. For this, we developed a dual-camera, 3D fluorescence microscopy assay in which endogenous Gga2 is C-terminally tagged with mNeonGreen, and Ent5 is supplied from a plasmid as either WT or Ent5 Δ249-287, tagged with mScarlet (see Fig. 7).

Across movies, the Ent5 Δ249-287 mutant prolongs Gga2-positive events. This shift in the average reflects a redistribution of event durations when all tracks are pooled: the fraction of short events (0–10 s) decreases, while long events (>30 s) increase in Ent5 Δ249-287 relative to WT (Fig. 7c; χ² = 54.43, df = 5, *p* = 1.71 × 10⁻¹⁰; nWT = 639, nEnt5 Δ249-287 = 525; examples: 0–10 s WT 19.1% → ΔEnt5 Δ249-287 8.2%; >30 s WT 20.5% → Ent5 Δ249-287 34.9%). Peak-aligned overlays of Gga2 (LOESS fit shown as a solid line, 95% CI as shaded area) reveal similar assembly–disassembly kinetics in both genotypes (Fig. 7d). In addition, recruitment of Ent5 to Gga2-positive structures is not detectably altered in the Ent5 Δ249–287 mutant, indicating that the initial interaction with Gga2 is preserved. Consistent with this, the Gga2-binding region of Ent5 (residues 327–337) is spatially distinct from the deleted helical segment (residues 249–287), suggesting that the Δ249-287 mutation does not directly disrupt the canonical interaction interface.

For Ent5 dynamics, Ent5 Δ249–287 also increases the average event lifetime (Fig. 7e; LOESS overlays, nWT = 639, nEnt5 Δ249–287 = 525), while representative montages show comparable event morphology in both conditions (Fig. 7a, b). Together, these results indicate that removal of the Ent5 249-287 helix selectively extends the duration of Gga2-marked trafficking events and shifts the population toward longer-lived assemblies. While Gga2 dynamics remain largely unchanged, the data are consistent with impaired release of Ent5 from Gga2-positive structures rather than altered recruitment.

## Discussion

Our experimental screening revealed that predictions for phase separation can be misleading. These predictions are based primarily on the presence of IDRs to suggest LLPS. Despite computational predictors suggesting a potential role in LLPS, we did not observe condensate formation for Ent1 or Ent3. Consistently, these epsins showed no signs of phase separation in our chemical screening assays. Supporting this, Sarkar et al.^28^ reported that Epsin1 not only fails to form condensates but may also destabilize the condensation of Eps15.

Another observation is that predictions for phase separation are sequence-based, with algorithms taking into account the local residue context but not structural information. Therefore, while the NTD-CHC domain is not predicted to undergo LLPS, the CLC, which contains an IDR, is predicted to spontaneously undergo LLPS. However, we do not know how the assembled triskelions would behave in vivo. Adaptors bound to them seem to act as more intuitive switches for condensate formation.

Ent5 demonstrated clear phase separation in our chemical screening, requiring both its helical 249-287 region and the ENTH domain. Notably, the full IDR complements at a lower concentration than the isolated 249-287 peptide, indicating that the larger IDR, acting as a spacer, contributes significantly to phase separation.

Our findings suggest that Ent5 may act as a driver of phase separation in endosomal trafficking given its capacity for recruiting NTD-CHC and, to a lesser degree, Ent3. Importantly, Ent5 is unlikely to act in isolation in vivo. Endocytic and trafficking adaptors operate within highly interconnected interaction networks, and heterotypic interactions with partner proteins are likely to contribute substantially to condensate formation and regulation. The cooperative behaviour we observe between the ENTH domain and the C-terminal region of Ent5, together with the efficient recruitment of clathrin into Ent5 condensates, supports a model in which condensates emerge from multiple weak interactions among adaptors, coat proteins, and membranes rather than from the self-association of a single component. Future studies will be required to define how additional adaptor proteins contribute to the assembly, composition, and dynamics of these condensates.

Our findings suggest that the scaffold driving Sla2–Ede1 condensates is concentration-dependent. At low Ede1 concentrations, Sla2 can recruit Ede1 into pre-existing condensates, whereas at higher Ede1 concentrations, Ede1 acts as the dominant scaffold and enhances Sla2 partitioning. This behaviour is consistent with the temporal organization of the yeast endocytic machinery, in which Ede1 is among the earliest proteins recruited to nascent endocytic sites, while Sla2 arrives later during coat assembly^14,32^. Dynamic shifts in the relative abundance of these proteins may therefore contribute to changes in condensate composition and scaffold dominance during endocytic site maturation.

Importantly, these observations do not imply that Sla2 functions solely as a recruited client. Rather, our data indicate that Sla2 itself possesses intrinsic condensate-forming properties that could enable it to actively shape the composition and material properties of endocytic assemblies. We show here that individual regions of Sla2 are capable of phase separation. The central coiled-coil and REND region behave as a sticker, and complementation experiments indicate inter-protein interactions that are further enhanced in the context of the full-length protein. This suggests a dual client/driver role for Sla2, potentially tied to its critical function in regulating the Pan1/End3/Sla1 complex^33,34^. If this role extends to other adaptors and regulators, it may facilitate the recruitment of actin polymerization and regulatory machinery during endocytosis.

Our results further underscore the importance of discrete structural elements in driving phase separation. In Ent5, we identified a dynamic helix within the IDR that functions as a sticker. Although the isolated 249–287 peptide does not undergo phase separation on its own, it can restore condensation when added to Ent5 Δ249–287, indicating that the helix acts cooperatively with the surrounding disordered region and the ENTH domain. This behaviour is consistent with the helix contributing weak or transient interactions that become productive at sufficient local concentrations, supporting its role as a sticker-like element rather than as an autonomous condensation module. The molecular nature of these interactions remains unresolved and may involve homotypic helix–helix contacts, heterotypic interactions with other regions of Ent5, or both. Membrane recruitment is likely to further enhance local protein concentration and facilitate condensate formation. The synergy between inducible secondary structure and flexible spacer regions appears to be a recurring principle in endocytic adaptors.

In Sla2, by contrast, the central coiled-coil-REND-containing region appears to make a major contribution to condensate formation. Our truncation and complementation analyzes indicate that this region provides interaction valency that promotes condensation, particularly under crowding conditions and in combination with adjacent domains. This observation is consistent with emerging evidence that coiled-coil-containing regions can contribute to LLPS through multivalent intermolecular interactions, rather than functioning solely as static oligomerization motifs^10,35^. Unlike the Ent5 helix region (249-287), the Sla2 coiled-coil-REND-containing region is sufficient to form droplets under crowding conditions, and its condensation is further enhanced when combined with neighboring domains. Together, these findings suggest that endocytic adaptors can employ distinct structural features as determinants of condensate formation. However, our data do not distinguish whether the contribution of the Sla2 coiled-coil-REND-containing region arises from canonical coiled-coil packing, self-association, or other weak multivalent interactions, and the underlying molecular interface remains to be determined.

When analyzing condensate formation in the context of lipidic membranes, both the ENTH domain in Ent5 and the ANTH domain in Sla2 synergistically facilitated LLPS. Additionally, we observed that phosphoinositides (PIPs) help concentrate adaptor proteins on membranes, thereby promoting the nucleation of protein condensates. This protein clustering could be beneficial in facilitating trafficking processes, as it may locally increase adaptor density at the membrane and favor efficient cargo recruitment. Ent5 and Sla2 are recruited to signaling-lipid-containing GUVs and form membrane-associated protein-enriched assemblies under crowding conditions. Because the GUVs were imaged as freely suspended vesicles in single optical planes, these data do not resolve the three-dimensional membrane topology or bilayer number of the observed structures. Thus, while the images support membrane recruitment and membrane-associated assembly, they do not distinguish between membrane folding, vesicle apposition, multilamellar stacking, or other forms of membrane remodeling.

Our results establish a direct link between the structural determinants of LLPS in Ent5 and the regulation of Gga2-dependent coat dynamics. Deleting the dynamic helix of Ent5 impaired its ability to form condensates on membranes in vitro and altered adaptor-mediated trafficking in vivo. Using total lifetime as a readout, we observed that Ent5 Δ249-287 prolonged trafficking events and shifted the population from predominantly short-lived to longer-lived tracks. Importantly, this change in lifetimes occurred without detectable alterations in the recruitment sequence of endocytic proteins, suggesting that condensate dynamics influence the timing of adaptor turnover rather than the order of recruitment. These data provide direct evidence that the structural determinants of LLPS uncovered in vitro also modulate trafficking dynamics in living cells. Consistent with this, Hung and Duncan^27^ reported that disrupting Ent5’s clathrin-binding function impairs coat maturation and prolongs the lifetime of clathrin-coated structures. In their study, mutation of the clathrin-box motifs or complete deletion of *ENT5* led to significantly extended lifetimes of Gga2-marked endosomal/TGN coats, indicating delayed coat disassembly. Our Ent5 Δ249-287 mutant produces a similar phenotype: endocytic trafficking events persist longer than normal (shifting from mostly short-lived to predominantly longer-lived tracks) despite an unaltered recruitment sequence. This parallel suggests that both the clathrin-binding motifs and the phase-separating 249-287 helix of Ent5 are critical for timely coat turnover,

Since clathrin-adaptor interactions are inherently weak in binding affinity^15,36–38^, condensation could contribute to selective recruitment. Not all proteins can partition into a given condensate. Instead, phase-separated condensates concentrate specific client molecules while excluding others^39–41^. This selectivity suggests that drivers of condensates act as biochemical filters that preferentially recruit key functional proteins. While our data establish the molecular determinants and biophysical properties that promote condensate formation in vitro, they do not demonstrate that these assemblies occur as phase-separated condensates in vivo. Rather, our findings provide a mechanistic framework for how membrane recruitment and multivalent interactions could promote local protein enrichment and condensate formation during endocytic and trafficking processes.

In summary, our study shows that distinct structural features in clathrin adaptors act as determinants of LLPS, enabling these proteins to function as drivers or clients depending on context. Together with their ability to engage clathrin and membranes, phase separation emerges as a shared strategy by which adaptors regulate endocytosis and trafficking.

## Methods

### Recombinant protein expression

All constructs were expressed in *Escherichia coli* as follows. Wild-type and variant Ent5 and Ent3 fusions (6×His-Sumo3-Ent5-mEGFP, 6×His-Sumo3-Ent3-mCherry, mCherry, plus all Ent5-mEGFP variants) were cloned into pETM-11 (N-terminal 6×His-Sumo3 tag). The N-terminal domain of yeast clathrin heavy chain (NTD-CHC, 1-369) and Ent1-ENTH/CLC were cloned into pETM-30 (N-terminal His-GST tag with TEV site). Full-length Sla2 and variants, Ede1 (366-900), and CHC (1172-1574) were cloned into pETM-11 and co-expressed with pLysS.

Plasmids were transformed into chemically competent *E. coli* Rosetta2 BL21(DE3) (Ent5/Ent3, Ent1, Ent1-ENTH/CLC), BL21(DE3) (NTD-CHC) or BL21(DE3)pLysS (Sla2/Ede1/NTD-CHC). Transformants were selected on LB agar with 30 µg/mL kanamycin (all) and 34 µg/mL chloramphenicol (Rosetta2) at 37 °C overnight. Several colonies were inoculated in an LB starter culture (same antibiotics) at 37 °C, 200 rpm overnight.

The overnight culture was diluted 1:100 into fresh medium (Ent5/Ent3: LB; NTD-CHC: 2×TY [16 g tryptone, 10 g yeast extract, 5 g NaCl per L]; Sla2/Ede1/CHC distal leg (residues 1172-1572): Terrific Broth) and grown at 37 °C, 200 rpm until OD₆₀₀ reached 0.8–1.0 (for Sla2/Ede1/CHC: 0.6–0.8). Cultures were then cooled: to 18 °C for Ent5/Ent3, ScChc-NTD and Ent1/CLC; to 16 °C for Sla2/Ede1/CHC. Protein expression was induced with IPTG (0.3 mM for NTD-CHC; 1 mM for all others) and allowed to proceed 14–18 h at the induction temperature with shaking.

Cells were harvested by centrifugation for 15 min at induction temperature, 7000 × *g* for Ent5/Ent3, Sla2/Ede1/CHC; 12,000 × *g* for NTD-CHC, and pellets were stored at −20 °C until purification.

### Recombinant protein purification

Recombinant protein purification was performed at 4 °C unless otherwise noted. Frozen cell pellets were thawed on ice and resuspended in lysis buffer (5–6 mL per gram of pellet; see Supplementary Table S2). The suspension was rotated at 8 °C until all visible clumps had dispersed, then lysed by three to four passes through an Emulsiflex C3 homogenizer (Avestin) at 15 kPsi. The crude lysate was clarified by centrifugation at 43,700 × *g* for 1 h at 4 °C and subsequently filtered through a 0.45 µm membrane.

For His-tagged Ent5, Ent3, mCherry and the N-terminal clathrin heavy chain domain (NTD-CHC), the cleared supernatant was loaded at 2 mL/min onto two serial 5 mL HisTrap HP columns (GE Healthcare) pre-equilibrated in buffer B1 followed by buffer A1 (see Table S2). After washing with 20 column volumes of buffer A1 and 5 CV of 5% buffer B1, bound protein was eluted over a 10 CV linear gradient to 100% buffer B1, and 1.5 mL fractions were collected. Sla2, Ede1, Ent1 and CHC^1172-1574^ constructs were purified by batch binding to gravity-flow Roti®Garose Ni–NTA resin (Carl Roth), which was equilibrated in buffer A2, washed with 10 CV of (90% Buffer A2: 10% Buffer B2), and eluted with 10 CV of buffer B2; 1 mL fractions were collected throughout. Fractions containing target proteins were identified by A₂₈₀ nm absorbance and SDS-PAGE, then pooled for tag removal.

His-tagged samples (Ent5, Ent3, Ent1, mCherry, NTD-CHC, Sla2, Ede1 and CHC^1172-1574^) were dialyzed overnight at 4 °C against Buffer 1 (for Ent5, Ent3, Ent1, mCherry and NTD-CHC) or Buffer 2 (for the other constructs) in the presence of SenP2 SUMO protease at 1 mg per 25 mg of fusion protein. NTD-CHC was cleaved instead with TEV protease at the same enzyme: substrate ratio. GST-tagged Ent1-ENTH and CLC were bound to Glutathione Sepharose 4B (Cytiva), washed in buffer A2, and eluted by on-column digestion with 1 mg TEV per mL of resin in buffer A2 overnight at 4 °C.

To remove protease and residual contaminants from Ent5, Ent3 and Ent1, dialyzed samples were adjusted to 0.7 M ammonium sulfate and applied at 4 mL/min to a HiTrap Butyl HP column (GE Healthcare) pre-equilibrated in Milli-Q water, then HIC buffer A. After a 10 CV wash in HIC buffer A, proteins were eluted over an 8 CV gradient to 35% HIC buffer B, held at 35% for 5 CV, and ramped to 100% over 5 CV. One-milliliter fractions were pooled based on SDS–PAGE, concentrated to ~2 mL (Amicon Ultra-15, 10 kDa cutoff), and cleared of precipitate by centrifugation at 21,000 × *g* for 20 min at 4 °C. All other cleaved His-tag constructs (Sla2, Ede1, Ent1-ENTH, CLC, CHC^1172-1574^ and mCherry) were passed three times over gravity-flow Ni-NTA resin equilibrated in their dialysis buffer; the combined flow-through and a 5% imidazole wash were pooled, concentrated, and clarified by centrifugation at 21,000 × *g* for 20 min at 4 °C.

Final polishing was achieved by size-exclusion chromatography. Ent5, Ent3, Ent1 and mCherry were run on a HiLoad 16/600 Superdex 200 pg column equilibrated in SEC Buffer 1. NTD-CHC was purified on a HiLoad 16/600 Superdex 200 pg equilibrated in SEC Buffer 2. ScSla2 full-length, ScSla2-CC, ScSla2-PQcc, and ScEde1-IDR-CC were purified on a Superose 6 Increase 10/300 column, whereas ScSla2-ANTH was purified on a HiLoad 16/600 Superdex 200 pg column. ScSla2-IDR, CLC, and CHC(1172–1574) were purified on a Superdex 200 Increase 10/300 column. Ent1-ENTH was purified on a Superdex 75 10/300 GL column. These proteins were equilibrated in SEC Buffer 3, except where otherwise indicated; the respective protein-specific buffers are listed in Supplementary Table 2. Samples (≤2 mL for 120 mL columns; 0.5 mL for 24 mL columns) were injected and eluted at 0.8 mL/min (120 mL columns) or 0.4 mL/min (24 mL columns), and fractions (120 ml: 1 mL; 24 mL: 0.5 mL) corresponding to the main peak, verified by SDS-PAGE, were pooled, concentrated, and cleared of aggregates by centrifugation at 21,000 × *g* for 20 min at 4 °C. Purified proteins were aliquoted, flash-frozen in liquid nitrogen, and stored at −70 °C.

Representative SDS–PAGE analyzes and size-exclusion chromatography (SEC) profiles of all purified proteins are shown in Supplementary Figs. 13 and 14. Mass spectrometry (MS) peptide fingerprinting was performed to identify degradation products and potential contaminants. MS analyzes were performed by the EMBL Proteomics Core Facility (Heidelberg).

### Construct details and site-directed mutagenesis

Mutations in the plasmids pETM-30-NTD-CHC and pETM-11-Ent5-mEGFP were introduced using a modified QuickChange protocol. Overlapping mutagenic primers (see Supplementary Table S3) were designed to amplify the entire plasmid. Each 50 µL PCR reaction contained 100 ng template DNA, 200 nM of each primer, and 25 µL of 2× Phusion reaction mix [40 mM Tris–HCl, pH 8.8 (25 °C); 4 mM MgCl₂; 120 mM KCl; 20 mM (NH₄)₂SO₄; 0.02 mM EDTA; 0.2% Triton X-100; 8% glycerol; 0.005% Xylene Cyanol FF; 0.05% Orange G; 0.4 mM dNTPs; and 0.04 U/µL Phu-Sso7d polymerase with nuclease-free water]. The PCR cycling conditions were as follows: initial denaturation at 98 °C for 3 min; 20 cycles of 98 °C for 1 min, 56 °C for 45 s, and 72 °C for 7 min; followed by a final extension at 72 °C for 10 min. After amplification, 1 µL of DpnI was added, and the reaction was incubated at 37 °C for 2 h to digest the methylated parental DNA. A 10-µL aliquot was then transformed into chemically competent *E. coli* DH5α cells. The desired mutations were confirmed by Sanger sequencing (Microsynth, Göttingen, Germany). The CAW Clathrin mutant was designed as follows: for the Clathrin box, we introduced mutations K63E, I87D, Q89A and K98E; for the Arrestin box, Q195A, I197T and K251E; and for the W-Box, we mutated residues F26A and Q155A.

### Peptide synthesis

Peptides were purchased from NovoProLabs (Shanghai, China) with at least 98% purity and were TFA-free (acetate salt). The peptides were Ent5 Helix (249-287, ANSNTRRRSHMEEQRRQRREILREQIKNKEQQRKRKQQ) and Ent5-6P (249-287, ANSNTPRRSHMPEQRRPRREPLREQIPNKEQQPKRKQQ). Peptide stocks were solubilized in water at a concentration of 20 mM and then diluted again at a final working concentration in 10 mM Tris, pH 7.5.

### GUV production and imaging

A 20 µL lipid mixture at a final concentration of 1.25 mg/ml was dried on platinum nets. The mixture contained (all molar %) 76% DOPC, 0.1% DOPE-Cy5.5, 16% DOPS, and 8% PI(3,5)P_2_ for GUVs binding Ent5 and 8% PI(4,5)P_2_ for GUVs used in experiments involving Sla2. The GUV lipid composition was chosen to match conditions previously validated for ENTH/ANTH membrane recruitment and remodeling assays^30,31^. The lipid mixture was spread on both nets three times, drying for at least 1 h in a fume hood to remove residual CHCl_3_. A continually heated sucrose solution at >40 °C matching the molality of the SEC Buffer is added to the cuvette to submerge the Pt nets for 30 min. Apply an AC for 2 h, 2.2 V at 10 Hz, then for 20 min apply 2.2 V at 2 Hz.

For imaging, the slide area is incubated with 5% BSA in PBS for 1 h, washed five times with PBS and left soaked in PBS. Add the protein-PEG solution to the slide and subsequently add a one-in-twenty dilution of GUV sucrose solution. Imaging was performed on a Nikon Ti2 spinning disk microscope using the 488 nm and 640 nm excitation wavelengths.

### Mass photometry (MP)

MP experiments were performed using a Refeyn TwoMP mass photometer with AcquireMP software (Version 2024 R1). Protein samples of Ent5WT-mEGFP were adjusted to 1 µM in Experiment Buffer 100 (composition in Supplementary Table S2). The instrument was auto-focused using 18 µL of Experiment Buffer 100 or LLPS Trigger Buffer (see Supplementary Table S2 for composition). Next, 1–2 µL of the protein stock was applied to the measurement chamber and recorded for 1 min at room temperature. Calibration was carried out using the NativeMarker Unstained Protein Standard (Invitrogen, LC0725). Data analysis was performed with DiscoverMP and further processed using custom Python scripts (https://github.com/steniegit/BioPhyPy) based on the eSPC (http://www.spc.embl-hamburg.de) framework^42^.

### LLPS induction and monitoring

Phase separation was induced either by macromolecular crowding or by reduction of ionic strength, depending on the protein construct analyzed. For Sla2 constructs, LLPS was induced by the addition of PEG 8000 as a macromolecular crowding agent. Unless otherwise indicated, samples were supplemented with 5% (w/v) PEG 8000 for Ent5 (when a crowding agent was used) and 2.5% (w/v) PEG 8000 for Sla2 immediately prior to analysis. For Ent5 constructs, LLPS was induced either by macromolecular crowding or by lowering the salt concentration. For low-salt induction, proteins were dialysed overnight into a buffer containing 150 mM Tris (pH 7.5), 100 mM NaCl, and 0.5 mM TCEP (Supplementary Table S2). Immediately before analysis, samples were diluted 1:5 into Tris buffer (pH 7.5, 0.5 mM TCEP), resulting in a final buffer composition of approximately 100 mM Tris, 20 mM NaCl, and 0.5 mM TCEP. For crowding-induced LLPS, PEG 8000 was added to the indicated final concentration. LLPS was monitored by turbidity measurements, microscopy, FRAP, and dynamic light scattering (DLS), as described in the corresponding sections. For turbidity-based phase-separation assays, absorbance/scattering at 600 nm was measured immediately after sample preparation using a CLARIOstar microplate reader (BMG Labtech) in 384-well plates. Buffer controls containing identical concentrations of PEG 8000 and/or salts were measured in parallel and subtracted from sample values. Phase diagrams were generated by quantifying buffer-corrected absorbance at 600 nm as a function of NaCl or PEG 8000 concentration. Protein stability and aggregation during LLPS induction were assessed using a Panta instrument (NanoTemper Technologies). Protein unfolding was monitored by intrinsic fluorescence (nanoDSF),

### Thioflavin T fluorescence and electron microscopy analysis

Thioflavin T (ThT) fluorescence assays were performed in an assay buffer containing 30 mM HEPES (pH 8.0), 150 mM NaCl, and 0.5 mM TCEP. A 1.5 mM ThT stock solution was prepared in assay buffer and filtered through a 0.22 µm syringe filter. ThT was used at final concentrations of 20 µM or 50 µM. PEG was prepared as a 25% (w/v) stock solution and added where indicated. Sla2 IDR-CC-mScarlet or Temporin 1CEa samples were prepared in assay buffer and diluted to 20 μM before the measurement. Reactions were pipetted into a Corning 3881 96-well half-area plate (non-binding surface) with a final volume of 100 µL per well and measured in three replicates. Edge wells were filled with Milli-Q water, and the plate was sealed with RTS thermal sealing film. Fluorescence measurements were performed using a BMG LABTECH CLARIOstar plate reader (Voyager software) at 37 °C in bottom-read mode with excitation at 448 ± 10 nm and emission at 482 ± 10 nm. Measurements were recorded every 10 min for 140 h with automatic gain and without shaking. Fluorescence data were exported and processed using Python. Replicates were averaged.

A representative sample (20 μM Sla2 IDR-CC-mScarlet) was taken from the ThT assay plate after the 140 h incubation and analyzed by negative stain transmission electron microscopy. Carbon-coated grids (CF22H-Cu_TH, EMS) were glow-discharged at 25 mA for 30 s using a gloQube glow-discharge device (QUORUM) in negative charge mode. 8 µL of sample was applied onto the glow-discharged grid and incubated for 30 s before being blotted away. The grid was then washed with approximately 10 µL of Milli-Q water, which was also blotted away. The sample-containing grid was then stained with 2% (w/v) uranyl acetate for 30 s, and excess stain was blotted away. The grid was air-dried on filter paper for 5 min. TEM imaging of the sample grid was performed on a Talos L120c (Thermo Fisher Scientific) with 120 kV accelerating voltage and a LaB6 thermionic source. Micrographs were recorded on a Ceta-M camera (Thermo Fisher Scientific) using a nominal magnification of 57,000×.

### Fluorescence microscopy and FRAP

Images and time-lapse videos of protein droplets were acquired on a Nikon Eclipse Ti2 microscope equipped with a spinning disk confocal unit, an oil-immersion 100× objective (NA 1.49), and an iXon Ultra 888 EMCCD camera. For imaging, mEGFP was excited at 488 nm (1% laser power) and imaged using a 525/5, bandpass emission filter; mCherry was excited at 561 nm (1% laser power), and mScarlet at 561 nm (10% laser power) and a bandpass emission filter 600/25 was used for imaging. For photobleaching (FRAP) experiments, 473 nm and 522 nm lasers were used at 40% power (Rapp OptoElectronic GmbH, Wedel, Germany). Movies were recorded at 500 ms intervals for 90–180 s, with the acquisition starting 10 s before bleaching to establish a baseline.

For quantitative analysis, 99 droplets per condition were examined to determine the partition coefficient (ratio of droplet intensity, *I*_D_, to the surrounding solution intensity, *I*_S_).

Statistical significance was assessed using the two-sided Yuen trimmed-mean tests with Holm–Bonferroni correction. FRAP recovery curves were corrected for photobleaching and normalized using custom Python scripts. The recovery kinetics were fitted to the equation

1 $$I(t)\,=\,A\,(1-{e}^{-\frac{t}{\tau }})+{{{\rm{C}}}}$$

where τ is the recovery time constant, *A* is the mobile fraction, and *C* is the baseline offset. The half-time of recovery (*t*_1/2_) was calculated according to established methods^43^. The fitting parameters for the Ent5 FRAP curves were A = 0.45, tau = 19.2 s and C = 0.43, whereas those for Sla2 were A = 0.47, tau = 112.8 s, C = 0.3.

### Circular dichroism spectroscopy

Peptides corresponding to the WT and 6P mutants were purchased from NovoProLabs (Shanghai, China) at ≥98% purity. Trifluoroacetic acid (TFA) was removed and exchanged with acetic acid before use. CD spectra were recorded on a Chirascan spectrophotometer by averaging three scans at a bandwidth of 1 nm. Measurements were performed in either 1 mm or 5 mm high-precision cuvettes (Hellma Analytics) at a peptide concentration of 20 µM in 10 mM Tris (pH 7.5). The sample temperature was controlled at 20 °C using a TC125 temperature controller (Quantum Northwest, Liberty Lake, USA) unless otherwise stated. Helical content was assessed via a TFE titration series from 0% to 52% (v/v). The free energy of helix formation was determined by fitting the data to a two-state model (Eq. 2) using custom SPC ChiraKit^44^.

2 $$[\varTheta ]=\frac{[{\varTheta }_{222}^{{TFE}}]+[{\varTheta }_{222}^{H2O}]{e}^{(\frac{1}{{RT}}(m\frac{[{TFE}]}{[H2O]}-\Delta {G}_{H2O}))}}{1+{e}^{(\frac{1}{{RT}}(m\frac{[{TFE}]}{[H2O]}-\Delta {G}_{H2O}))}}$$

### Stopped-flow light scattering

Stopped-flow light scattering measurements were performed on a µSFM stopped-flow (Bio-Logic, Grenoble, France) fitted with a MOS-200/M spectrometer. Syringe 1 (100 mM Tris pH 7.5, 0.5 mM TCEP) and syringe 2 (18 mg/mL Ent5-WT in 100 mM Tris pH 7.5, 0.5 mM TCEP, 150 mM NaCl) were rapidly mixed (9:1 buffer:protein, 32 µL total volume) at ~1 mL/s into a µFC-08 cuvette maintained at 8 °C. Measurements were done at 600 nm, detected at 144 V, and sampled at 0.5 ms intervals over 4001 points (0–2 s). Data acquisition began immediately upon mixing. All kinetic traces were fitted by non-linear least squares using two exponential curves using the following equation (Eq. 3):

3 $${{Abs}}_{600{nm}}=B+{A}_{1}{e}^{-t/{\tau }_{1}}+{A}_{2}{e}^{-t/{\tau }_{2}}$$

where *B*, *A*_1_ and *A*_2_ are pre-exponential constants, *t* is the time in milliseconds and ${\tau }_{1}$ and ${\tau }_{2}$ are the two-time constants of the process.

### Stopped-flow time-resolved SAXS

Time-resolved SAXS measurements were performed at the SAXS beamline P12 (EMBL, PETRA III, DESY, Germany) using a stopped-flow mixer (µSFM, BioLogic, Grenoble, France) equipped with a quartz capillary (1.0 mm inner diameter) as a sample delivery system^45^. The device was used to rapidly mix 17.6 mg/ml Ent5-WT in high-salt buffer (100 mM Tris pH 7.5, 0.5 mM TCEP, 150 mM NaCl) with low-salt buffer (100 mM Tris pH 7.5, 0.5 mM TCEP) in a 1:9 ratio with a total volume of 32 µl. Measurements were done at 8 °C, keeping the temperature constant with a circulating water bath (Huber Ministat 240 Thermocycler (Offenburg, Germany)).

SAXS curves were recorded with an Eiger X 4 M detector positioned at a distance of 3 m from the sample using delays ranging from 0 to 380 ms in 20 ms increments, with an exposure period of 20 ms per frame. Two independent series were acquired and averaged for each time point within a *q*-range of 0.02–3.0 nm^−1^. To visualize the time evolution of the reaction, the intensities at *q* = 0.26 nm^−1^ were plotted against the reaction time.

### Protein crystallization and data collection

Purified NTD-CHC concentrated to 10 mg/ml, was mixed with the Ent5.2 peptide (IDDLLDWDGP, NovoProLabs, China) at a final concentration of 8.8 mM, and the mixture was set for co-crystallization using commercially available screens. After 1 day of incubation, a crystal appeared in a condition consisting of 0.056 M sodium phosphate and 1.334 M potassium phosphate. The crystal was retrieved from the droplet and briefly soaked in crystallization buffer saturated with Ent5.2 (CBM2) peptide at 8.8 mM and glycerol as a cryoprotectant at 10% v/v, followed by flash-freezing under liquid nitrogen for storage and X-ray data collection. X-ray diffraction data were collected at a wavelength of 0.9763 Å on beamline P14 at the PETRA III synchrotron, DESY, Hamburg, Germany, at 100 K.

### X-ray diffraction data processing

Raw X-ray diffraction data were indexed and integrated with XDS^46^, identifying C121 space group; the data were then scaled with XSCALE, with data extending to 1.7 Å resolution. Molecular replacement succeeded using the 9EX5 PDB structure as a search model and yielded a solution scoring a TFZ of 76.6 that could place two molecules in the asymmetric unit. The resulting molecular replacement solution displayed a well-defined electron density with clear additional positive density maps tenable for structure modeling. Subsequently, iterative rounds of model building and structure refinement were achieved with Coot^47^ and Refmac^48^. The final refined model contained 777 protein residues, 6471 non-hydrogen atoms, and 391 solvent molecules. The final *R*_work_ and *R*_free_ values were 0.1830 and 0.2114, respectively. Ramachandran analysis showed that 97.63% of residues were in favored regions and 2.37% were in allowed regions, with no Ramachandran outliers.

### Phylogenetic tree reconstruction and AlphaFold3 modeling

Homologs of ScEnt5 (UniProtKB Q03769; strain S288c) were identified in UniRef100 (March 2025) using BLAST+ v2.15^49^ (blastp; *E*-value ≤ 1e-5; soft masking on), then used to build an Ent5-specific profile with MUSCLE v5.1^50^ (seed MSA) and HMMER v3.4 (hmmbuild)^51^, followed by iterative jackhmmer searches against UniRef100 (*N* = 5; incE = 1e-5; domE = 1e-5) to expand the set while limiting drift. To enforce the Ent5-specific ENTH-domain insertion, each retained sequence was modeled with AlphaFold3^52^ and structurally aligned to the yeast Ent5 ENTH reference using TM-align 20160521^53^; reference residues 86-98 were evaluated within a buffered window 80-103, and sequences were kept only if at least half of the reference-mapped columns were non-gaps in the candidate (≥50% occupancy), to check for the insertion specific to Ent5^54^. The final sequence set was aligned with MUSCLE v5.1^50^ (Super5 for large N; trimming of highly gapped/poorly aligned sites prior to inference). Lineage metadata was retrieved from NCBI Taxonomy^55^ (snapshot June 2025) for downstream annotation. Maximum-likelihood trees were inferred with IQ-TREE v2.3.6^56^ using ModelFinder for automatic model selection, and branch support was assessed with 1000 ultrafast bootstrap replicates refined by NNI together with 1000 SH-aLRT replicates. Lineages represented by fewer than three sequences were removed prior to visualization. Trees were rendered in iTOL v7.2.1^57^ (circular layout) and exported as SVG for figure preparation in Inkscape.

### In vivo Gga2-Ent5 recruitment dynamics

*Saccharomyces cerevisiae* cells (MK100 WT: *MATa*; his3Δ200; *leu2-3,112*; *ura3-52*; *lys2-801*) were made competent and transformed as previously described^15^ to tag *GGA2* with mNeonGreen at the C-terminus. Additionally, cells were transformed with pRS315-Ent5-mScarlet (WT or Ent5 Δ249-287; *LEU2* selection marker), and the endogenous *ENT5* was then knocked out (*URA3* cassette). Yeast cells were grown overnight at 30 °C with shaking in a 24-well plate using LD (low-fluorescence SD) Trp^-^, Leu^-^ Ura^-^ medium (yeast nitrogen base without amino acids supplemented with the corresponding dropout mix; Formedium, CYN402, with 2% Glucose). Cells were diluted into fresh medium to OD600 = 0.1 and grown at 30 °C with shaking for 4–6 h to log phase (OD600 = 0.6–1.2). 8-well glass-bottom μ-slides (Ibidi, 80807) were coated with 50 µL Concanavalin A (1 mg/mL; Sigma-Aldrich, C2010; in 10 mM sodium phosphate pH 6, 10 mM CaCl_2_, 1 mM MnCl_2_) for 5 min and washed twice with 50 µL fresh medium. Then 50 µL of cell suspension was applied for 5 min, removed, and wells were washed twice with 50 µL fresh medium to remove unattached cells. Microscopy was performed at room temperature (21  °C) on a Nikon Eclipse Ti2 equipped with 488 nm and 561 nm lasers and dual ORCA-Fusion BT sCMOS cameras in the ALFM Facility (CSSB/DESY, Hamburg) using NIS-Elements v5.42.04. A water-immersion 60× objective (NA 1.27) was used. For each field of view, a 3-min movie was acquired with 100 ms exposure per channel at 2 s intervals (0.5 fps per channel) over 8-11 z-planes (Voxel size: 0.1083 ×0.1083 ×0.7 µm). To compensate for bleaching and/or change of fluorescent signal over time, we merged the two channels as the sum of the signals from both channels. Images were cropped to remove uneven illumination. The cell segmentation and tracking were then performed in the created merged channel. The cell segmentation was performed with Cellpose^58^ to provide cell identity to the tracking result. Tracking was done with TrackMate^59^ using the Simple LAP tracker with linking max distance and gap-closing max distance of 1.0 µm and gap-closing max frame of 0. Only tracks from cells expressing Ent5 were kept, as defined by per-cell SNR and intensity thresholds (median SNR₂ ≥ 0.55, fraction of SNR₂ ≥ 0.70 ≥ 0.10, median *f*₂ ≥ 0.40) applied. Tracks touching acquisition boundaries were removed as timings cannot be determined for those. Each track was background-subtracted using fixed channel-specific values (Gga2: 100, Ent5: 100), then min–max normalized within track per channel. Track durations were binned into six intervals (0–10, 10–15, 16–20, 21–25, 26–30, >30 s), and a χ² test of independence was performed on the resulting contingency table (χ² = 54.43, df = 5, *p* = 1.71 × 10⁻¹⁰). For intensity overlays (Fig. 7d, e), an additional dynamic range filter was applied to exclude low-amplitude traces (defined as normalized range <0.08) prior to alignment. Gga2 traces were aligned to their own intensity peak (*t* = 0), while Ent5 traces were aligned to the Gga2 peak from the corresponding track. Final overlays represent LOESS-smoothed dataset means (span = 0.2), with shaded bands indicating 95% confidence intervals estimated by per-timepoint bootstrapping. Sample sizes post-filtering: WT, *n* = 639; Ent5 Δ249-287, *n* = 525.

### Statistics and reproducibility

Statistical analyzes and data processing were performed using the workflows described in the corresponding Methods sections and figure legends. All statistical tests were performed on the experimental units indicated in the figure legends. For fluorescence microscopy partitioning assays, individual droplets were used as the unit of analysis and partitioning was quantified as the ratio between the mean fluorescence intensity in the dense phase or droplet and that in the surrounding dilute phase or solvent, expressed as *I*_*D*/_*I*_*S*_.

FRAP recovery curves were corrected for photobleaching, normalized, and fitted to a single-exponential recovery model. For Ent5 and Sla2 FRAP analyzes, *n* = 45 and *n* = 24 droplets were analyzed, respectively. Ent5 recruitment assays in Fig. 4 were quantified from n = 99 droplets collected from three independent experiments. Sla2 recruitment assays in Fig. 5 were quantified from *n* = 30 droplets per condition. Ent3 and mCherry partitioning into Ent5 droplets in Supplementary Fig. 11 were quantified from *n* = 50 and *n* = 70 droplets, respectively. For droplet partitioning analyzes, statistical comparisons were performed using two-sided Yuen trimmed-mean tests with Holm–Bonferroni correction for multiple comparisons, and adjusted *P* values are reported in the figure panels.

Turbidity-based phase-separation assays were corrected by subtracting buffer-only controls. The initial absorbance/scattering screen for Ent1, Ent3, and Ent5 was performed with *n* = 3 technical replicates and was used as a screening assay rather than for inferential statistics. Thermal stability and static light-scattering measurements and Ent5 phase diagrams are shown as the mean of three independent experiments. Thioflavin T fluorescence measurements were performed in three replicates, averaged across replicates, and displayed with standard deviations. CD spectra were generated by averaging three scans per condition. Mass photometry measurements were performed in two independent experiments per condition and are presented descriptively; no inferential statistical test was applied to these datasets. Time-resolved SAXS measurements were acquired as two independent series and averaged for each time point.

For in vivo Gga2–Ent5 recruitment dynamics, individual tracked puncta were used as the unit of analysis. Tracks were retained only from cells expressing Ent5 according to defined per-cell signal-to-noise and intensity thresholds. Tracks touching acquisition boundaries were excluded because their durations could not be determined. For intensity overlays, low-amplitude traces with a normalized range < 0.08 were excluded before alignment. Track durations were binned into six intervals: 0–10, 10–15, 16–20, 21–25, 26–30, and >30 s. Genotype distributions were compared using a χ² test of independence. Final post-filtering sample sizes were *n* = 639 tracks for WT and *n* = 525 tracks for Ent5 Δ249–287. For intensity profiles, Gga2 traces were aligned to their own intensity peak, Ent5 traces were aligned to the corresponding Gga2 peak, and LOESS-smoothed dataset means were plotted with 95% confidence intervals estimated by per-timepoint bootstrapping.

Representative microscopy and biochemical experiments were repeated as stated in the corresponding figure legends. Ent5 complementation microscopy, Sla2 truncation microscopy, and the NTD–CHC recruitment experiment on GUVs were each performed in three independent experiments. Protein purification profiles and SDS–PAGE gels are representative quality-control data for the purified samples used in the assays. Crystallographic data were processed and refined as described in the Methods, with data-collection and refinement statistics reported in Table 2.

No statistical method was used to predetermine sample size. Experiments were not randomized, and investigators were not blinded to condition during acquisition or analysis unless otherwise stated.

## Supplementary information

Supplementary information

Supplementary Data

MS data

Transparent Peer Review file

## Data Availability

The X-ray structure of NTD-CHC in complex with Ent5.2 peptide was deposited to the PDB data bank with the 9S6D code. SAXS data were uploaded to SASDB with code SASDYA2. Microscopy images were uploaded to BioImage Archive with code S-BIAD2383. CD spectra were uploaded to Zenodo with code (10.5281/zenodo.17523881). All source data for main figures is included as a Supplementary Data file.

## Code availability

For the in vivo Gga2–Ent5 recruitment dynamics experiments, the fluorescence microscopy analysis pipeline is available at: https://doi.org/10.5281/zenodo.22638611.
