# Supplementary material for: Clathrin adaptors drive phase separation in endocytosis and trafficking
Source: Commun Biol. 2026 Sep 26;9:1260. doi: 10.1038/s42003-026-11043-9 (PMC13615978; doi:10.1038/s42003-026-11043-9)

Supplementary Material for:

"Clathrin adaptors drive phase separation in endocytosis and trafficking"

George Draper-Barr[1,2†], Janina Schiller[2,†], Katharina Veith[1,2], Stephan Niebling[1,2], David Ruiz-Carrillo[1], Ziqiang Huang[3], Christian Tischer[4], Roland Thuenauer[2,5], Lucas A. Defelipe[1,2*] and Maria García-Alai[1,2*]

Affiliations

1- European Molecular Biology Laboratory, Hamburg, 22607, Germany.

2- Centre for Structural Systems Biology (CSSB), Deutsches Elektronen-Synchrotron DESY, Notkestraße 85, 22607 Hamburg, Germany.

3- EMBL Imaging Centre, European Molecular Biology Laboratory, Meyerhofstraße 1, Heidelberg, Germany

4- Data Science Centre, European Molecular Biology Laboratory, Meyerhofstraße 1, Heidelberg, Germany.

5- Technology Platform Light Microscopy (TPLM), Universität Hamburg (UHH), Hamburg, Germany.

† - These authors contributed equally

* - Corresponding authors: Maria García-Alai (maria.garcia@embl-hamburg.de) and Lucas A. Defelipe (lucas.defelipe@embl-hamburg.de).

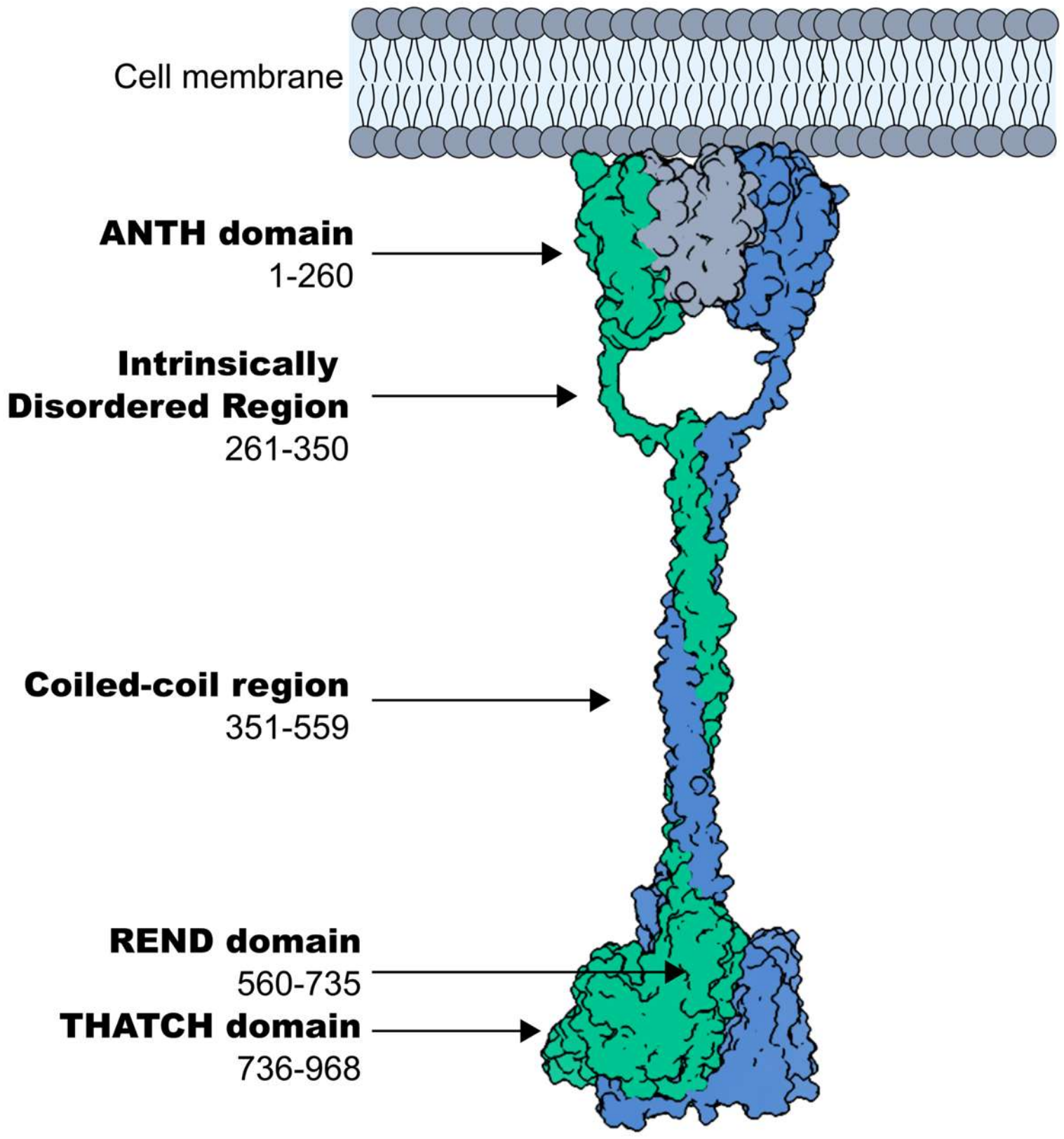

Supplementary Figure 1: Sla2 structural representation scheme (adapted from [1]). Sla2 dimerizes mainly through its coiled-coil region. In this representation, Sla2 monomers are shown in green and blue. Each Sla2 monomer contains an N-terminal ANTH membrane-binding domain (residues 1–260), which binds, in the presence of phosphatidylinositol 4,5-bisphosphate (PIP_2), to the ENTH domain of Ent1 (shown in grey). Two molecules of ANTH and two molecules of ENTH domains form a heterotetrameric membrane complex (the AENTH complex) [2]. The ANTH domains of Sla2 are followed by an intrinsically disordered region (IDR; residues 261–350), a coiled-coil region (residues 351–559), a force-sensing domain (REND; residues 560–735), and an actin-binding domain (THATCH; residues 736–968).

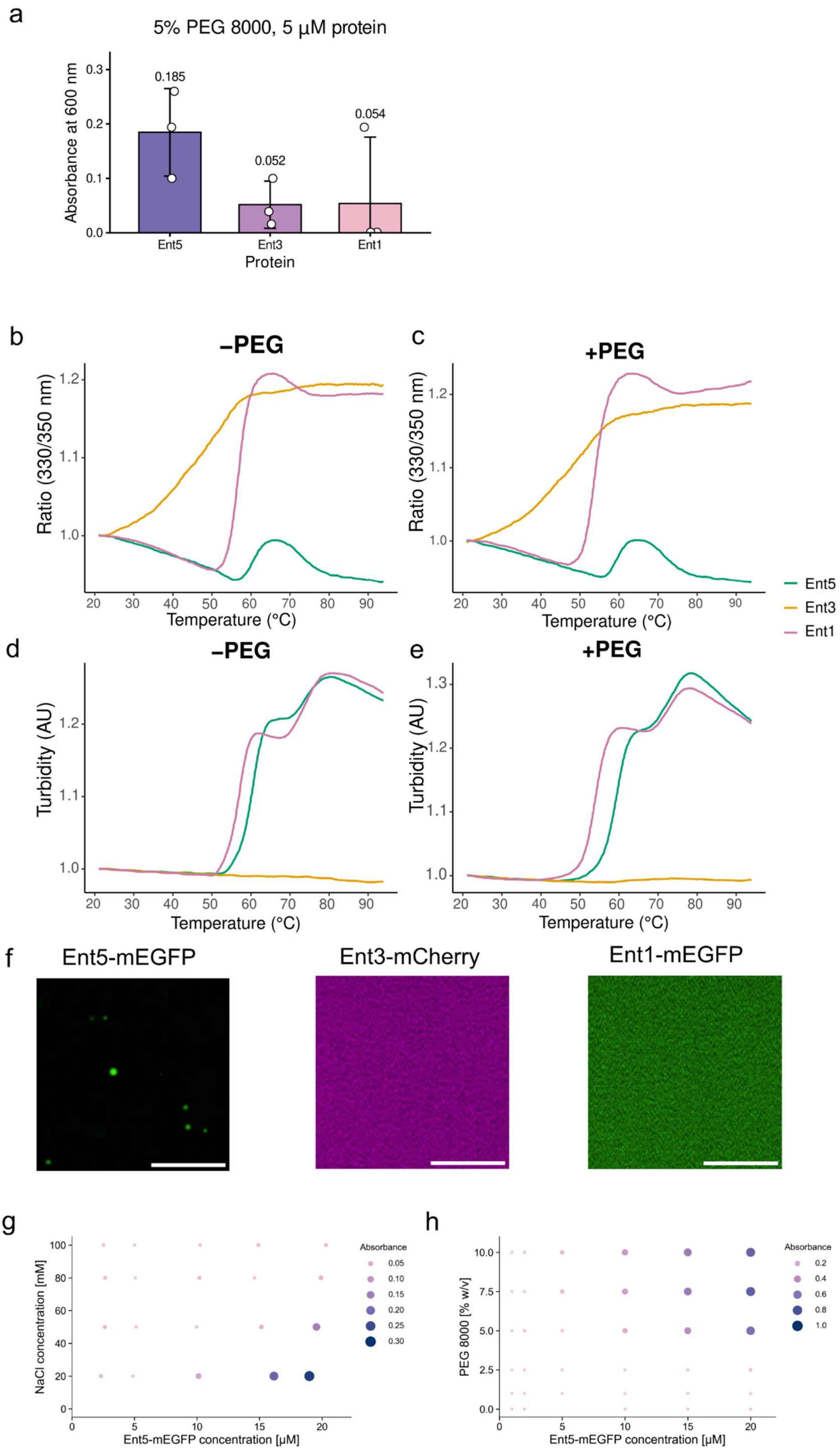
a
5% PEG 8000, 5 µM protein
Absorbance at 600 nm
0.0
0.1
0.2
0.3
0.185
0.052
0.054
Ent5
Ent3
Ent1
Protein
b
–PEG
Ratio (330/350 nm)
1.0
1.1
1.2
Temperature (°C)
20 30 40 50 60 70 80 90
c
+PEG
Ratio (330/350 nm)
1.0
1.1
1.2
Temperature (°C)
20 30 40 50 60 70 80 90
Ent5
Ent3
Ent1
d
–PEG
Turbidity (AU)
1.0
1.1
1.2
Temperature (°C)
20 30 40 50 60 70 80 90
e
+PEG
Turbidity (AU)
1.0
1.1
1.2
1.3
Temperature (°C)
20 30 40 50 60 70 80 90
f
Ent5-mEGFP
Ent3-mCherry
Ent1-mEGFP
g
NaCl concentration [mM]
0
20
40
60
80
100
Ent5-mEGFP concentration [µM]
0 5 10 15 20
Absorbance
0.05
0.10
0.15
0.20
0.25
0.30
h
PEG 8000 [% w/v]
0.0
2.5
5.0
7.5
10.0
Ent5-mEGFP concentration [µM]
0 5 10 15 20
Absorbance
0.2
0.4
0.6
0.8
1.0

Supplementary Figure 2. Ent5 undergoes PEG-dependent phase separation under conditions in which Ent1 and Ent3 remain soluble.

(a) Samples containing 5 µM Ent1-mEGFP, Ent3-mCherry, or Ent5-mEGFP in the presence of 5% (w/v) PEG 8000 were analysed by absorbance/scattering at 600 nm immediately after mixing using a CLARIOstar plate reader in a 384-well plate. Values were corrected by subtracting the corresponding PEG-containing buffer control. Bars show the mean buffer-corrected absorbance, and error bars indicate the SD of technical replicates (n = 3). (b–e) Thermal stability and static light-scattering analysis of Ent1-mEGFP, Ent3-mCherry, and Ent5-mEGFP in the absence or presence of PEG 8000. Protein unfolding was monitored by the intrinsic fluorescence ratio at 330/350 nm (b,c), while aggregation was assessed simultaneously by static light scattering (d,e) using a Panta instrument (NanoTemper). Measurements were performed in 100 mM Tris-HCl (pH 7.5), 150 mM NaCl, and 0.5 mM TCEP, either without PEG (b,d) or with 5% (w/v) PEG 8000 (c,e). Curves show the mean of three independent experiments. Apparent melting temperatures (T_m) were 55.97 ± 0.06 °C and 53.27 ± 0.25 °C for Ent1 in the absence and presence of PEG, respectively; 57.03 ± 0.12 °C and 55.67 ± 0.75 °C for Ent3; and 60.77 ± 0.06 °C and 59.73 ± 0.06 °C for Ent5. Apparent aggregation temperatures (T_m) were 50.06 ± 0.07 °C and 47.71 ± 0.08 °C for Ent1 in the absence and presence of PEG, respectively, and 52.54 ± 0.16 °C and 51.45 ± 0.12 °C for Ent5. Ent3 has no clear transition in the turbidity signal. (f) Representative spinning-disk confocal images of Ent5-mEGFP, Ent3-mCherry, and Ent1-mEGFP under the same PEG-induced LLPS conditions and incubation time used in panel (a). Samples contained 5 µM protein and 5% (w/v) PEG 8000 in 100 mM Tris-HCl (pH 7.5), 150 mM NaCl, and 0.5 mM TCEP. Scale bars, 10 µm. (g,h) Phase diagrams of Ent5-mEGFP phase separation as a function of protein concentration and either NaCl concentration (g) or PEG 8000 concentration (h). In (g), the NaCl concentration was varied by dilution. The plotted values represent the corrected final protein and NaCl concentrations after dilution; consequently, the protein concentrations vary slightly across the NaCl series and do not form a uniformly aligned grid. Phase separation was quantified by buffer-corrected absorbance/scattering at 600 nm using a CLARIOstar plate reader in a 384-well plate. Circle size and colour represent the absorbance values. Measurements were performed at 21 °C and show the mean of three independent experiments. In (g), NaCl concentration was varied by dilution. In (h), NaCl concentration was maintained at 150 mM while PEG 8000 concentration was varied. Unless otherwise indicated, samples contained 100 mM Tris-HCl (pH 7.5) and 0.5 mM TCEP.

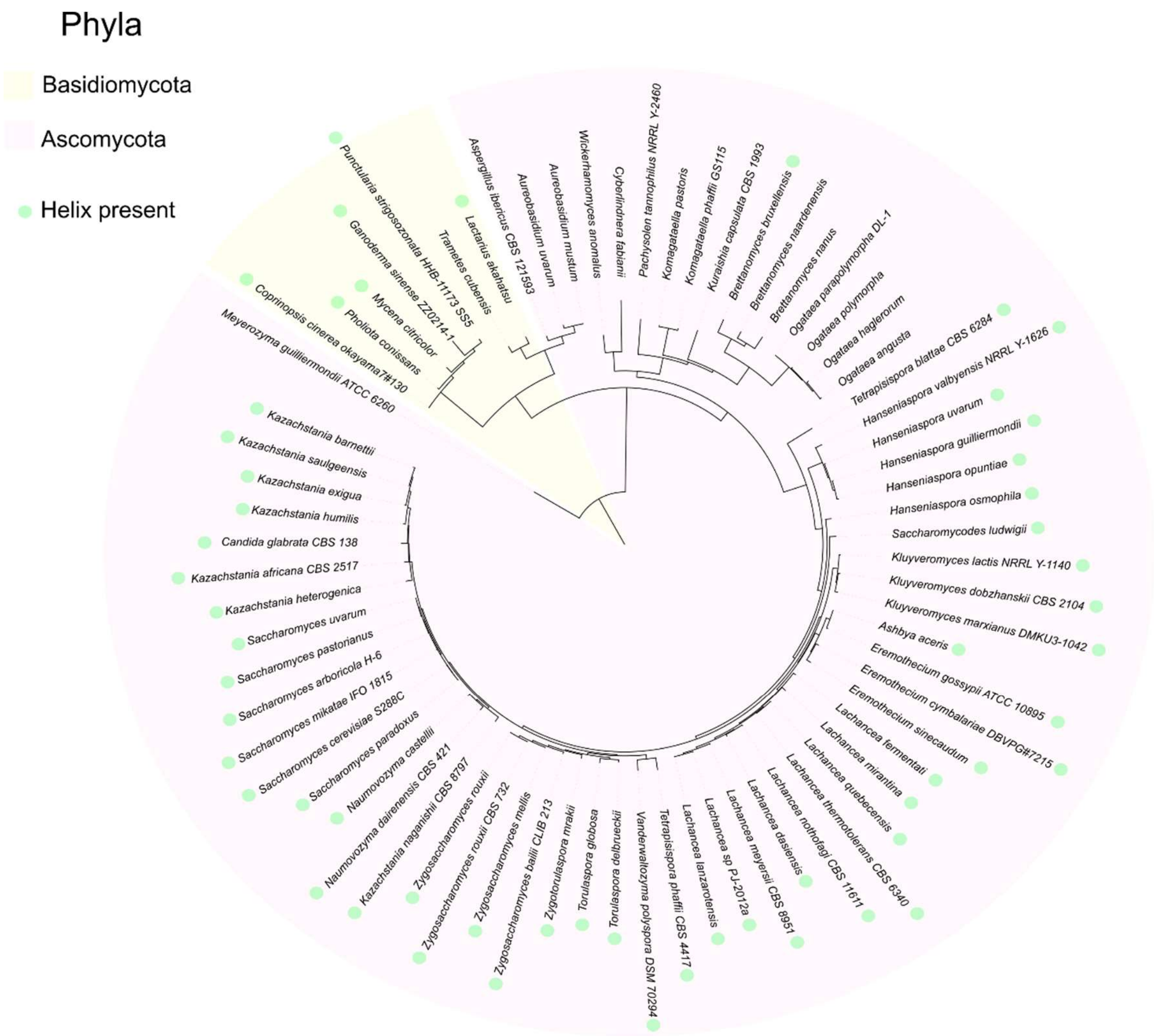

Supplementary Figure 3. Distribution of Ent5 proteins and associated helix across Dikaria. Green dots indicate species in which both Ent5 and the characteristic helix are detected by AF3.

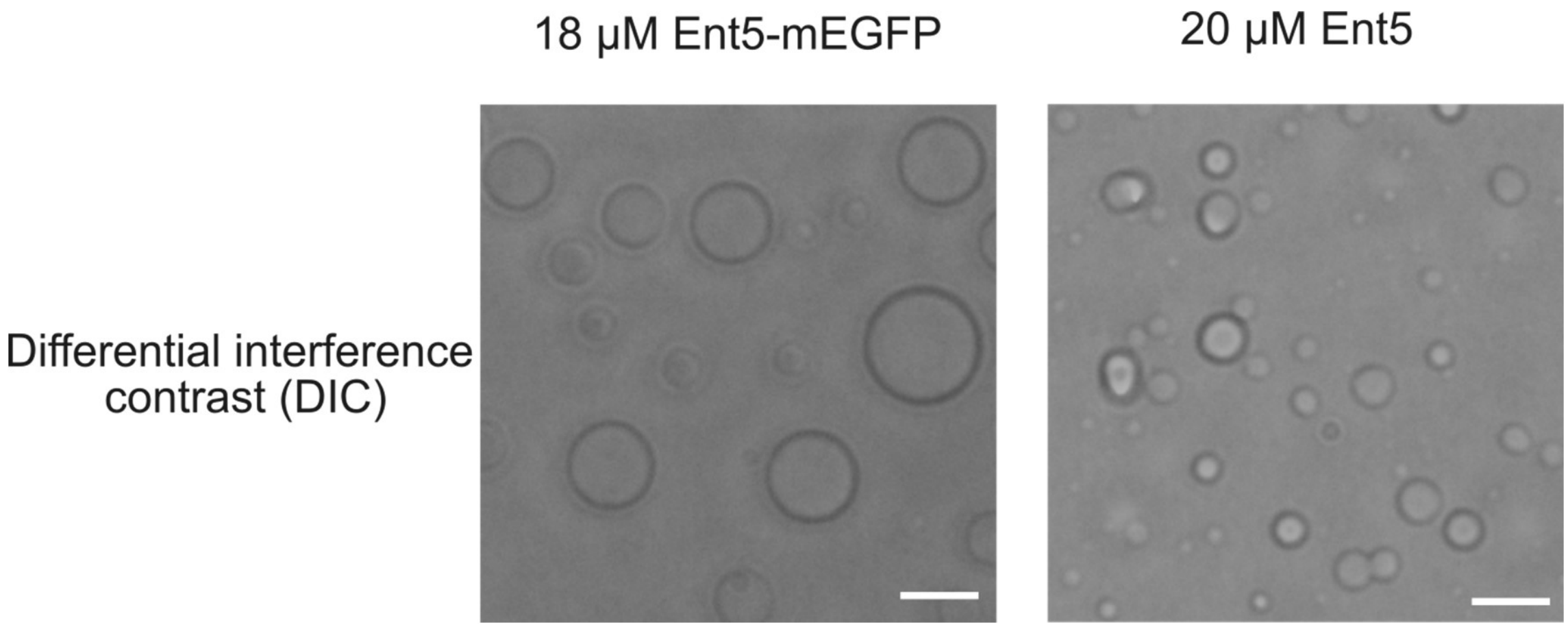

Supplementary Figure 4. DIC imaging of Ent5-mEGFP and unlabeled Ent5. DIC images of Ent5-mEGFP and unlabeled Ent5 demonstrate condensate formation in both samples, confirming that phase separation occurs independent of the mEGFP tag. White scale bars represent 5 µm. Relative to Figure 2.

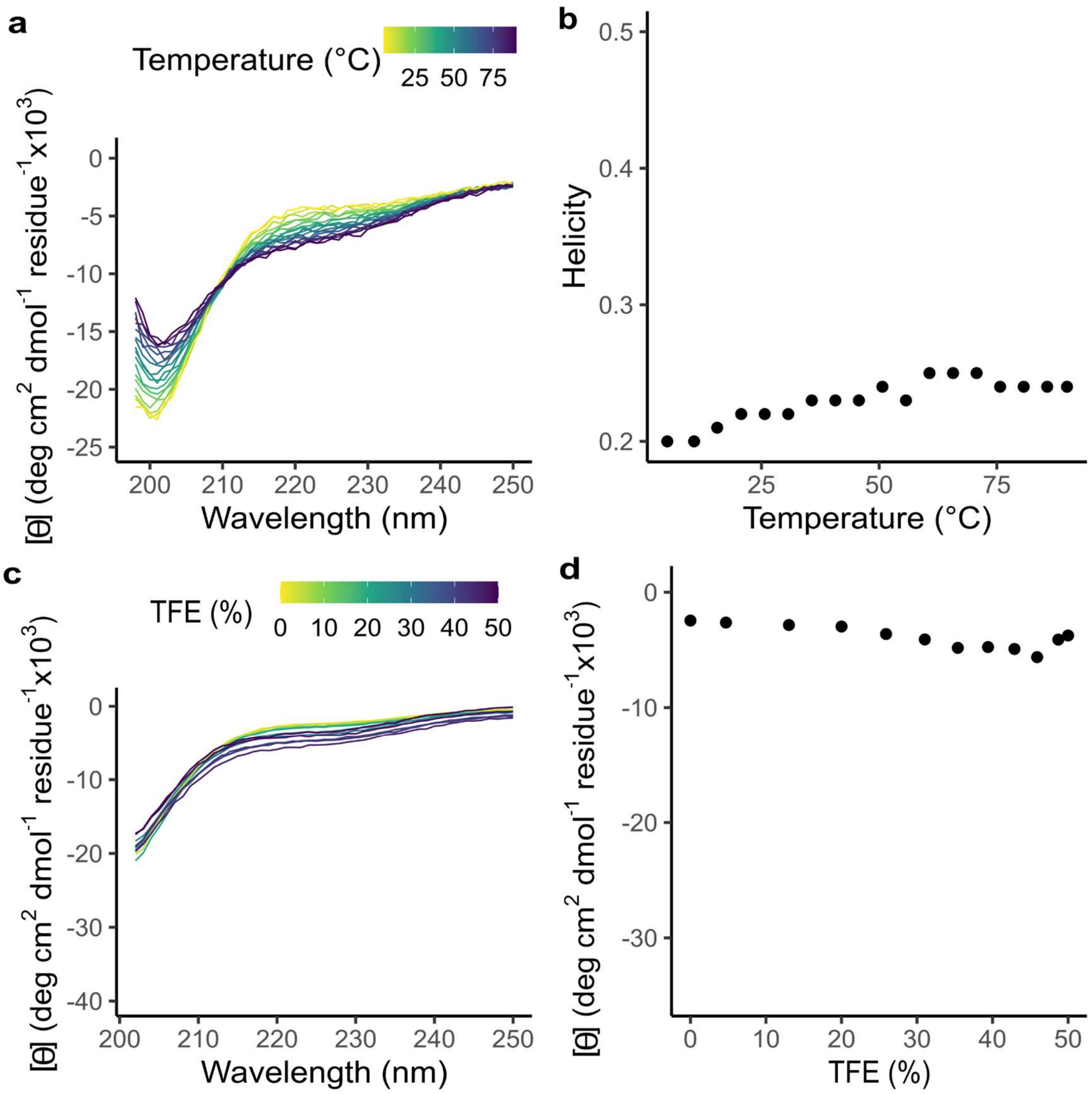

Supplementary Figure 5. Circular dichroism of Ent5 6P peptide. a) Circular Dichroism (CD) spectra of the Ent5 Helix peptide (helix) were recorded over a temperature ramp from 5–90 °C. b)**.** The percentage contribution of helical secondary structure—calculated from the molar ellipticity spectra using the Peptide Helicity module of ChiraKit [3] —is plotted against temperature. c) Far UV CD spectra for the helix were recorded in the presence of TFE titrated from 0 to 50% (v/v) in 10 mM Tris (pH 7.5). d) Molar ellipticity at 222 nm plotted against TFE percentage.

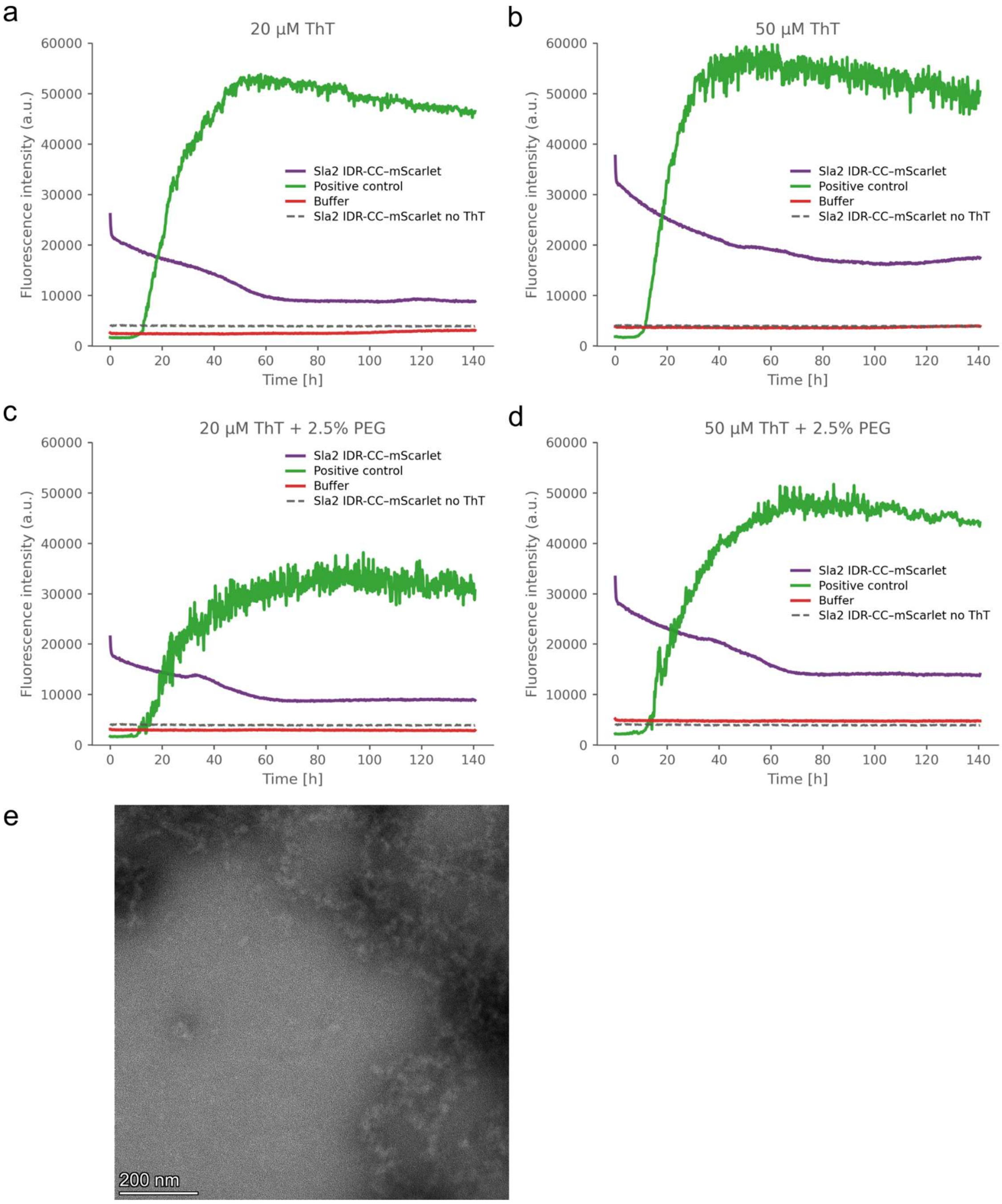

Supplementary Figure 6. Thioflavin T fluorescence and electron microscopy analysis of Sla2 IDR-CC-mScarlet assemblies. Sla2 IDR-CC-mScarlet (violet) and the amyloid-forming positive control Temporin-1CEa (green) were incubated in 30 mM HEPES, pH 8.0, 150 mM NaCl, and 0.5 mM TCEP in the presence of **(a)** 20 µM ThT, **(b)** 50 µM ThT, **(c)** 20 µM ThT plus 2.5% (w/v) PEG 8000, or **(d)** 50 µM ThT plus 2.5% (w/v) PEG 8000. Buffer-only controls (red) and the autofluorescence control of Sla2 IDR-CC-mScarlet in the absence of ThT (grey, dashed) were measured in parallel. Fluorescence was monitored every 10 min for 140 h at 37 °C. Traces represent the mean of three replicates; standard deviations are included but are not visible at this scale. **(e)** Negative-stain electron microscopy micrograph of 20 µM Sla2 IDR-CC-mScarlet after 140 h incubation under the conditions shown in (d). No fibrillar or amyloid-like structures were observed. Scale bar: 200 nm.

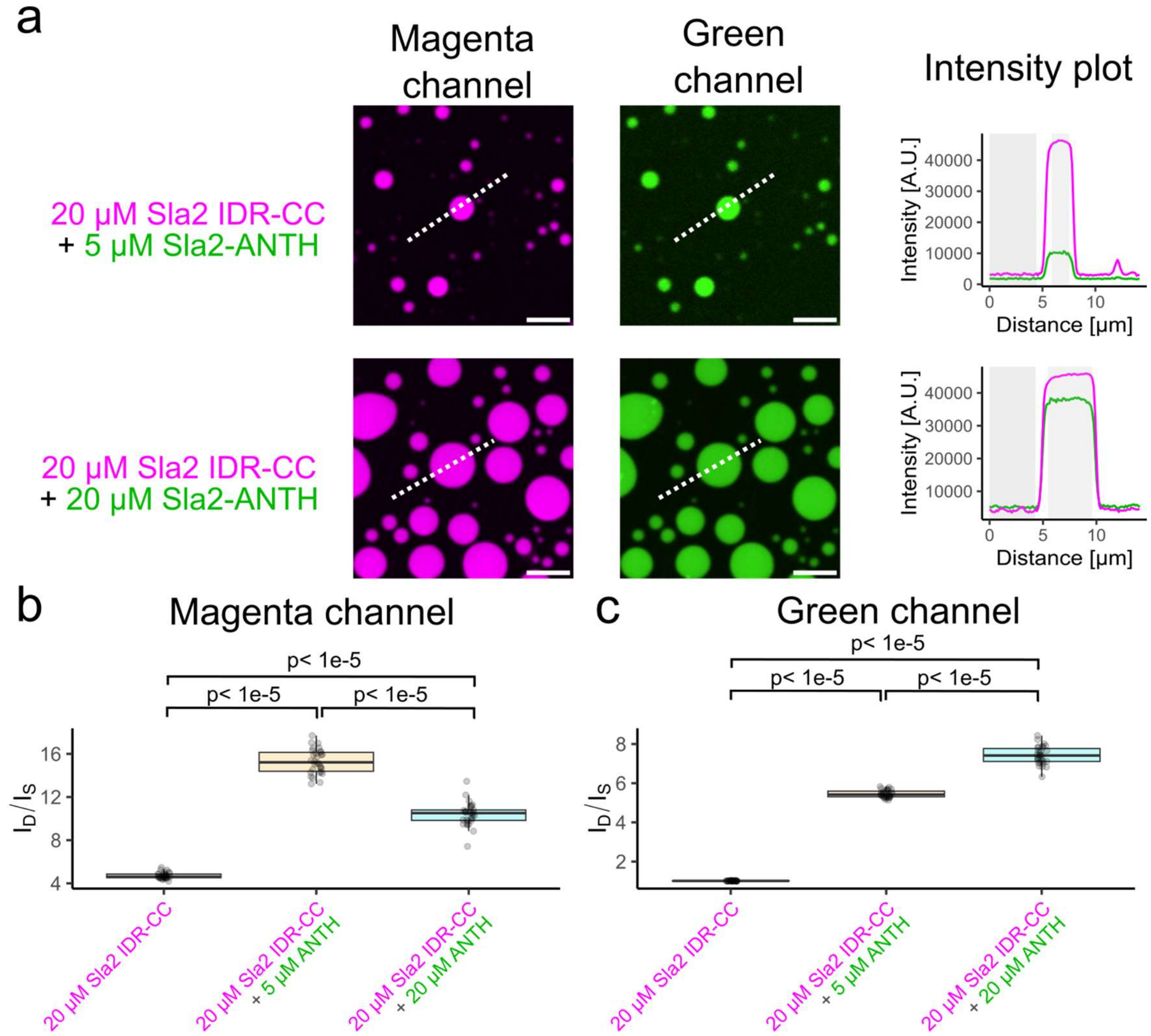

Supplementary Figure 7. Representative imaging and intensity quantification for Sla2 IDR-CC and Sla2 ANTH interactions.

a) Representative condensate images acquired under the same conditions as in Figure 5. Samples contained 20 µM Sla2 IDR-CC–mScarlet combined with either 5 µM or 20 µM Sla2–ANTH–mEGFP. In each image, a central line is drawn to quantify the difference in protein concentration between the droplet (I_D, intensity in the droplet) and the surrounding solvent (I_S, intensity in the solvent). White scale bars represent 5 µm.

b) Average intensity ratios (I_D/I_S) for the magenta channel (Sla2 IDR-CC–mScarlet) are plotted for the two conditions in panel a along with data for Sla2 IDR-CC alone (as reported in Figure 5). c) Average intensity ratios (I_D/I_S) for the green channel (Sla2–ANTH–mEGFP) are plotted for the two conditions in panel a along with data for Sla2 IDR-CC alone (from Figure 5). Statistical analyses were performed as described for Figure 5.

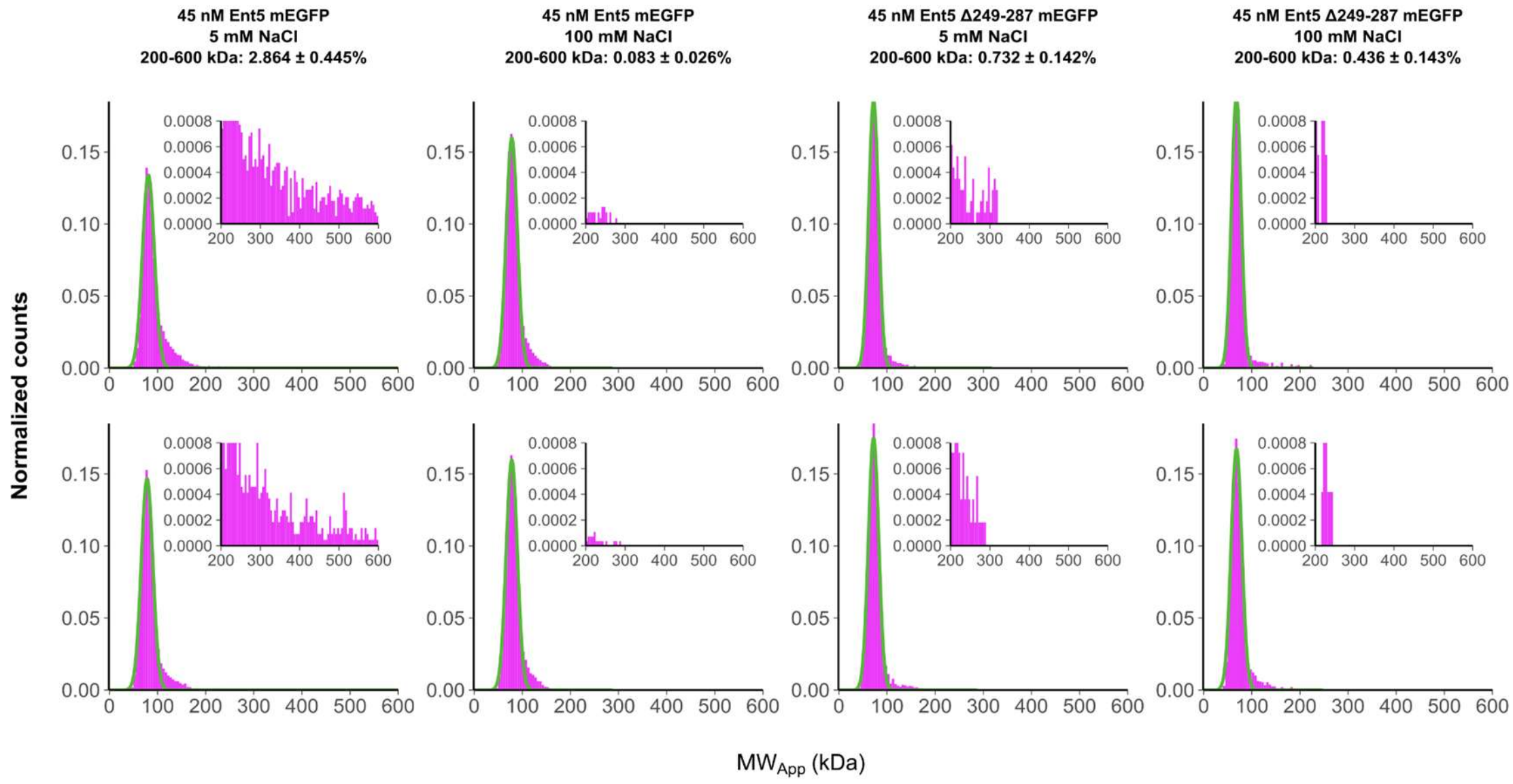

Supplementary Figure 8. Characterization of Ent5-mEGFP nanoclusters by mass photometry. Apparent molecular weight, MW_{app}, histograms obtained from mass photometry measurements of 45 nM Ent5-WT–mEGFP and 45 nM Ent5-Δ249–287–mEGFP in 100 mM Tris, pH 7.5, containing either 5 mM or 100 mM NaCl. Data are shown for n = 2 independent experiments per condition. Normalized histograms are shown in magenta, and fitted Gaussian distributions are shown in green. The expected monomeric molecular masses are 74 kDa for Ent5-WT–mEGFP and 69 kDa for Ent5-Δ249–287–mEGFP. Insets magnify the MW_{app} region between 200 and 600 kDa. Values above each condition indicate the percentage total normalized histogram area within this range, reported as mean ± SD across the two independent experiments. Measurements were performed at room temperature.

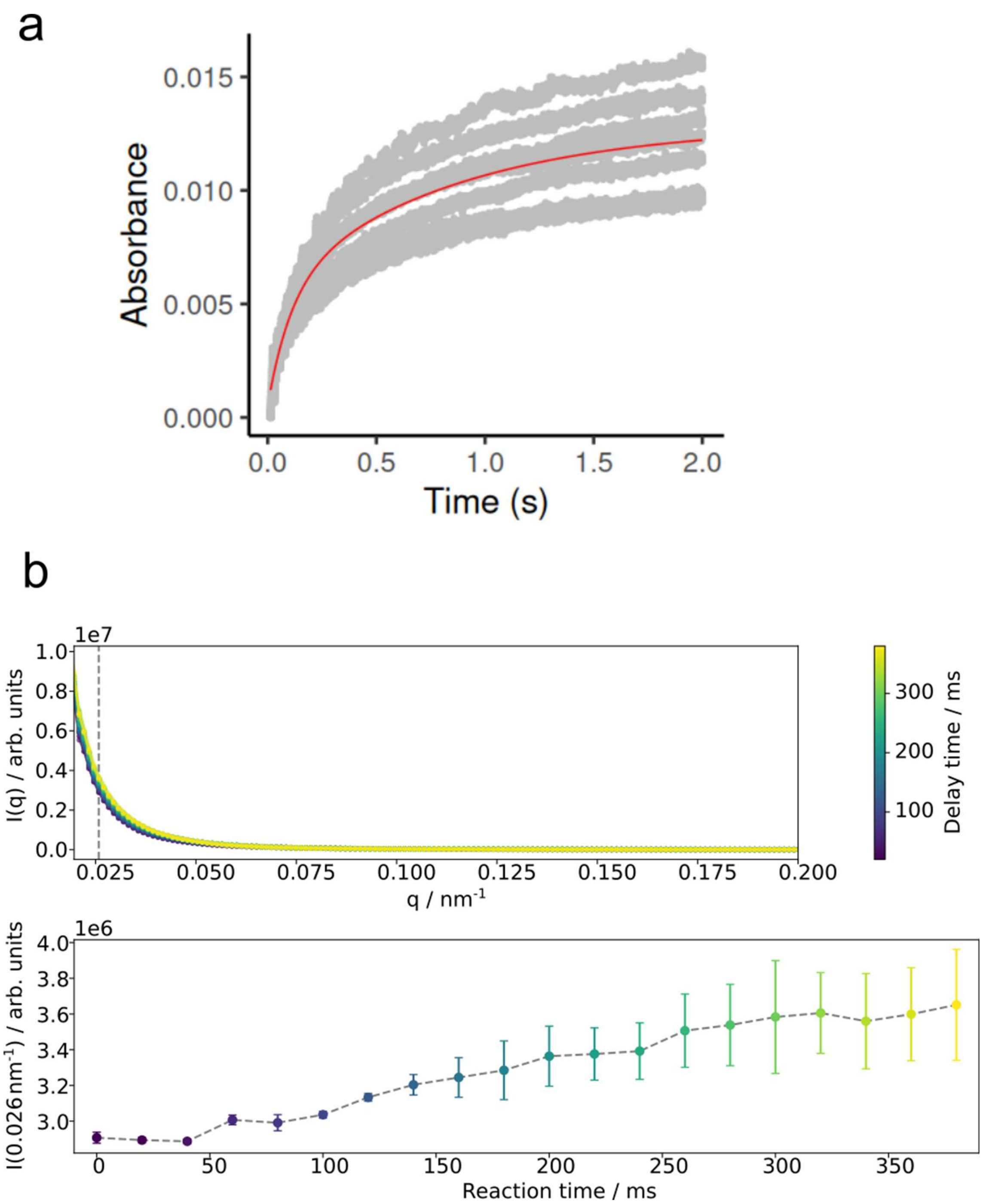

Supplementary Figure 9. Light Scattering and TR-SAXS. a) Stopped-Flow Light Scattering. The red curve shows the fit to a double-exponential curve with time constants of τ_1 (108±7) ms and τ_2 (843±42) ms. b) Time-Resolved SAXS curves for different delay times (top) and a vertical slice at 0.026 nm^{-1} (bottom). Points show the average, and bars show SD.

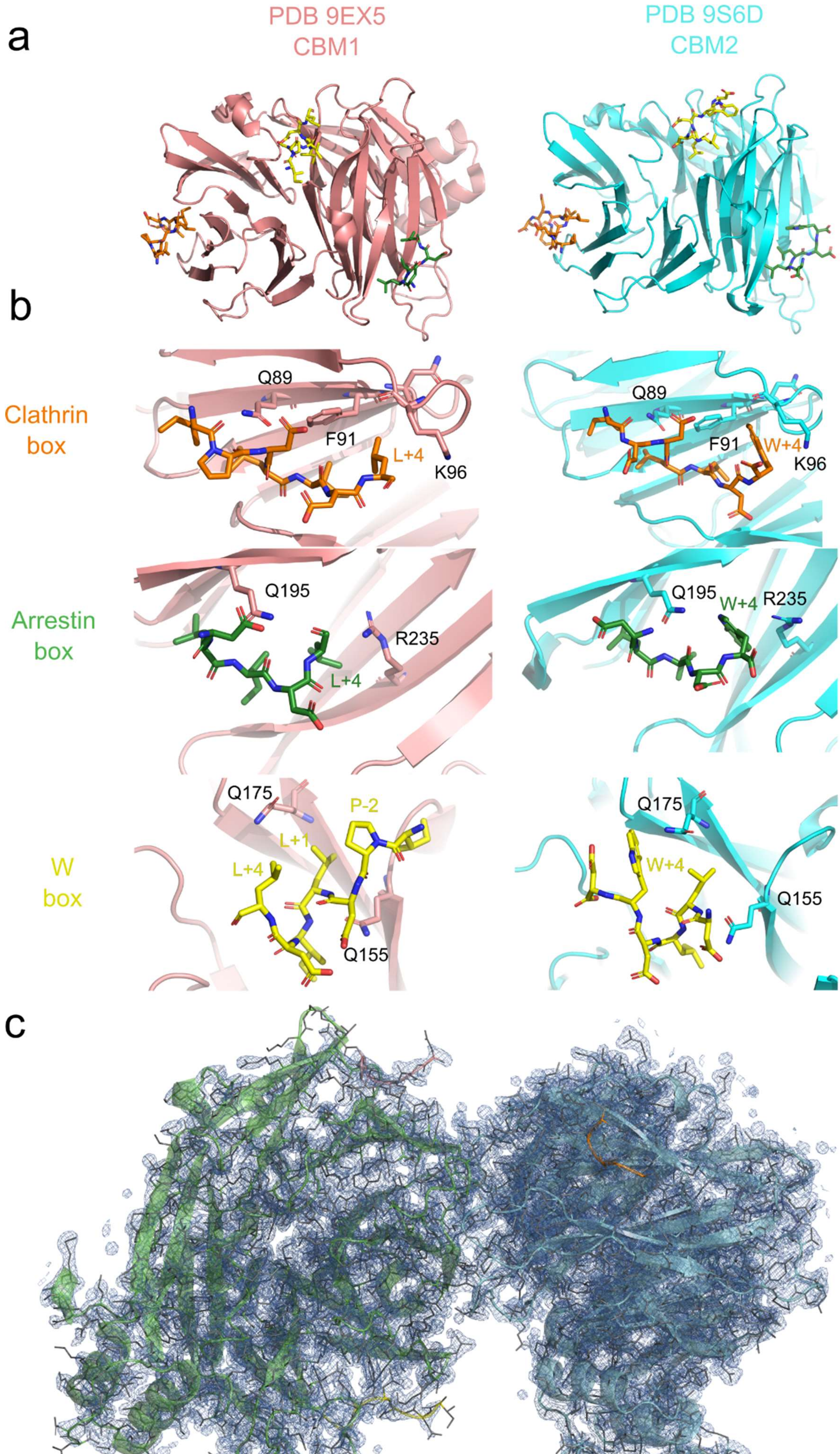
a
PDB 9EX5
CBM1
PDB 9S6D
CBM2
b
Clathrin box
Q89
F91
L+4
K96
Q89
F91
W+4
K96
Arrestin box
Q195
R235
L+4
Q195
W+4
R235
W box
Q175
P-2
L+1
L+4
Q155
Q175
W+4
Q155
c

Supplementary Figure 10. Crystal structure of NTD-CHC in complex with Ent5.2 peptide (LLDW). a) Crystal structure of NTD-CHC bound to CBM1 of Ent5 (Left, PDB 5EX5). Crystal structure of NTD-CHC bound to CBM2 of Ent5 (Right, 9S6D). b) Close-up view of each binding box. c) Whole-structure 2mFo–DFc electron-density map of the NTD–CHC–Ent5.2 peptide complex (PDB 9S6D), shown as a blue mesh surrounding the refined model and contoured at 1.0σ.

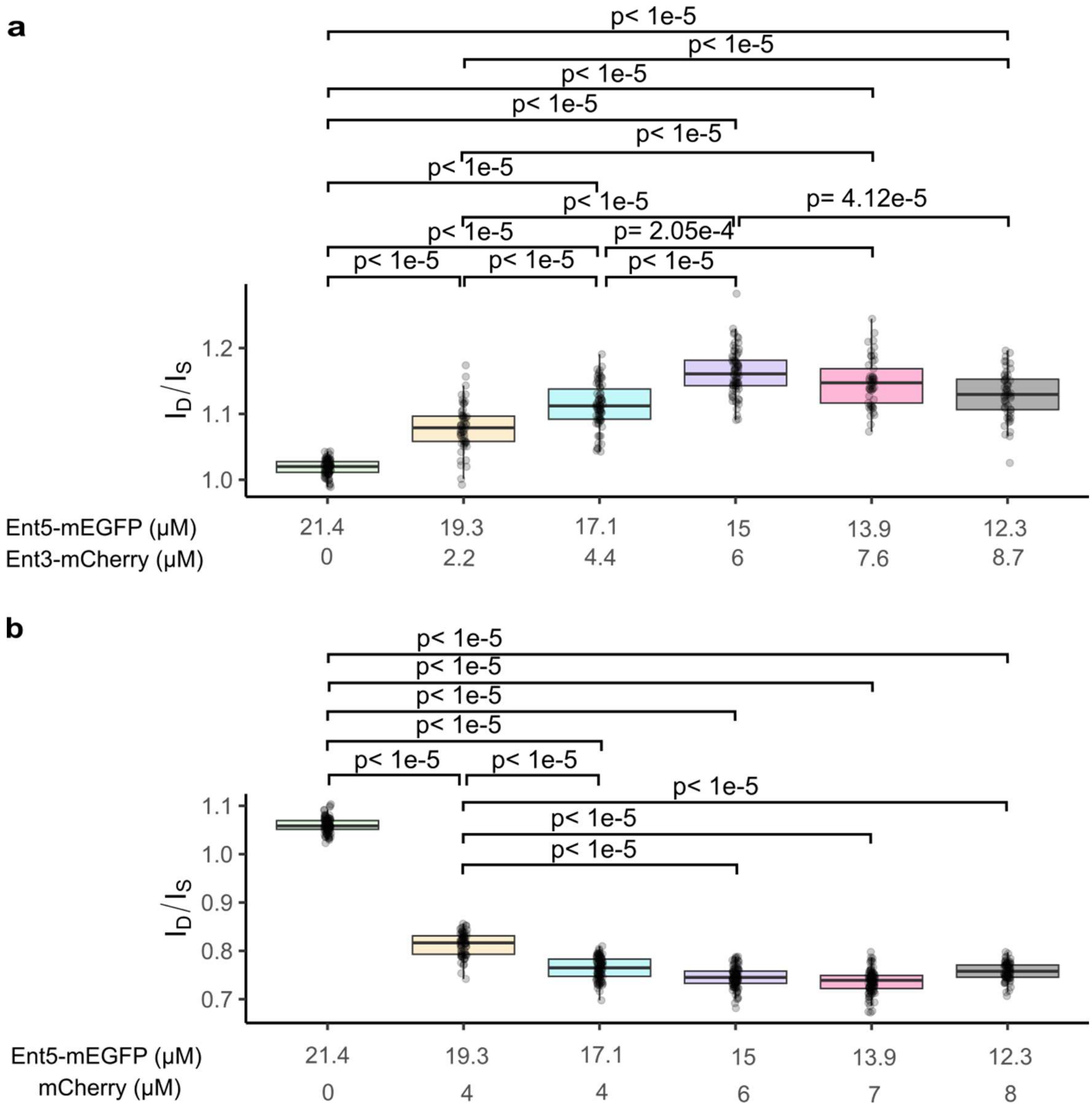

Supplementary Figure 11. Quantitative Analysis of Ent3 Partitioning into Ent5 Droplets. a) Partitioning coefficient of Ent3WT–mCherry into Ent5WT–mEGFP droplets is measured over a concentration range of 0–8.7 μM (Ent3WT–mCherry) and 12.3–20.4 μM (Ent5WT–mEGFP); n = 50 droplets were analyzed. b) Partitioning coefficient of mCherry into Ent5WT–mEGFP droplets is measured over a concentration range of 0–8.0 μM (mCherry) and 12.8–21.4 μM (Ent5WT–mEGFP); n = 70 droplets were analyzed. Statistical analyses were performed as described for Fig 5.

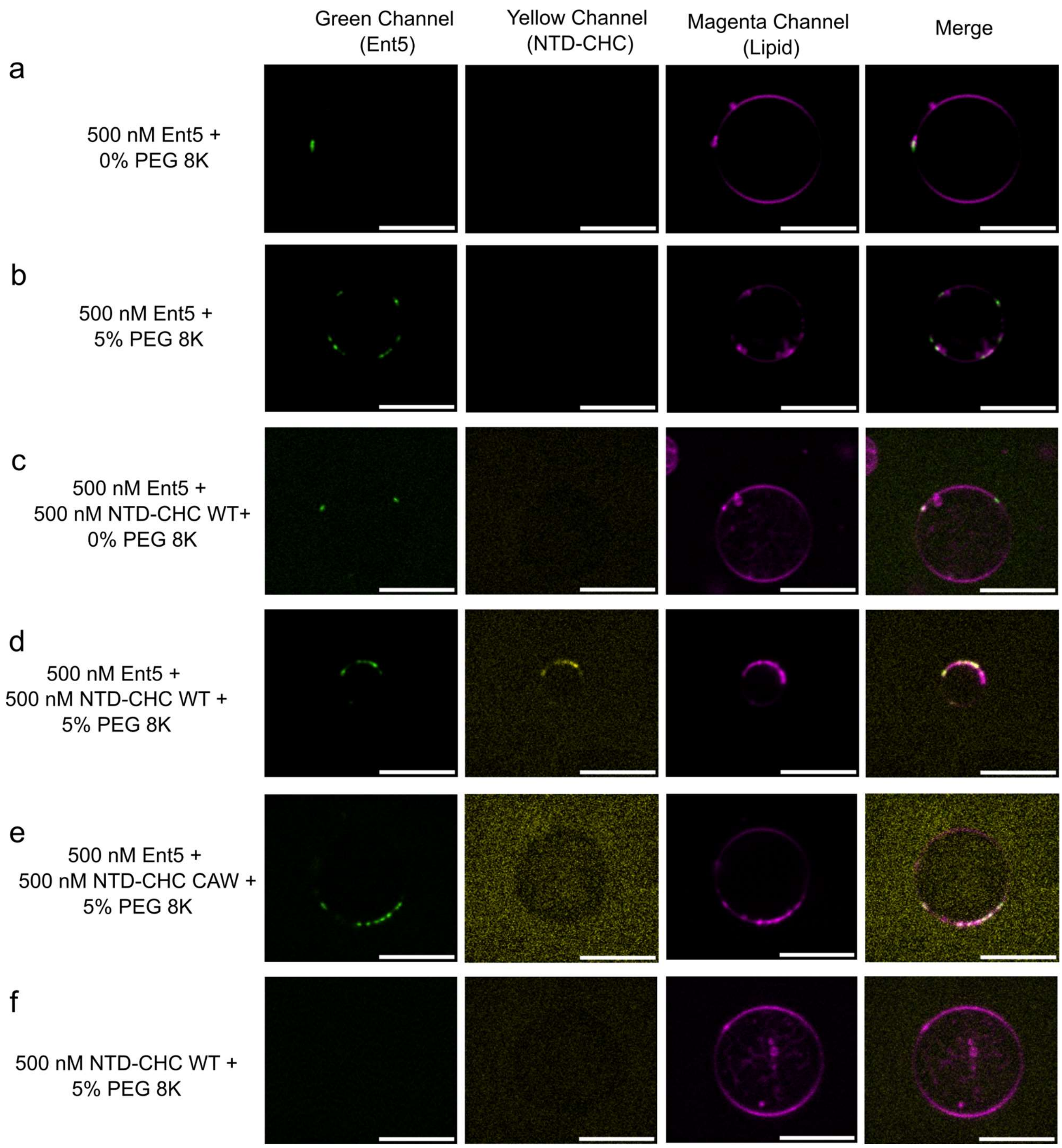

Supplementary Figure 12. NTD–CHC is recruited to Ent5 membrane condensates assembled on PI(3,5)P_2-containing GUVs. Representative spinning-disk confocal images of GUVs in presence of Ent5-mEGFP and NTD-CHC-mScarlet. GUVs were incubated for 5 min at room temperature with Ent5–mEGFP (green) and/or NTD–CHC–mScarlet (yellow) in 100 mM Tris-HCl, pH 7.5, 150 mM NaCl, and 0.5 mM TCEP. The lipid membrane is shown in magenta (DOPE-Cy5.5). (a) 500 nM Ent5–mEGFP without PEG 8000. (b) 500 nM Ent5–mEGFP with 5% (w/v) PEG 8000. (c) 500 nM Ent5–mEGFP and 500 nM WT NTD–CHC–mScarlet without PEG 8000. (d) 500 nM Ent5–mEGFP and 500 nM WT NTD–CHC–mScarlet with 5% (w/v) PEG 8000. (e) 500 nM Ent5–mEGFP and 500 nM NTD–CHC CAW–mScarlet with 5% (w/v) PEG 8000. (f) 500 nM WT NTD–

CHC–mScarlet with 5% (w/v) PEG 8000. Under crowding conditions, WT NTD–CHC is recruited to Ent5-rich membrane condensates, whereas the CAW mutant shows no comparable recruitment. Images are representative of three independent experiments. Scale bars, 10 μm.

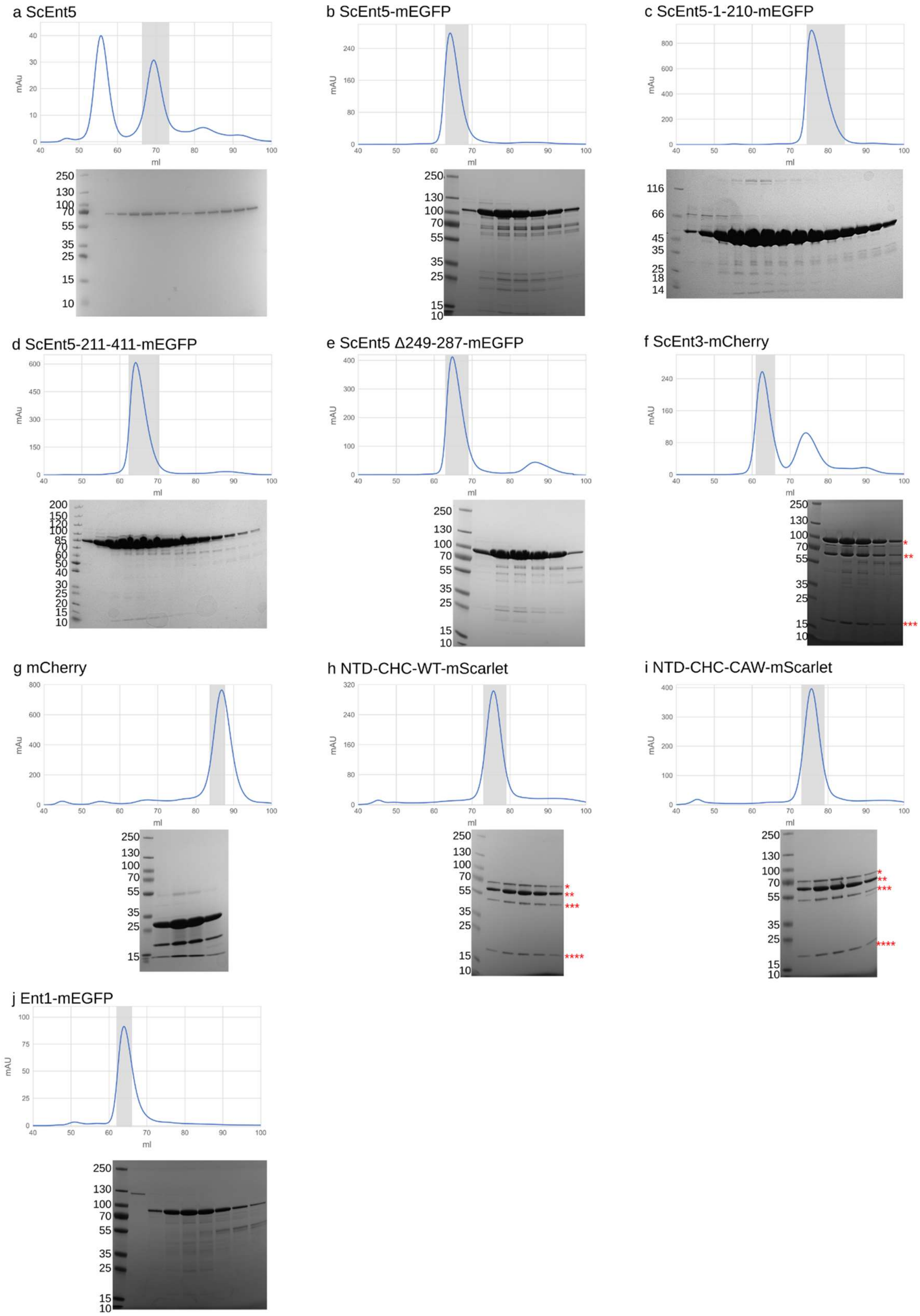

Supplementary Figure 13. Purification of fluorescently labelled epsin constructs, clathrin N-terminal domain constructs, and fluorophore controls by size-exclusion chromatography. Preparative size-exclusion chromatography (SEC) profiles and corresponding SDS–PAGE analysis of pooled fractions. Samples marked with red asterisks were submitted for MS mass fingerprinting (see Supplementary MS

Information). (a) *S. cerevisiae* Ent5 (expected MW = 47.2 kDa), (b) ScEnt5-mEGFP (expected MW = 74.3 kDa), (c) ScEnt5(1–210)-mEGFP (expected MW = 51.6 kDa), (d) ScEnt5(211–411)-mEGFP (expected MW = 50.0 kDa), (e) ScEnt5Δ249–287-mEGFP (expected MW = 69.9 kDa), (f) ScEnt3-mCherry (expected MW = 72.8 kDa; * indicates intact ScEnt3-mCherry, ** a ScEnt3 degradation product, and *** free mCherry), (g) mCherry (expected MW = 26.6 kDa), (h) NTD–CHC-WT-mScarlet (expected MW = 67.5 kDa; * and ** indicate full-length NTD–CHC-WT-mScarlet, *** an NTD–CHC-WT degradation product, and **** free mScarlet), (i) NTD–CHC-CAW-mScarlet (expected MW = 67.2 kDa; * and ** indicate full-length NTD–CHC-CAW-mScarlet, *** an NTD–CHC-containing degradation product, and **** free mScarlet), and (j) Ent1-mEGFP (expected MW = 79.3 kDa). Proteins were purified by SEC using a HiLoad 16/600 Superdex 200 pg column in the respective protein-specific buffers listed in Supplementary Table 2. Grey shaded regions in the chromatograms indicate the fractions selected for pooling. Pooled fractions were analysed by SDS–PAGE using 4–20% gradient gels followed by Coomassie staining. Molecular-mass markers are indicated in kDa.

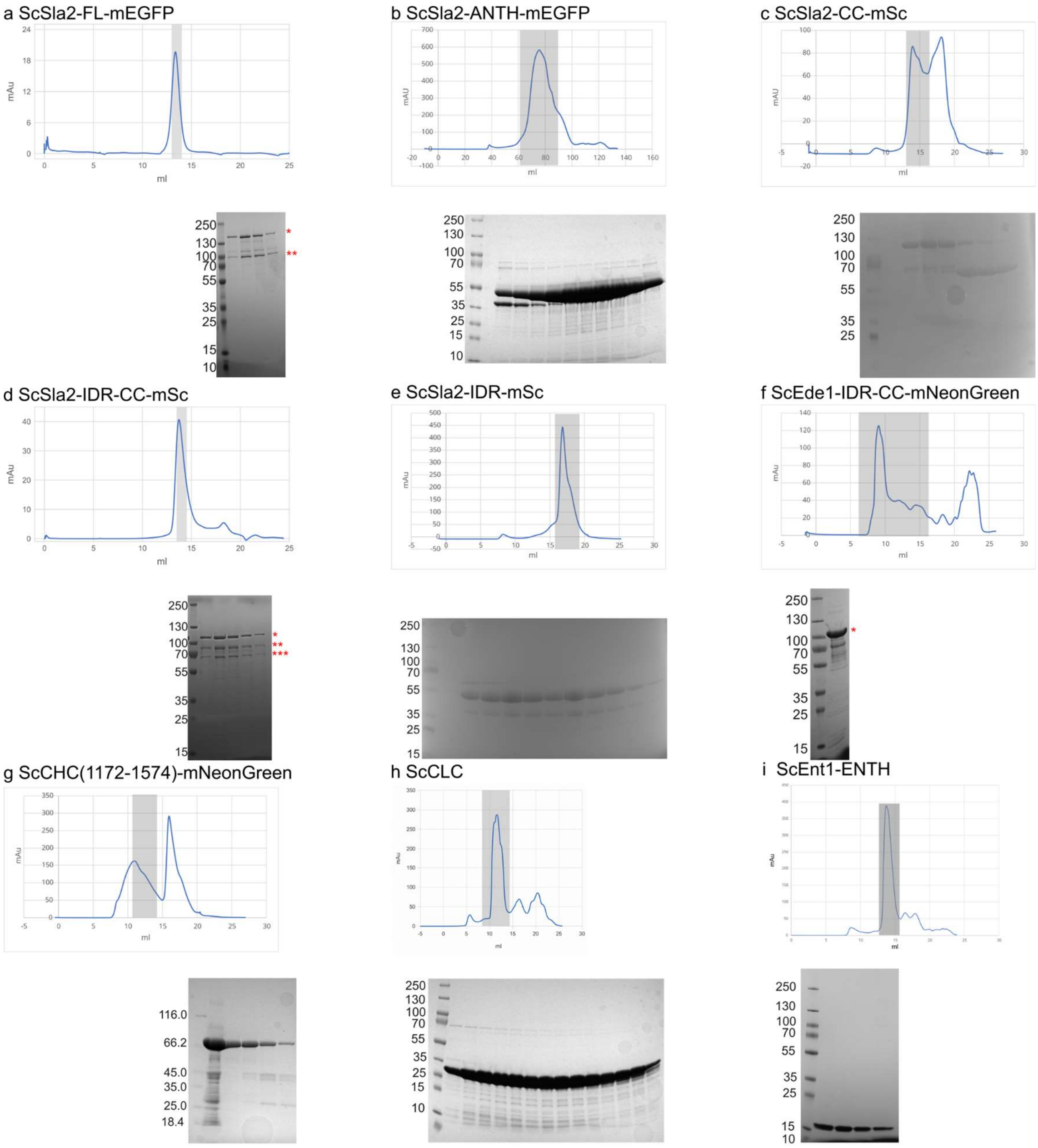

Supplementary Figure 14. Purification of fluorescently labelled Sla2, Ede1, clathrin heavy-chain, and clathrin light-chain constructs by size-exclusion chromatography. Preparative size-exclusion chromatography (SEC) profiles and corresponding SDS–PAGE analysis of pooled fractions. Samples marked with red asterisks were submitted for MS mass fingerprinting (see Supplementary MS Information). (a) *S. cerevisiae* Sla2 full-length-mEGFP (ScSla2-FL-mEGFP; expected MW = 136.6 kDa), purified using a Superose 6 Increase 10/300 column (* indicates full-length ScSla2-FL-mEGFP, ** and *** indicate N-terminally truncated ScSla2 species); (b) ScSla2-ANTH-mEGFP (expected MW = 60.4 kDa), purified using a HiLoad 16/600 Superdex 200 pg column; (c) ScSla2-CC-mScarlet (expected MW = 72.4 kDa), purified using a Superose 6 Increase 10/300 column; (d) ScSla2-IDR-CC-mScarlet (expected MW = 79.7 kDa), purified using a Superose 6 Increase 10/300 column (* and ** indicate intact ScSla2-IDR-CC-mScarlet, *** indicates an ScSla2-IDR-CC-mScarlet degradation product lacking peptides from the IDR region); (e) ScSla2-IDR-mScarlet (expected MW

= 38.0 kDa), purified using a Superdex 200 Increase 10/300 column; (f) ScEde1-IDR-CC-mNeonGreen (expected MW = 86.5 kDa), purified using a Superose 6 Increase 10/300 column (* indicates an N-terminally truncated ScEde1-IDR-CC-mNeonGreen lacking peptides corresponding to approximately the first 60 amino acids); (g) ScCHC(1172–1574)-mNeonGreen (expected MW = 79.4 kDa), purified using a Superdex 200 Increase 10/300 column; (h) ScCLC (expected MW = 26.5 kDa), purified using a Superdex 200 Increase 10/300 column; and (i) ScEnt1-ENTH (expected MW = 18.9 kDa), purified using a Superdex 75 Increase 10/300 column. Proteins were purified in the respective protein-specific buffers listed in Supplementary Table 2. Grey shaded regions in the chromatograms indicate the fractions selected for pooling. Pooled fractions were analysed by SDS–PAGE using 4–20% gradient gels followed by Coomassie staining. Molecular-mass markers are indicated in kDa.

Supplementary Table 1. IDR Regions and HotSpot residues for LLPS.

Name	IDR region	pLLPS	Droplet-promoting regions
Apl2	637-726	0.21	694-721
Apl5	648-931	0.36	721-771;783-875;897-932
CHC	-	0.15	1623-1653
CLC	1-110	0.97	1-28;33-125
Ede1	228-274, 367-597,884-1340	0.99	98-139;203-214;235-250;360-565;571-585;701-713;803-815;879-1029;1032-1054;1058-1147;1152-1202;1255-1286;1295-1340
Ent1	306-454	0.97	108-118; 136-197; 241-226; 253-265; 290-369;425-452
Ent2	416-613	1.00	128-212;227-257;286-415;423-613
Ent3	173-408	0.99	158-272; 285-321; 340-378
Ent5	206-411	0.97	199-411
Gga1	1-18; 317-440	0.31	1-18;371-381;541-557
Gga2	1-24; 335-470	0.73	256-269;335-351;354-465
Sla1	130-253;412-497;619-656;718-1244	1.00	123-254; 397-510; 548-657; 718-858; 1027-1056; 1091-1111; 1129-1140; 1199-1228
Sla2	260-351	0.57	278-370; 385-428; 593-604
Swa2	1-140;250-367;516-551	0.84	1-98;295-363;451-464;513-560
Yap180	324-637	0.97	282-299;333-387;400-414;424-459;470-496;520-

1			542;559-624
Yap180 2	325-568	0.89	255-438;442-454;556-568

Supplementary Table 2. Buffer Composition Table

Buffer name	Composition
PCR buffer	40 mM Tris–HCl, pH 8.8 (25°C)<br>4 mM $MgCl_2$<br>120 mM KCl<br>20 mM $(NH_4)_2SO_4$<br>0.02 mM EDTA;<br>0.2% Triton X-100;<br>8% glycerol;<br>0.005% Xylene Cyanol FF;<br>0.05% Orange G;<br>0.4 mM dNTPs;<br>0.04 U/µL Phu-Sso7d polymerase
Lysis buffer (Ent5, Ent3, Ent1, mCherry)	20 mM Sodium Phosphate NaP pH 7.5<br>200 mM NaCl<br>0.05% Tween-20<br>2 mM MgCl2<br>0.5 mM TCEP<br>400 U DNAse I<br>Complete EDTA-Free protease inhibitor
Lysis Buffer (Sla2, Ede1, CLC, $CHC^{1172-1574}$, Ent1-ENTH)	30 mM HEPES pH 8<br>300 mM NaCl<br>5 % w/v glycerol<br>400 U DNAse I<br>Complete EDTA-Free protease inhibitor
Lysis buffer (NTD-CHC)	20 mM Sodium Phosphate NaP pH 7.5<br>200 mM NaCl<br>0.05% Tween-20<br>2 mM MgCl2<br>0.5 mM TCEP<br>400 U DNAse I<br>Complete EDTA-Free protease inhibitor
HisTrap Buffer A1 (Ent5, Ent3, Ent1, NTD-CHC, mCherry)	20 mM NaP pH 7.5<br>500 mM NaCl<br>12.5 mM imidazole

HisTrap Buffer B1 (Ent5, Ent3, Ent1, NTD-CHC, mCherry)	20 mM NaP pH 7.5<br>500 mM NaCl<br>250 mM imidazole
HisTrap Buffer A2 (Sla2, Ede1, CHC$^{1172-1574}$, CLC, Ent1-ENTH)	30 mM HEPES pH 8<br>300 mM NaCl<br>5 % w/v glycerol
HisTrap Buffer B2 (Sla2, Ede1, CHC$^{1172-1574}$, CLC, Ent1-ENTH)	30 mM HEPES pH 8<br>300 mM NaCl<br>5 % w/v glycerol<br>200 mM imidazole
Dialysis Buffer 1 (Ent5, Ent3, Ent1, CHC-NTD, mCherry)	20 mM TRIS pH 7.5<br>200 mM NaCl<br>0.5 mM TCEP<br>optional: 1 mM EDTA
Dialysis Buffer 2 (Sla2, Ede1, CHC$^{1172-1574}$, CLC, Ent1-ENTH)	30 mM HEPES pH 8<br>150 mM NaCl<br>5 % w/v glycerol<br>1 mM DTT
SEC Buffer 1 (Ent5, Ent3, Ent1, mCherry)	100 mM Tris–HCl pH 7.5<br>150 mM NaCl<br>0.5 mM TCEP<br>1 mM EDTA
SEC Buffer 2 (NTD-CHC)	50mM Tris–HCl pH 9<br>150 mM NaCl<br>0.5 mM TCEP
SEC Buffer 3 (Sla2, Ede1, Ent1-ENTH, CLC, CHC$^{1172-1574}$)	30 mM HEPES pH 8<br>150 mM NaCl<br>0.5 mM TCEP

Supplementary Table 3. Primers used for QuikChange mutagenesis in both pETM-30-GST-NTD-CHC and pETM-11-Sumo3-Ent5-mEGFP

Name	Protein	Mutation	Type	Sequence
K63E_Fw	NTD-CHC	K63E	Fw	GGCAATGAAGTGACAAGGGAGAATATGGGCGGTGATTCTGCTATCATG
K63E_Rev	NTD-CHC	K63E	Rev	CATGATAGCAGAATCACCGCCCATATTCTCCCTTGTCACTTCATTGCC
I87D+Q89A+K98E	NTD-CHC	I87D+Q89A+K98E	Fw	CAAATGGTACTgacGTGgccATATTTAATTTGGAAACTAAGAGCgagTTAAAG
I87D+Q89A+K98E	NTD-CHC	I87D+Q89A+K98E	Rev	CTTTAACTCGCTCTTAGTTTCCAAATTAAATATGGCCACGTCAGTACCATTTG
Q195A+I197T_Fw	NTD-CHC	Q195A+I197T	Fw	CTCAAAACAACGTAACATCTCCgccGCTaccGACGGTCATGTTG
Q195A+II197T_Rev	NTD-CHC	Q195A+I197T	Rev	CAACATGACCGTCGGTAGCGGCGGAGATGTTACGTTGTTTTGAG
K251E_Fw	NTD-CHC	K251E	Fw	GCTTCATTGCCTTCTCAATATCAAGAGGAAACTACCGATATTTTCTTTCC
K251E_Rev	NTD-CHC	K251E	Rev	GGAAAGAAAATATCGGTAGTTTCCTCTTGATATTGAGAAGGCAATGAAGC
F26A_Fw	NTD-CHC	F26A	Fw	GGAATTTCCCCTCAATTCCTTGACGCCAGATCAACTACTTTCGAG
F26A_Rev	NTD-CHC	F26A	Rev	CTCGAAAGTAGTTGATCTGGCGTCAAGGAATTGAGGGGAAATTCC
Q155A_Fw	NTD-CHC	Q155A	Fw	CCTTGAGACACGCTAACTTGAACAATACCgccATTATCAATTTTGTGGCTAACAAAAACC
Q155A_Rev	NTD-CHC	Q155A	Rev	GGTTTTTGTTAGCCACAAAATTGATAATGGCGGTATTGTTCAAGTTAGCGTGTCTCAAGG
Delta249-287_Fw	Ent5	Delta Helix	Fw	GTGACCAACTTCAATGTTCCTGTGGAAACAGAAGATAGCATCCCTGACTT
Delta249-287_Rev	Ent5	Delta Helix	Rev	AAGTCAGGGATGCTATCTTCTGTTTCCACAGGAACATTGAAGTTGGTCAC
211-411_Fw	Ent5	IDR + Helix	Fw	caccattgacgtgttccagcaacagaccggtACGTCTGCAAGTTACGATAACATCG
211-411_Rev	Ent5	IDR + Helix	Rev	CGATGTTATCGTAACTTGCAGACGTaccggtctgttgctggaacacgtcaatggtg
1-210_Fw	Ent5	ENTH	Fw	CCATTTATAATGCCAACCAGATTTCAggatccgtgagcaagggcgaggagctgttc
1-210_Rev	Ent5	ENTH	Rev	gaacagctcctcgcccttgctcacggatccTGAAATCTGGTTGGCATTATAAATGG

References

1. Draper-Barr, G. *et al.* Sla2 is a core interaction hub for clathrin light chain and the Pan1/End3/Sla1 complex. *Structure* **33**, 1193–1207.e5 (2025).
2. Lizarrondo, J. *et al.* Structure of the endocytic adaptor complex reveals the basis for efficient membrane anchoring during clathrin-mediated endocytosis. *Nat. Commun.* **12**, 2889 (2021).
3. Burastero, O. *et al.* ChiraKit: an online tool for the analysis of circular dichroism spectroscopy data. *Nucleic Acids Res.* **53**, W158–W168 (2025).

Uncut gels

From Supplementary Figure 14a

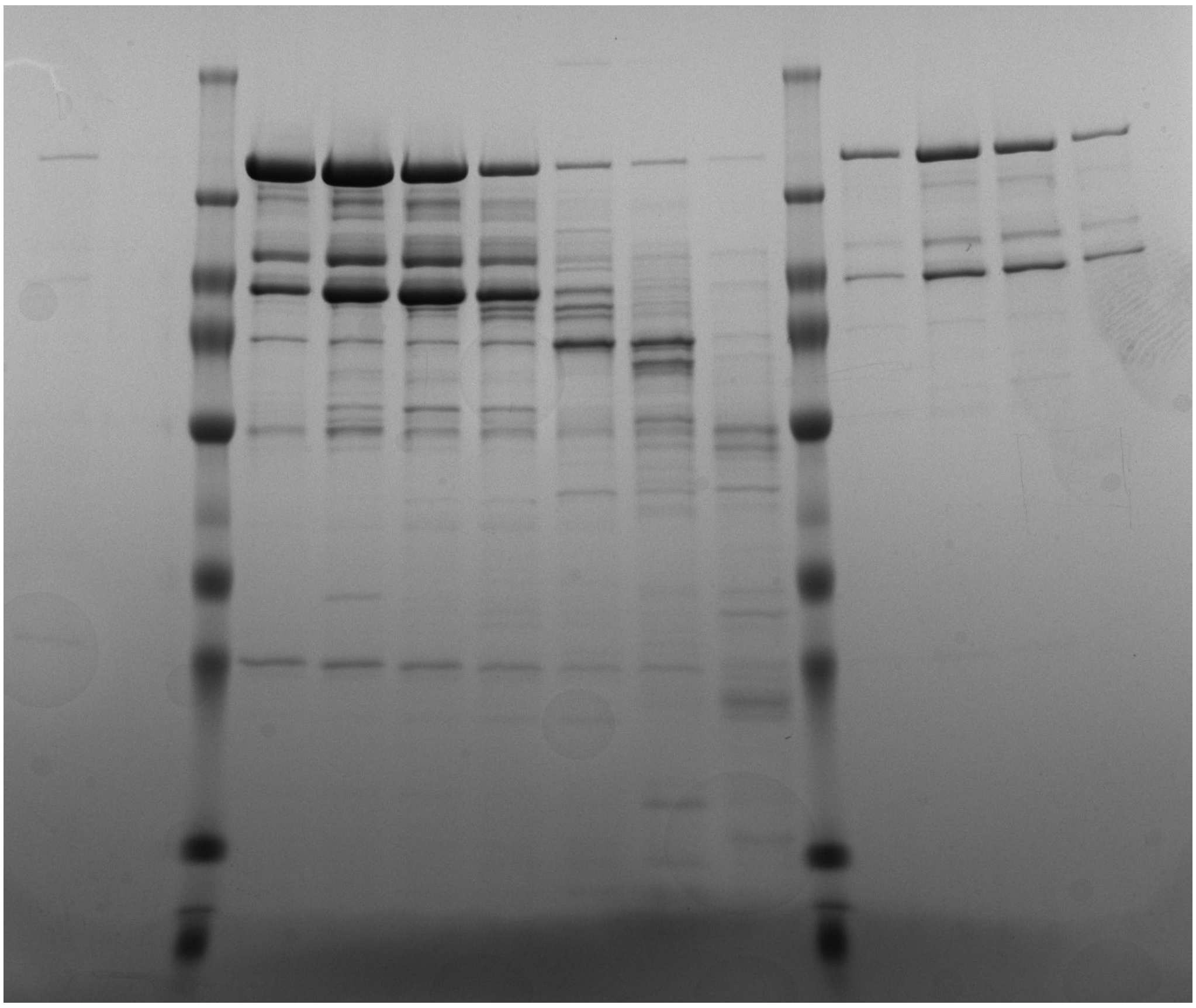

From Supplementary Figure 14d

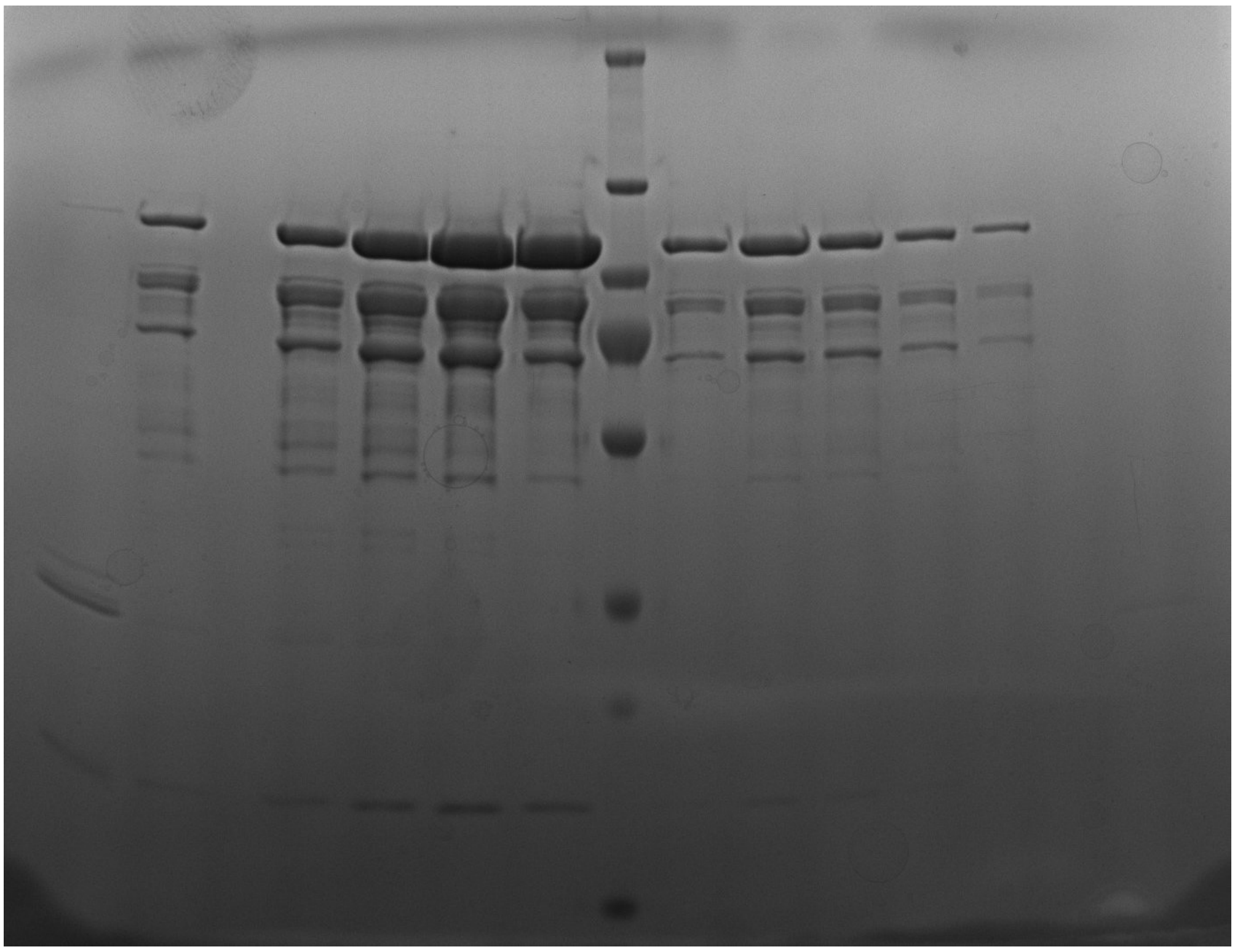

From Supplementary Figure 14f

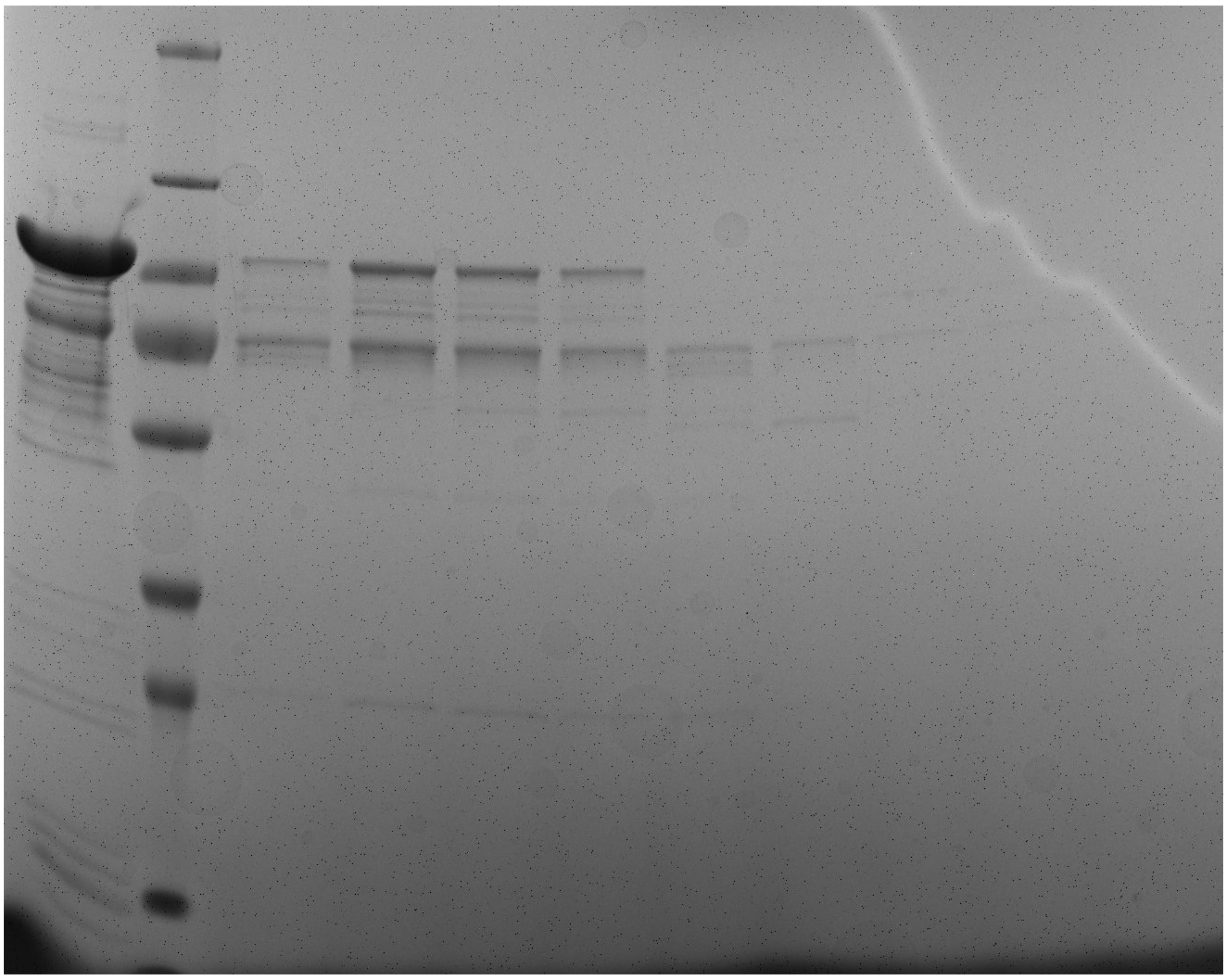

Supplement: Supplementary file 1 [file 42003_2026_11043_MOESM1_ESM.pdf]
