# Supplementary material for: Clathrin adaptors drive phase separation in endocytosis and trafficking
Source: Commun Biol. 2026 Sep 26;9:1260. doi: 10.1038/s42003-026-11043-9 (PMC13615978; doi:10.1038/s42003-026-11043-9)

Ent3-mCherry

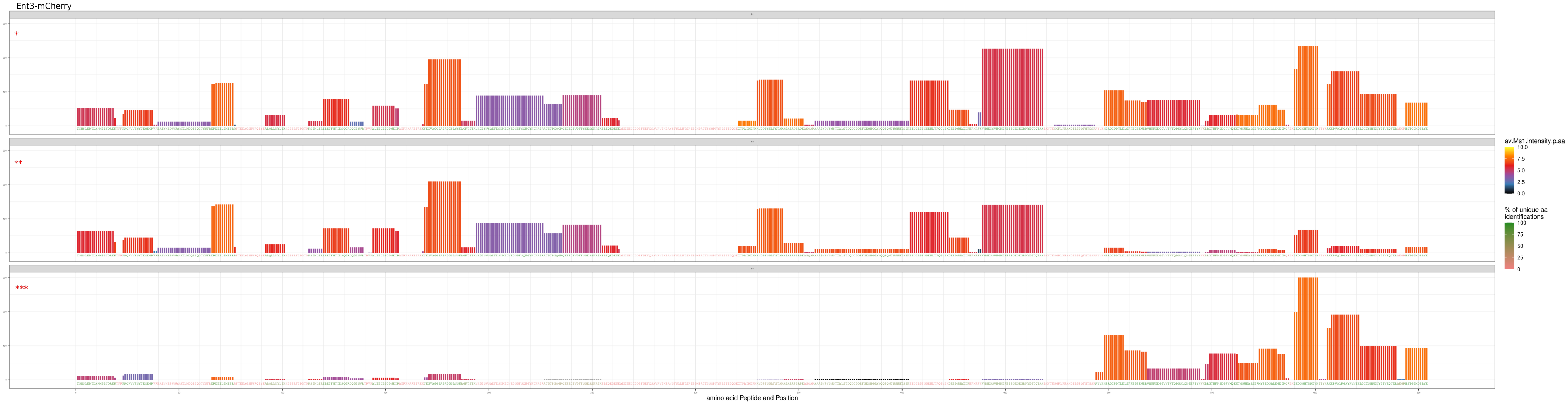

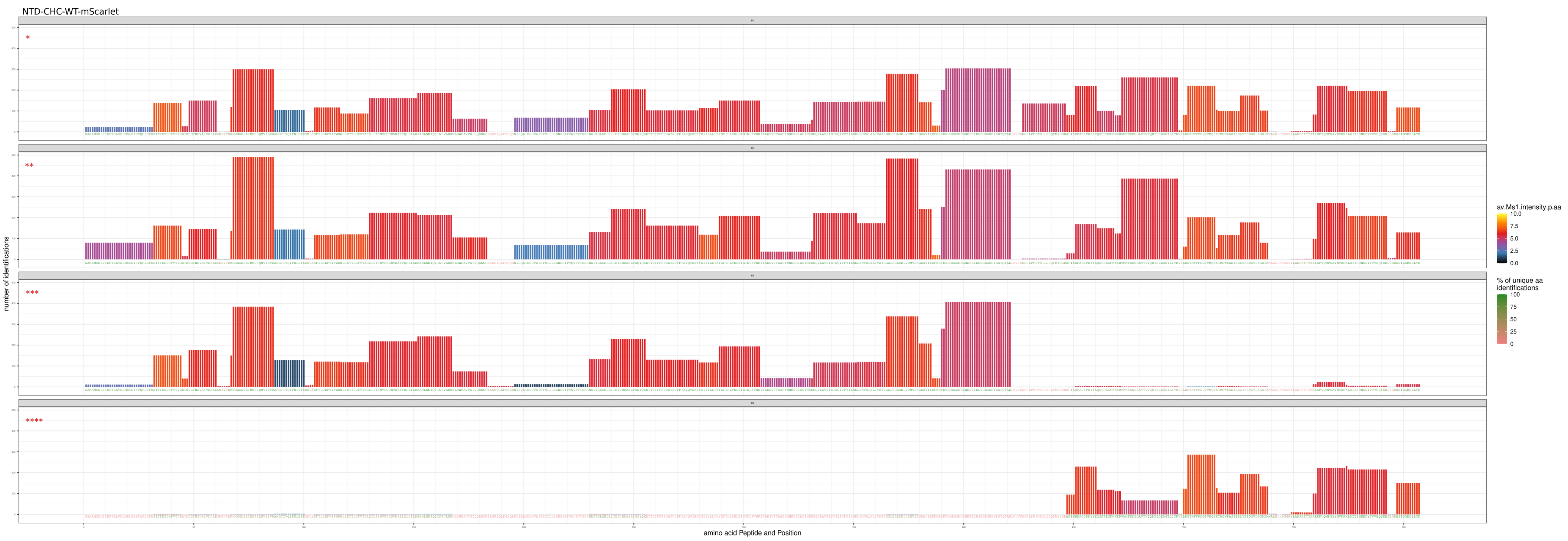
NTD-CHC-WT-mScarlet
*
**
***
****
number of identifications
amino acid Peptide and Position
av.Ms1.intensity.p.aa
10.0
7.5
5.0
2.5
0.0
% of unique aa identifications
100
75
50
25
0

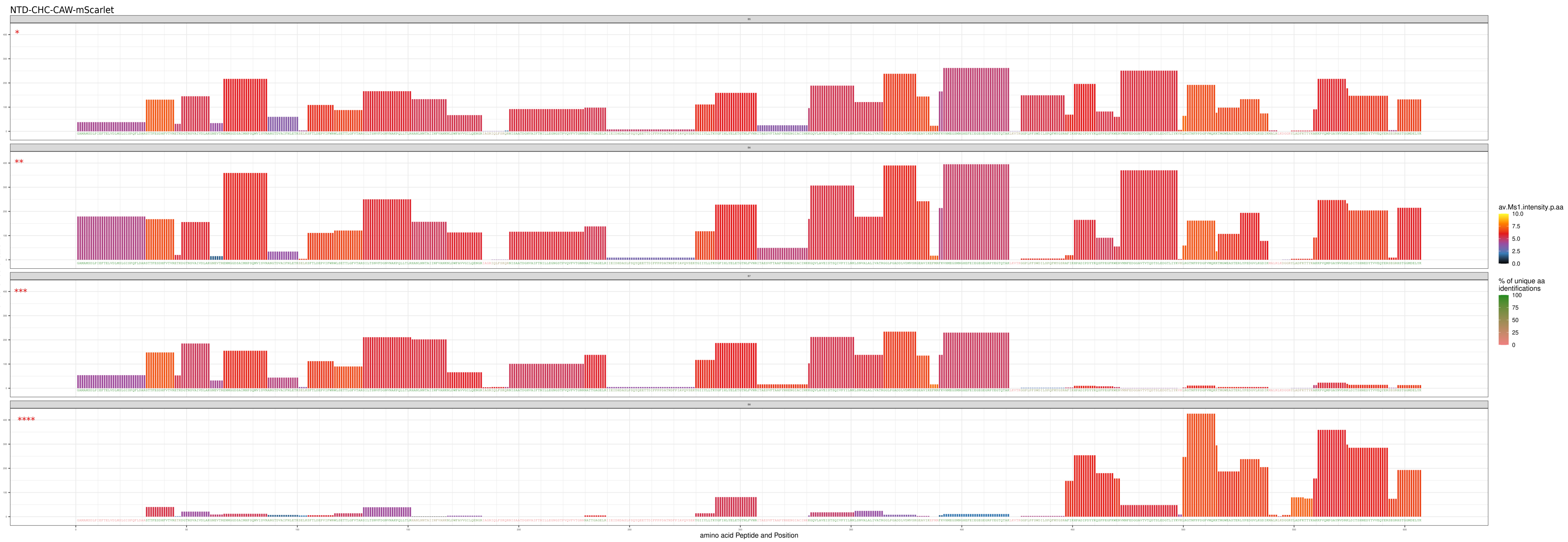
NTD-CHC-CAW-mScarlet
*
**
***
****
number of identifications
amino acid Peptide and Position
av.Ms1.intensity.p.aa
10.0
7.5
5.0
2.5
0.0
% of unique aa identifications
100
75
50
25
0

Sla2-FL-mEGFP

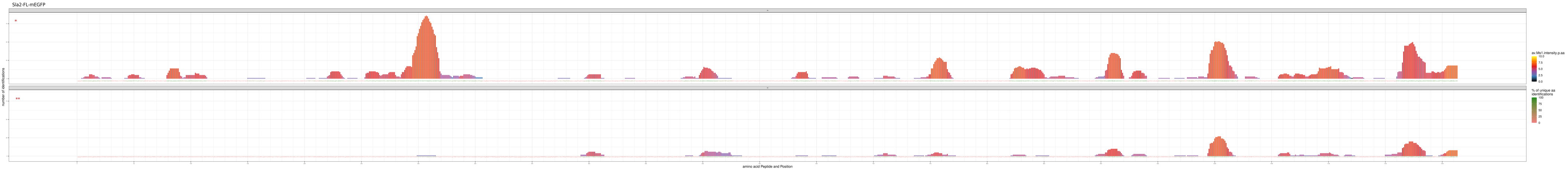

Sla2-IDR-CC-mScarlet

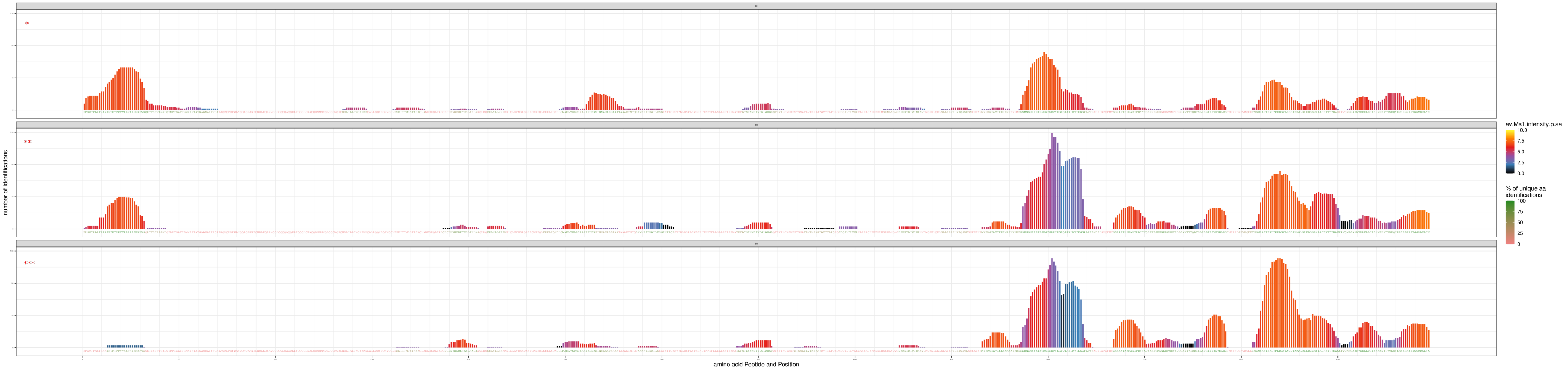

Ede1-IDR-CC-mNeonGreen

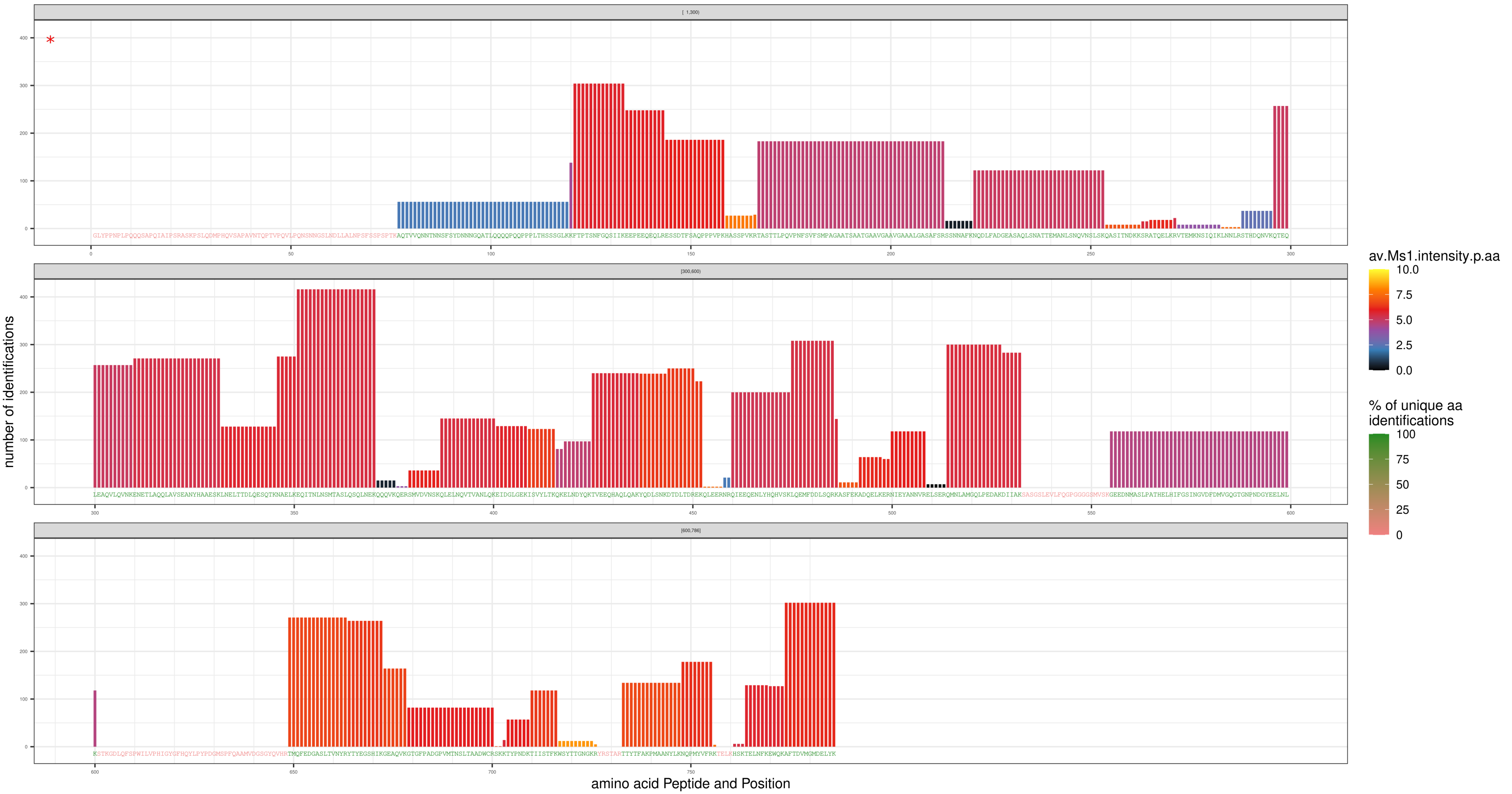

Supplement: Supplementary file 2 [file 42003_2026_11043_MOESM3_ESM.pdf]
