# Supplementary material for: Clathrin adaptors drive phase separation in endocytosis and trafficking
Source: Commun Biol. 2026 Sep 26;9:1260. doi: 10.1038/s42003-026-11043-9 (PMC13615978; doi:10.1038/s42003-026-11043-9)
Supplement: Supplementary file 3 [file 42003_2026_11043_MOESM4_ESM.pdf]

nature portfolio

Peer Review File

Clathrin adaptors drive phase separation in endocytosis and trafficking

Corresponding Author: Dr Maria Garcia-Alai

This file contains all reviewer reports in order by version, followed by all author rebuttals in order by version.

Version 0:

Reviewer comments:

Reviewer #1

(Remarks to the Author)
Reviewer comment:
Summary
In this manuscript, Draper-Barr et al. propose that clathrin adaptor proteins drive phase separation to organize endocytosis and intracellular trafficking. Using a combination of in vitro phase separation assays, quantitative partitioning measurements, biophysical characterization, and structural biology, the authors argue that adaptor proteins, particularly Ent5 and Sla2 undergo LLPS via intrinsically disordered regions and specific short sequence motifs.
The study addresses an important and timely question at the interface of membrane trafficking and biomolecular condensates. The supplementary data are extensive and technically sound. Overall, the work is compelling, but several conceptual and mechanistic issues should be addressed to strengthen the conclusions.
Major Comments
1. LLPS of protein phase separation:
To date, a large number of IDR-containing proteins have been reported to undergo phase separation. In this study, the authors first leverage the sequence features of clathrin adaptors, together with biochemical assays, to identify phase separation properties of adaptor proteins, including Ent5 and Sla2. Although the authors used assays such as FRAP to demonstrate LLPS properties of full-length Ent5 and the Sla2 IDR-CC construct (Figure 1c), I still have concerns and questions regarding the conditions under which phase separation occurs.
1) Why does Ent5 require very low salt (20 mM) conditions to undergo phase separation? Whether Ent5 undergoes robust phase separation on its own under physiological ionic strength and in the absence of crowding agents, and whether the observed behavior reflects a clear protein concentration dependence. Clarifying these points would strengthen the physiological relevance of the proposed LLPS mechanism. Besides, Ent5 is unlikely to act in isolation in vivo and may instead form condensates together with interacting adaptor proteins. How about it?
2) Compared with Ent5, Sla2 can undergo phase separation at 150 mM salt condition. Moreover, multiple domains contribute to promoting LLPS, including the ANTH domain, the IDR, and the coiled-coil domain. However, the manuscript does not discuss the potential relationship between Ent5 and Sla2 as adaptor proteins. Do these two proteins interact with each other, either directly or indirectly? If so, how might such interactions influence condensate formation? Moreover, do the distinct condensates formed by Ent5 and Sla2 correspond to different cargoes or distinct trafficking/endocytic pathways? Clarifying these relationships would strengthen the biological interpretation of the findings.
3) The Id/Is ratios are used to indicate protein enrichment within condensates. However, fluorescence intensity can also be leveraged to estimate the absolute protein concentration within droplets. I therefore suggest performing additional calibration-based measurements to determine the actual protein concentration in the condensates.
2. Mechanistic role of the Ent5 helical sequence:
The identification of a short α-helical element within the Ent5 IDR is intriguing and potentially novel. Circular dichroism and deletion analyses (Supplementary Figures 4 and 6) suggest that this region influences oligomerization and clustering behavior. However, the mechanistic role of this helix in phase separation remains ambiguous. Does the helix act as a sticker mediating homotypic interactions? Does the helix participate in intramolecular or intermolecular interactions? Can they design experiments provide direct evidence? I am curious whether, similar to what is shown in Figures 2e and 2f, adding the helix peptide to the Ent5Δ249–287 mEGFP mutant in the mass photometry (MP) experiments would be able to rescue the formation of higher-order oligomers.
Does it modulate LLPS indirectly by altering IDR conformational ensemble or effective valency? The authors should propose an explicit mechanistic model (even if speculative) for how this helical element contributes to LLPS.

3. GUV assays:
Giant Unilamellar Vesicles (GUVs) typically have diameters of around 10 μm and are sufficiently flat, making them a very good experimental system for mimicking cellular membranes. The authors have used GUVs as a vehicle for determining the recruitment of both Ent5 and Sla2 condensates to membrane environments (Figure 6). However, membrane environment typically lowers the critical concentration for LLPS by confining diffusion (PMID: 35411085, 32726575, 31604601).
1) In figure 6a-e, are the protein concentrations used by the authors representative of physiological conditions, or at least close to physiological levels in the lipidic condition? Because the protein concentrations used by the authors are not reduced by many orders of magnitude compared with those in solution.
2) Addition of condensates can happen on the surface of GUVs and also occurred in the solution. When membraneless organelles contact membrane-bound organelles, a wetting/prewetting behavior is often observed (PMID: 39385023, 37217523, 33473217). However, this phenomenon is not apparent in Figure 6b. In addition, the GUVs shown do not appear to consist of a single bilayer; instead, multilamellar stacking is observed (magenta channel). Could this be a consequence of the high protein concentration used in the experiment? Moreover, z-stack microscopy experiments should be performed, as they would provide stronger support for the proposed interpretation.
3) Finally, PIP_2 is generally a minor lipid component of the plasma membrane. In eukaryotic cells, its abundance is typically below 2%, and most in vitro reconstitution studies use ≤5% PIP_2 to approximate physiological conditions (PMID:15107463, 33202250, 29606581). In this study, however, 8% PIP_2 was used in the GUV experiments. The authors should justify the choice of this relatively high PIP_2 concentration. Excessive PIP_2 may substantially alter membrane electrostatics, surface potential, and other biophysical properties of the bilayer, potentially leading to non-physiological behavior. It would therefore be important to clarify whether the observed effects depend quantitatively on PIP_2 content, and whether similar results can be obtained at lower, more physiological PIP_2 fractions. Otherwise, there is a concern that the experimental conditions may be overly artificial.
4. Discussion Language:
In line 393, "This helix alone is insufficient to promote condensation, but together with the surrounding disordered region and the ENTH domain, it enables robust LLPS at physiological concentrations." should be toned down. The authors state that Ent5 undergoes LLPS at physiological concentrations. However, the manuscript does not provide any quantitative information regarding the physiological abundance or concentration of Ent5 in yeast, nor is this discussed with reference to existing literature. Or the authors should therefore explicitly state the physiological abundance/concentration of Ent5 in yeast (with appropriate citation or measurement); discuss how the in vitro concentrations used relate to the estimated in vivo levels; if membrane binding and PIP_2-dependent recruitment are proposed to elevate the local effective concentration of Ent5 to the phase separation regime, this should be quantitatively justified or experimentally supported. Clarifying these points is essential to substantiate the claim that Ent5 undergoes LLPS under physiologically relevant conditions.

Minor Comments
1. The biochemical experiments described in the manuscript employ various full-length proteins and truncated variants. Please provide protein purification data or representative gel images (e.g., SDS-PAGE) to demonstrate protein quality and integrity.
2. Fluorescent tags can be sufficiently large to influence phase behavior. Indeed, figure S3 indicates that fluorescent tags can promote phase separation. However, to avoid potential artifacts, it would be preferable to also employ small hydrophobic probes to investigate LLPS and confirm that the observed phase behavior is not tag-induced.
2. Line 805, "White scale bars (5 μm) in panels b and d indicate the scale for all microscopy images." Correction: they may be referring to panels d and f.
3. Correction: the Figure 2 contains two panels labeled g, and one of them should be corrected to panel h.
4. I suggest labeling Figure 7b directly as Ent5Δhelix, which would improve the visual clarity and readability of the data. Previous studies have reported that Gga2 interacts with Ent5 (PMID: 22457357 and 20704189). In the same context as Figure 7b, it would be important to ask whether the use of the Ent5Δhelix mutant affects the ability of Ent5 to bind Gga2. If so, could the observed prolongation of lifetimes be a consequence of impaired Gga2–Ent5 interaction?

Overall Assessment
This is a strong and carefully executed study that makes a significant contribution to the emerging understanding of LLPS in endocytosis. Addressing the points above-particularly those concerning mechanism and conceptual clarity-would substantially strengthen the manuscript and its impact.

Recommendation:
Minor to moderate revision.

Reviewer #2

(Remarks to the Author)
Authors present a study highlighting the role of two distinct clathrin adapters in promoting condensate formation, providing a link between clathrin recruitment to a membrane site and phase separation. The work focuses on Ent5 and Sla2, both of which contain N-terminal phosphoinositide-binding domains and either an intrinsically disordered region (Ent5) or a coiled-coil followed by an actin-binding domain (Sla2). These proteins are used as structure–function examples to support the broader idea that adaptor-driven LLPS underlies clathrin-mediated endocytosis.

The study screened for the role of LLPS using in silico approach and possibly chemical follow ups, but screen data are not shown or extremely challenging for this reviewer to find (??). Ent5 and Sla2 were then subjected to standard in vitro LLPS assays, which generally supported the predictions. Interestingly, MP experiments were performed alongside SAXS. These

provided some limited evidence for higher MW species. Further experiments tested client/driver hypotheses, ending with a test of the effect of the helix of Ent5 that seemed to drive LLPS. The outcome was limited.

Conceptually, the study shows in vitro protein experiments suggestive of LLPS behavior, but without the necessary biochemical controls requisite for most in vitro protein biochemistry work, especially reconstitution. The LLPS field is plagued with multiple tagged constructs, no purification information, and generally biochemistry that is not of acceptable quality to support conclusions drawing on sophisticated physical phenomenon. In this case, I would generally trust that the biochemical reagents are properly assessed (there is a structure even), but it is not shown. Taken as granted, the results are novel and supportive of some data in the literature suggesting the roles of clathrin adaptors linking endocytosis to phase separation. Some molecular data is presented in support of this, and the findings are novel.

That distinct structures including IDR and CC can take part in LLPS in vitro is undisputed. The finding of "robust LLPS at physiological concentration" doesn't yet apply until we know the in vivo concentration. And there is I think the major problem. The purpose of this behavior is not clearly described nor pursued. So, a phenomenon which is observed in vitro, and supported from a couple orthogonal angles, has a challenging route for impact.

The manuscript does not appear ready for publication. The manuscript reads as a thesis-style catalog of proteins and domains rather than a coherent narrative, includes discussion of proteins not pursued experimentally, and is often overly didactic. Some 'lab lingo' used that needs cleanup (e.g. "the helix").

Major

The standard for structural protein biochemistry requires the relevant purification details: protein gels, size-exclusion chromatograms, and ideally an independent measure of the protein size (DLS, native, MP, etc). Size-exclusion chromatography should remove aggregates without reliance on centrifugation; detectable aggregation would indicate poor protein behavior (Methods, Recombinant Protein Purification). Authors should show this information.

Mass photometry experiments draw conclusions from n=1 singular insets, despite triplicate measurements. They show very little difference. Technical factors can drive these observations, please refer to https://www.nature.com/articles/s41596-025-01255-4 (unfortunately not field-representative as a single lab published it, but we have replicated many of the findings). Conclusions should be toned down – the reported higher-molecular-weight species appear at extremely low abundance (on the order of ~0.01% of particles, corresponding to only 2–3 particles per cluster), raising questions about their significance. Authors should comment.

Standard LLPS experiments were performed. Purify, incubate, observe droplets, FRAP, domain dissection, mix, conclude LLPS. What is the biological consequence and significance? Do we have concentration, salt, detergent dependence? Crowding agents? The experiments feel somewhat underwhelming in impact. Authors should make clear

Sla2 recruits to Ede1 via "cc-interactions". What are these? Is coiled coil packing meant, i.e. h-b-c-h-d-e-f? That could be oligomerization, well understood to be a challenge of working with coiled coils at reagent concentrations. Authors need to specifically address what is meant and evidence it more clearly.

The structure is barely connected to the rest of the manuscript. I don't see the point. Multivalency? Avidity? The authors need to rethink how this reads.

Assumptions from quite old literature have led to the view that certain ENTH domains bind PI4,5P2 and some PI3,5P2. This would be best explicitly tested with modern approaches as in Fig. 6. I don't think this has been systematically done. What if there is no PI35P2? Or PI45P2? These should be explicitly tested and controlled for. The GUV experiments are unfortunately rather haphazard and non-systematic.

Similarly, it is admirable how forthright the authors are on concentrations. However, these appear to vary from place to place, and it is very difficult to conceptualize as currently presented. Authors should not necessarily repeat everything systematically, but consider how the presentation currently looks.

Finally, and in positive spirit, a major challenge remains unresolved: how a ~50 nm coiled coil dimer, exceptionally stable and likely quite rigid, undergoes LLPS. How should one conceive the internal structure of such a droplet? I am not aware of any in vivo or in vitro structural evidence for how these assemblies can be conceived. Moreover, if CC interactions drive LLPS then we would expect turbidity increase with time and irreversible aggregation akin to amyloid/TDP43 aggregation, rather than reversible condensates. Authors should spend time addressing this concept, as it would be central to one of the two adaptors investigated here.

Minor

Should cite the below re: Ent5 binding PI35P2 (current citations 17 Duncan, and 18 Eugster): Chidambaram et al. Journal of Biological Chemistry, Volume 279, Issue 6, 2004, Pages 4175-4179, ISSN 0021-9258, https://doi.org/10.1074/jbc.M308667200

Target selection from the screen is not clear.

Fig 1 should say "in vitro" in the title and in legend should say "droplets existed at" rather than formed, as no concentration dependent experiments were performed.

Fig 2 has two 'g' panels. "The helix" should define precisely the construct used (Ent5249-287, I believe).

Use of PEG8000 not justified anywhere and alternative crowding not employed.

Fig 3 – what are the statistic here? What is reproducibility of such experiments?

Structural descriptions page 7 – what is significance?
Pg. 9 data not shown should be shown. Where is the PI45P2 data?
Simply as a curiousity, have the authors considered Pegylated lipids vs. soluble PEG as crowding agent?
Fig 6 no panel f present but referenced in legend

Reviewer #3

(Remarks to the Author)
This study is based on an in silico screen for clathrin adaptors with predicted IDRs, that might undergo phase separation. Select candidates were tested for their ability to induce LLPS in vitro. Three of these candidates (Ent5 and Sla2 as validated in this study; Ede1 also based on previous observations) were analyzed in more detail. Indeed, isolated Ent5 and Sla2 can undergo phase separation in vitro. In case of Ent5 this property is further shown to require an α-helix that acts as a sticker. Subsequent experiments reveal that Ent5 can recruit NTD-clathrin into droplets, and that Ede1-droplets can recruit Sla2. Furthermore, the authors demonstrate that Ent5 and Sla2/Ede1-ENTH can form condensates on PI(3,5)P2- and PI(4,5)P2-enriched membranes, respectively. Finally, the impact of Ent5-dependent LLPS was investigated in living yeast, where loss of a crucial α-helix present in Ent5 is shown to delay the release of Gga2-dependent coats.

The study summarizes very interesting findings on the phase separating properties of a variety of clathrin-associated adaptor proteins, and suggests important novel concepts on how membrane binding, adaptor interactions and clathrin recruitment might be coordinated by condensation. However, a few aspects need to be addressed to render the manuscript suitable for publication in Communications Biology.

Major questions and concerns:

1) The authors need to show the purity of all recombinant proteins used for this study.
2) On page 6 the authors claim that "Ent5 α-Helix showed significantly less signal above 200 kDa". How has significance been determined? What is the P value?
3) According to Figs. 1/3 Sla2 IDR-CC forms droplets at 20 µM upon the addition of crowding agent (2.5% PEG 8000). For the data depicted in Figure 5, however, neither results section, nor legend indicate that PEG was added. This difference needs to be explained.
4) Also, I´m confused about the identity of the Sla2 IDR-CC. Based on Fig. 1a/b one would suspect that it covers aa260-560. According to the sketch shown in Fig. 3c/d, however, the CC additionally contains the REND domain. Have the same protein fragments been used for the two experiments? Then the text needs to be adapted accordingly.
5) Where relevant the authors need to indicate numbers of experiments/numbers of droplets per experiment underlying their statistics.
6) In Fig. 5 Ede1 IDR-CC is applied at a concentration of 5 µM, based on a paper that showed condensate formation of the homologous region in human Eps15 (ref. 27). Would 5 µM be sufficient for yeast Ede1 IDR-CC as well? And would Sla2 IDR-CC maybe act as a driver at 5 µM Ede1 IDR-CC?
7) Fig.6 shows that Sla2, in concert with Ent1-ENTH, forms condensates in presence of crowding agent, but unlike Ent5 does not deform membranes. Would membrane deformation occur in presence of full-length Ent1? Would phase separation be affected?
8) Related to this: The discussion indicates that in contrast to initial computational predictions Ent1, Ent2 or Ent3 did not form condensates in a chemical screen. The data are apparently missing in the manuscript, and also not mentioned in the results section. They should be included.

Minor points:

a) On page 7 the authors indicate that "the concentration of Ent5 decreases in the presence of NTD-CHC". This wording is misleading. Also, the authors should refer to Fig. 4d in the results text, where the relevant quantitative data are depicted. This would greatly assist the reader.
b) Table 1: To my knowledge membrane recruitment of Gga proteins is promoted by PI(4)P, in mammals as in yeast (Daboussie et al., 2016, PMID 22344030). In mammals the GAT domain has been shown to be sufficient for PI(4)P binding and recruitment to liposomes (Wang et al., 2007, PMID 17494868). The respective entries in the table should be corrected.
c) Headline on page 9: contains a spelling error (dependent). Also, the sentence is not really consistent with the actual results: The Ent5 helix rather affects Gga2-dependent coating. Cargo trafficking has not been monitored (though this would be an interesting readout).
d) Text section, as well as legend to fig.7 should indicate the organism the experiment has been conducted with.
e) Page 10, end of results section: the presence of the Ent5 helix affects release dynamics from Gga2-positive structures, rather than its recruitment dynamics to these structures.

Remark:
The significance of this manuscript is diminished by a lack of coherence between the different experiments. For instance, the authors demonstrate that Ent5 forms liquid condensates (Figs. 1,2), and drives NTD-CHC or Ent3 recruitment into Ent5-droplets (Fig.4). But it remains unclear if NTD-CHC or Ent3 would also be recruited to Ent5 condensates on PI(3,5)P2-enriched GUVs (Fig.6). On the other hand, the live imaging approach investigates Ent5 recruitment to Gga2-positive Golgi structures (Fig.7). But these structures are most likely enriched in PI(4)P, and it is not further assessed if (membrane-

associated) Ent5 could drive Gga2 into condensates (or vice versa: according to Table 1 Gga2 is predicted to undergo phase separation itself).
Similarly, it is shown that Sla2/Ent1-ENTH forms condensates on PI(4,5)P2-enriched GUVs in presence of crowding agents (Fig.6). But it is not investigated, if and how this process would be modulated if Ede1 or clathrin would be added, which are shown to act as drivers or clients of Sla2 condensation, respectively (Fig.5).
The study would greatly benefit if such inconsistencies could be resolved, and maybe reach an even wider field.

Version 1:

Reviewer comments:

Reviewer #1

(Remarks to the Author)
I appreciate the authors' thorough and thoughtful responses to my previous comments. Overall, the revised manuscript has improved substantially in clarity, terminology, experimental documentation, and mechanistic framing. Most of my concerns have been addressed satisfactorily. However, I still have a few remaining comments:

1.The new phase-diagram measurements and the lower-concentration GUV experiments are helpful additions. They provide further support for the effects of protein concentration, crowding, and membrane recruitment on Ent5 condensation. The authors have also been more careful in discussing the limitations of the GUV experiments and have toned down their conclusions about physiological relevance, membrane topology, and membrane remodelling.

2. I also find the revised discussion of the Ent5 249-287 region more balanced. The data are consistent with this region acting as a sticker through intermolecular interactions, while the exact molecular contacts remain unknown. The additional protein-quality controls, methodological details, replicate information, and revised analysis of the Gga2-associated events have further improved the manuscript.

3. I appreciate the authors' attempt to examine peptide-induced oligomerization by mass photometry. I do not think this experiment can be interpreted conclusively. The expected molecular mass of the isolated Ent5(249-287) peptide is only about 4-5 kDa, whereas the peptide-only sample gives a strong signal at around 90-100 kDa. This could reflect peptide aggregation, contamination, measurement background, or an effect of the peptide concentration used. The peptide-protein mixture appears to shift toward approximately 120-130 kDa, but this increase is much larger than expected from the binding of a single peptide and therefore does not demonstrate the formation of a defined peptide-protein complex. The different sample concentrations and total event counts also make direct comparison of the distributions difficult. I don't know what happens using the helix peptide. But I am curious as to why this result appears inconsistent with the LLPS assay, in which addition of the helix peptide was sufficient to restore phase separation of Ent5Δ249-287. One possible explanation is that the helix acts as a sticker only when its local concentration increases within the condensed phase, whereas the corresponding interactions are too weak or transient to be detected in the aqueous dilute phase. It would be helpful if the authors could comment on this possibility.
I do not think that another experiment is essential for publication, as long as the mass photometry result is clearly described as inconclusive and is not used to support a specific oligomeric state or binding stoichiometry. If peptide-induced oligomerization remains an important part of the proposed mechanism, it should be confirmed using an independent and appropriately controlled method. Otherwise, the wording throughout the manuscript should be toned down to make clear that peptide-induced oligomerization remains a possible explanation rather than a conclusion directly supported by the current data.

4. The presentation of Supplementary Fig. S2g could be improved.

5. The manuscript would benefit from one final round of proofreading. I noticed a few duplicated words, spacing problems, and other minor formatting issues.

Overall, the authors have addressed my main scientific concerns. The remaining points are minor and do not change my positive view of the study. I therefore support publication after minor revision.

Reviewer #2

(Remarks to the Author)
The authors have addressed all concerns with utmost rigor. While the length of the manuscript has grown somewhat as a result, the points of this reviewer were addressed.

Reviewer #3

(Remarks to the Author)
The authors have addressed all important comments and concerns. The revised manuscript is now acceptable for publication.

Open Access This Peer Review File is licensed under a Creative Commons Attribution 4.0 International License, which permits use, sharing, adaptation, distribution and reproduction in any medium or format, as long as you give appropriate credit to the original author(s) and the source, provide a link to the Creative Commons license, and indicate if changes were made.
In cases where reviewers are anonymous, credit should be given to 'Anonymous Referee' and the source.
The images or other third party material in this Peer Review File are included in the article's Creative Commons license, unless indicated otherwise in a credit line to the material. If material is not included in the article's Creative Commons license and your intended use is not permitted by statutory regulation or exceeds the permitted use, you will need to obtain permission directly from the copyright holder.
To view a copy of this license, visit https://creativecommons.org/licenses/by/4.0/

Reviewer #1 (Remarks to the Author):

Reviewer comment:
Summary
In this manuscript, Draper-Barr et al. propose that clathrin adaptor proteins drive phase separation to organize endocytosis and intracellular trafficking. Using a combination of in vitro phase separation assays, quantitative partitioning measurements, biophysical characterization, and structural biology, the authors argue that adaptor proteins, particularly Ent5 and Sla2 undergo LLPS via intrinsically disordered regions and specific short sequence motifs.
The study addresses an important and timely question at the interface of membrane trafficking and biomolecular condensates. The supplementary data are extensive and technically sound. Overall, the work is compelling, but several conceptual and mechanistic issues should be addressed to strengthen the conclusions.

Major Comments
1. LLPS of protein phase separation:
To date, a large number of IDR-containing proteins have been reported to undergo phase separation. In this study, the authors first leverage the sequence features of clathrin adaptors, together with biochemical assays, to identify phase separation properties of adaptor proteins, including Ent5 and Sla2. Although the authors used assays such as FRAP to demonstrate LLPS properties of full-length Ent5 and the Sla2 IDR-CC construct (Figure 1c), I still have concerns and questions regarding the conditions under which phase separation occurs.

1) Why does Ent5 require very low salt (20 mM) conditions to undergo phase separation? Whether Ent5 undergoes robust phase separation on its own under physiological ionic strength and in the absence of crowding agents, and whether the observed behavior reflects a clear protein concentration dependence. Clarifying these points would strengthen the physiological relevance of the proposed LLPS mechanism. Besides, Ent5 is unlikely to act in isolation in vivo and may instead form condensates together with interacting adaptor proteins. How about it?

We thank the reviewer for this comment and agree that this point was not sufficiently clarified in the original manuscript.

Ent5 undergoes phase separation in the presence of the macromolecular crowding agent PEG 8000 at physiological ionic strength (150 mM NaCl). To clarify this, we have added a new supplementary figure (Supplementary Fig. S2 h) showing phase separation monitored by absorbance as a function of both Ent5 concentration and PEG 8000 concentration at 150 mM NaCl. These data demonstrate that Ent5 retains the capacity to undergo LLPS under physiological ionic strength when molecular crowding is present.

During our initial screen for phase-separation conditions, we also observed that Ent5 undergoes LLPS in the absence of crowding agents when the ionic strength is reduced (Supplementary Fig. S2 g). We subsequently performed mechanistic experiments under these conditions because they enabled complementary biophysical analyses, including stopped-flow kinetics and SAXS. The presence of PEG 8000 would have substantially increased viscosity and background scattering, complicating these measurements.

The newly included data further demonstrate that Ent5 phase separation depends on both protein concentration and ionic strength. Increasing protein concentration promotes condensate formation at low ionic strength, whereas increasing NaCl concentration suppresses LLPS in the absence of crowding agents. These observations are consistent with a contribution of electrostatic interactions to Ent5 condensation.

We agree with the reviewer that Ent5 is unlikely to act in isolation *in vivo*. Endocytic and trafficking adaptors function within highly interconnected interaction networks, and it is likely that heterotypic interactions with binding partners further contribute to condensate formation and regulation. Indeed, our data show that the ENTH domain and the C-terminal region of Ent5 cooperate to enhance condensation, and that clathrin is efficiently recruited into Ent5 condensates. Defining the contribution of additional adaptor proteins to condensate assembly will be an important direction for future work. We have expanded the Discussion to clarify these points.

“Our findings suggest that Ent5 may act as a driver of phase separation in endosomal trafficking given its capacity for recruiting NTD-CHC and, to a lesser degree, Ent3. Importantly, Ent5 is unlikely to act in isolation in vivo. Endocytic and trafficking adaptors operate within highly interconnected interaction networks, and heterotypic interactions with partner proteins are likely to contribute substantially to condensate formation and regulation. The cooperative behaviour we observe

between the ENTH domain and the C-terminal region of Ent5, together with the efficient recruitment of clathrin into Ent5 condensates, supports a model in which condensates emerge from multiple weak interactions among adaptors, coat proteins, and membranes rather than from the self-association of a single component. Future studies will be required to define how additional adaptor proteins contribute to the assembly, composition, and dynamics of these condensates."

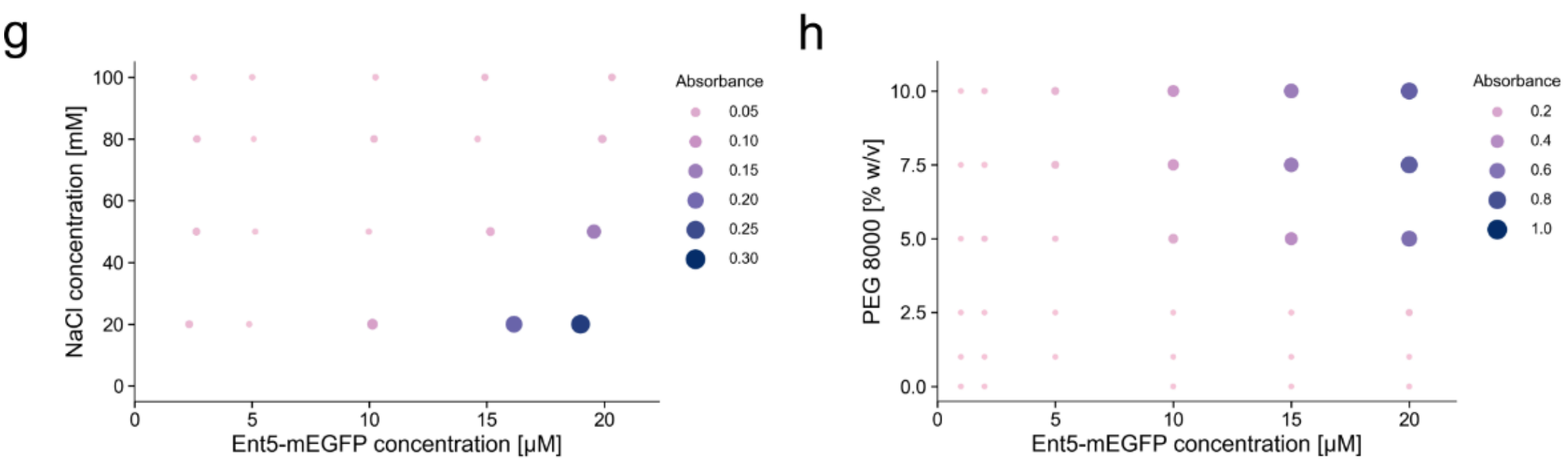

Supplementary Figure 2 (g,h) Phase diagrams of Ent5-mEGFP phase separation as a function of protein concentration and either NaCl concentration (g) or PEG 8000 concentration (h). Phase separation was quantified by buffer-corrected absorbance/scattering at 600 nm using a CLARIOstar plate reader in a 384-well plate. Circle size and colour represent the absorbance values. Measurements were performed at 21 °C and show the mean of three independent experiments. In (g), NaCl concentration was varied by dilution. In (h), NaCl concentration was maintained at 150 mM while PEG 8000 concentration was varied. Unless otherwise indicated, samples contained 100 mM Tris-HCl (pH 7.5) and 0.5 mM TCEP.

2) Compared with Ent5, Sla2 can undergo phase separation at 150 mM salt condition. Moreover, multiple domains contribute to promoting LLPS, including the ANTH domain, the IDR, and the coiled-coil domain. However, the manuscript does not discuss the potential relationship between Ent5 and Sla2 as adaptor proteins. Do these two proteins interact with each other, either directly or indirectly? If so, how might such interactions influence condensate formation? Moreover, do the distinct condensates formed by Ent5 and Sla2 correspond to different cargoes or distinct

trafficking/endocytic pathways? Clarifying these relationships would strengthen the biological interpretation of the findings.

We thank the reviewer for raising this point. Ent5 and Sla2 function in distinct trafficking pathways and, to our knowledge, there is currently no evidence for a direct interaction between them. Ent5 functions primarily in trans-Golgi network (TGN) and endosomal trafficking, whereas Sla2 is a core component of the endocytic machinery at the plasma membrane.

Although both proteins contain phosphoinositide-binding domains, they have been associated with different membrane environments. Sla2 is recruited to the plasma membrane through interactions involving PI(4,5)P_2, whereas Ent5 has been linked to endosomal and TGN membranes that are enriched in PI(3,5)P_2 and related phosphoinositides. Consistent with these distinct cellular localisations, Ent5 and Sla2 are expected to participate in different protein interaction networks and to contribute to condensates with different compositions and biological functions. We have clarified this point in Table 1.

The differences in phase behaviour observed for Ent5 and Sla2 may also reflect differences in their domain architectures and interaction networks. Sla2 contains multiple structured domains, including an ANTH domain and a coiled-coil-REND containing region, which increase its potential interaction valency and may contribute to its ability to undergo phase separation under higher ionic-strength conditions. In contrast, our results suggest that Ent5 phase separation is more sensitive to ionic strength and may rely to a greater extent on interactions mediated by its intrinsically disordered region.

More broadly, we anticipate that the condensates formed by Ent5 and Sla2 represent distinct assemblies associated with different stages and locations of membrane trafficking rather than a common adaptor condensate.

3) The Id/Is ratios are used to indicate protein enrichment within condensates. However, fluorescence intensity can also be leveraged to estimate the absolute protein concentration within droplets. I therefore suggest performing additional calibration-based measurements to determine the actual protein concentration in the condensates.

We thank the reviewer for this suggestion. We agree that calibration-based fluorescence measurements could, in principle, be used to estimate protein concentrations within condensates and provide additional quantitative information.

However, obtaining reliable absolute concentration estimates requires careful calibration of each fluorophore under conditions comparable to those used in the LLPS experiments. In dense condensates, fluorescence intensity may not scale linearly with protein concentration due to effects such as self-quenching, altered fluorophore environments, and optical artefacts arising from the high local protein density. Consequently, absolute concentration estimates derived from fluorescence measurements can be associated with substantial uncertainty. Furthermore, the use of different fluorescent tags throughout the study (mEGFP, mCherry and mScarlet) would require independent calibration for each construct and experimental setup.

In the present study, our conclusions rely on comparative changes in partitioning, quantified using I_D/I_S ratios, rather than on the absolute concentration of proteins within condensates. These measurements provide a robust and internally consistent metric for assessing protein enrichment and the effects of interaction partners on condensate formation. While calibration-based concentration measurements would be an interesting extension of this work, we do not believe they are required to support the conclusions of the current manuscript.

2. Mechanistic role of the Ent5 helical sequence:
The identification of a short α-helical element within the Ent5 IDR is intriguing and potentially novel. Circular dichroism and deletion analyses (Supplementary Figures 4 and 6) suggest that this region influences oligomerization and clustering behavior. However, the mechanistic role of this helix in phase separation remains ambiguous. Does the helix act as a sticker mediating homotypic interactions? Does the helix participate in intramolecular or intermolecular interactions? Can they design experiments provide direct evidence? I am curious whether, similar to what is shown in Figures 2e and 2f, adding the helix peptide to the Ent5Δ249–287 mEGFP mutant in the mass photometry (MP) experiments would be able to rescue the formation of higher-order oligomers.
Does it modulate LLPS indirectly by altering IDR conformational ensemble or effective valency? The authors should propose an explicit mechanistic model (even if speculative) for how this helical element contributes to LLPS.

We thank the reviewer for this constructive comment. We agree that the mechanistic role of the α-helical element within the Ent5 IDR deserves further discussion.

Our data are most consistent with a model in which residues 249–287 act as a sticker-like element that contributes interaction valency and promotes LLPS. This interpretation is based on several observations. First, deletion of residues 249–287

(Ent5Δ249–287-mEGFP) strongly impairs phase separation, substantially increasing the saturation concentration (C_{sat}) required for condensate formation. Second, the isolated peptide corresponding to residues 249–287 does not undergo phase separation on its own, even at concentrations up to 500 µM. Third, addition of the isolated peptide restores condensate formation by Ent5Δ249–287.

The ability of the isolated peptide to rescue condensation in trans argues that this region contributes more than a purely intramolecular structural effect on the conformational ensemble of the full-length protein. Instead, it suggests that residues 249–287 participate in intermolecular interactions that increase the effective valency of Ent5 and thereby promote LLPS. At present, however, the molecular nature of these interactions remains unresolved. The current data do not allow us to distinguish between interactions involving the 249-287 helix and other regions of Ent5 and direct homotypic helix–helix interactions.

We have therefore revised the manuscript to propose the following working model in the Results section:

“Together, these results support a model in which residues 249–287 function as a sticker-like element within Ent5. Although neither the isolated peptide nor Ent5Δ249–287 undergoes efficient phase separation individually, combining both components restores condensate formation. This activity in trans is consistent with a role for the helical region in increasing the effective interaction valency of Ent5 through transient intermolecular contacts. However, the molecular identity of these contacts remains to be determined, and both heterotypic interactions with other regions of Ent5 and homotypic helix–helix interactions remain possible.”

Following the reviewer’s suggestion, we also attempted to assess whether addition of the isolated peptide altered the oligomeric distribution of Ent5Δ249–287-mEGFP by mass photometry. Unfortunately, this experiment proved difficult to interpret because the peptide alone generated a substantial background signal that overlapped with the mass range of interest, preventing reliable quantification of peptide-induced changes. For completeness, we provide the corresponding mass photometry figure below.

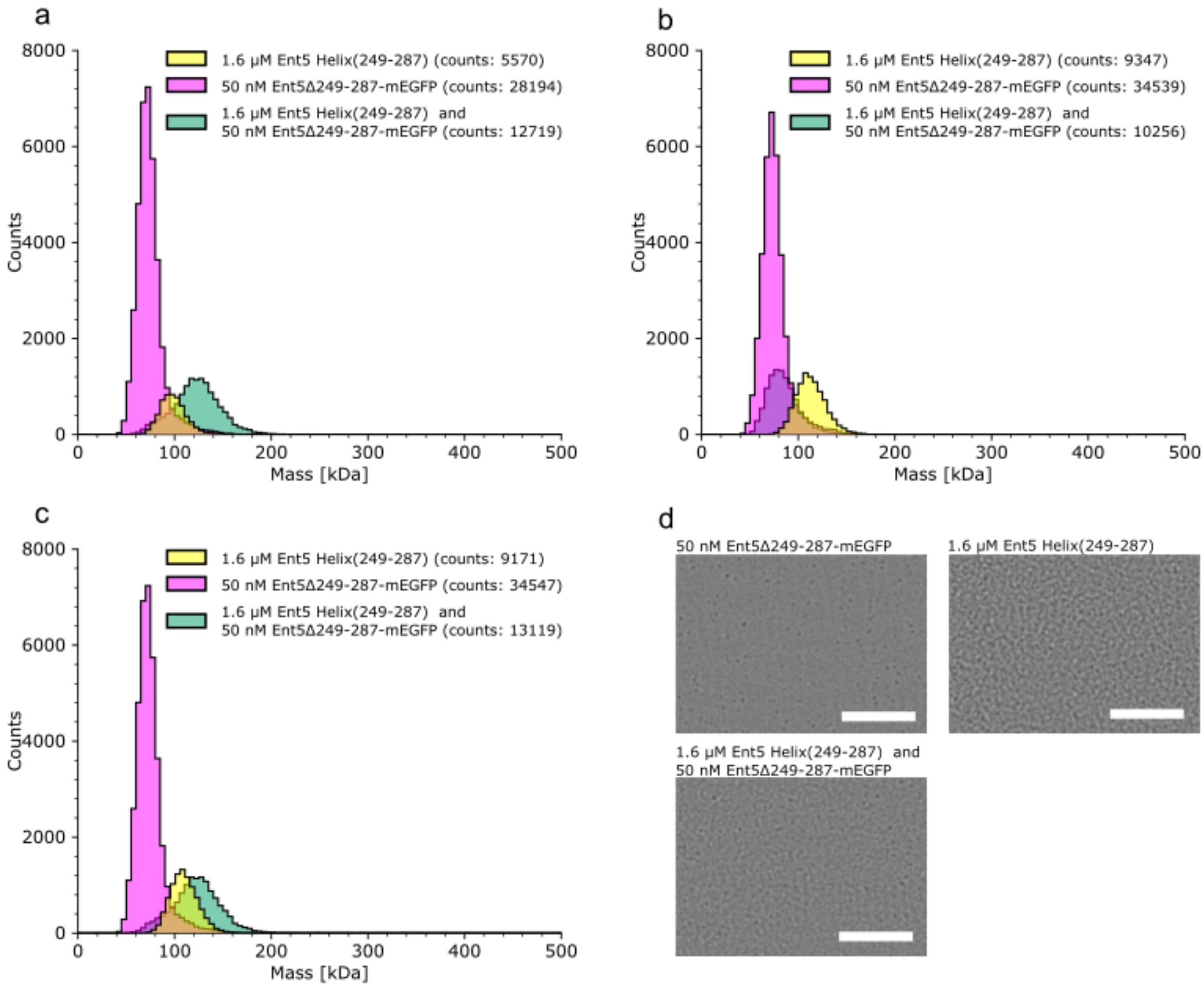

Figure: Mass photometry analysis of Ent5 249-287 peptide and Ent5Δ249–287–mEGFP. Mass distributions generated using the Refeyn analysis software for 1.6 μM Ent5 helix 249-287 peptide (yellow), 50 nM Ent5Δ249–287–mEGFP (magenta), and the corresponding mixture (teal) in three individual measurements (a–c). The number of detected events is indicated in each panel. The peptide-containing samples produced substantial interferometric contrast and background, which limited reliable particle detection and mass assignment in the mixed samples. (d) Representative fields of view acquired for Ent5Δ249–287–mEGFP alone, Ent5 249-287 peptide alone, and the corresponding mixture.

3. GUV assays:

Giant Unilamellar Vesicles (GUVs) typically have diameters of around 10 μm and are sufficiently flat, making them a very good experimental system for mimicking cellular

membranes. The authors have used GUVs as a vehicle for determining the recruitment of both Ent5 and Sla2 condensates to membrane environments (Figure 6). However, membrane environment typically lowers the critical concentration for LLPS by confining diffusion (PMID: 35411085, 32726575, 31604601).
1) In figure 6a-e, are the protein concentrations used by the authors representative of physiological conditions, or at least close to physiological levels in the lipidic condition? Because the protein concentrations used by the authors are not reduced by many orders of magnitude compared with those in solution.

We thank the reviewer for this important comment.

The physiological concentrations of Ent5 and Sla2 at their sites of action are not known with sufficient precision to allow a direct comparison with our in vitro conditions. Therefore, we cannot conclude whether the bulk protein concentrations used in our GUV experiments fully recapitulate physiological levels.

In our original experiments (Fig. 6a–e), we deliberately used bulk protein concentrations that were lower than those employed in our solution-based LLPS assays. Ent5 was used at 3.5 μM in the GUV assays compared with 17 μM in solution (Figs. 2 and 4), whereas Sla2 was used at approximately 1 μM compared with approximately 2.5 μM in the PEG-induced solution assays (Fig. 3a). These concentrations enabled robust visualization of membrane recruitment while avoiding extensive membrane saturation.

Following the reviewer's suggestion, we have now repeated the membrane recruitment experiments using substantially lower Ent5 protein concentrations. As shown in the new Supplementary Fig. S12, robust recruitment of WT NTD–CHC to Ent5-rich membrane condensates is readily observed using only 500 nM Ent5 and 500 nM NTD–CHC, representing a seven-fold reduction in Ent5 concentration compared with the original GUV experiments. These new data further support the conclusion that membrane association enables condensate formation and client recruitment at considerably lower bulk protein concentrations.

This behaviour is consistent with the established principle that the relevant parameter in the membrane context is not the bulk concentration in solution but the local surface density achieved upon membrane binding. Membrane recruitment effectively confines proteins to two dimensions, substantially increasing their local concentration and thereby lowering the apparent saturation concentration (C_{sat}) required for phase separation.

We have included these additional low-concentration experiments as Supplementary Fig. S12 and have clarified this point in the revised manuscript.

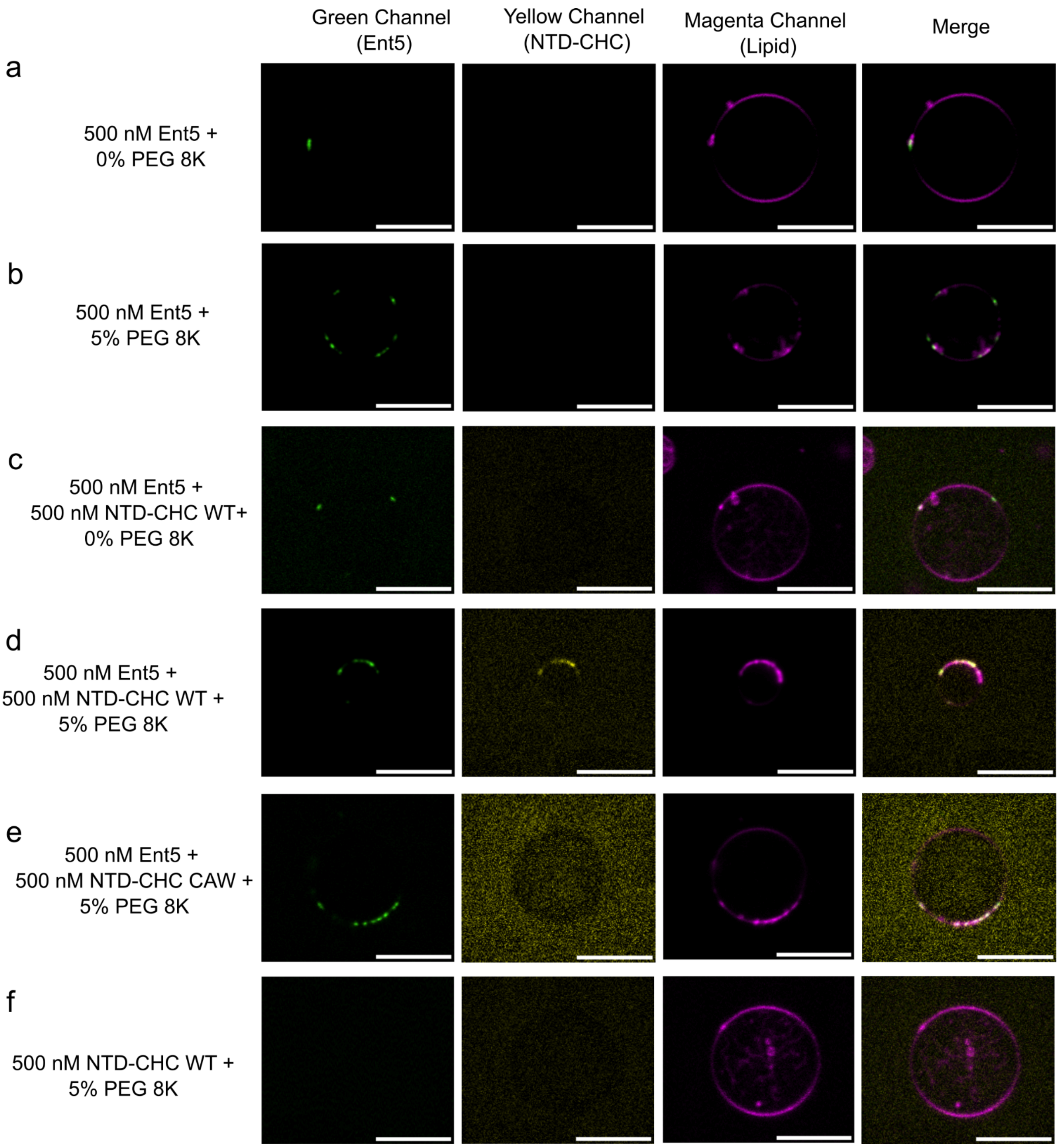

Supplementary Figure 12. NTD–CHC is recruited to Ent5 membrane condensates assembled on PI(3,5)P_2-containing GUVs. Representative spinning-disk confocal images of GUVs in presence of Ent5-mEGFP and NTD-CHC-mScarlet. GUVs were incubated for 5 min at room temperature with Ent5–mEGFP (green) and/or NTD–CHC–mScarlet (yellow) in 100 mM Tris-HCl, pH

7.5, 150 mM NaCl, and 0.5 mM TCEP. The lipid membrane is shown in magenta (DOPE-Cy5.5). (a) 500 nM Ent5–mEGFP without PEG 8000. (b) 500 nM Ent5–mEGFP with 5% (w/v) PEG 8000. (c) 500 nM Ent5–mEGFP and 500 nM WT NTD–CHC–mScarlet without PEG 8000. (d) 500 nM Ent5–mEGFP and 500 nM WT NTD–CHC–mScarlet with 5% (w/v) PEG 8000. (e) 500 nM Ent5–mEGFP and 500 nM NTD–CHC CAW–mScarlet with 5% (w/v) PEG 8000. (f) 500 nM WT NTD–CHC–mScarlet with 5% (w/v) PEG 8000. Under crowding conditions, WT NTD–CHC is recruited to Ent5-rich membrane condensates, whereas the CAW mutant shows no comparable recruitment. Images are representative of three independent experiments. Scale bars, 10 µm.

The following text was added to the manuscript:

“Notably, membrane-associated condensates were also observed at an Ent5 concentration of 500 nM (Supplementary Fig. S12), consistent with membrane recruitment increasing the local surface density of proteins in two dimensions and thereby lowering the apparent concentration threshold for phase separation”

2) Addition of condensates can happen on the surface of GUVs and also occurred in the solution. When membraneless organelles contact membrane-bound organelles, a wetting/prewetting behavior is often observed (PMID: 39385023, 37217523, 33473217). However, this phenomenon is not apparent in Figure 6b. In addition, the GUVs shown do not appear to consist of a single bilayer; instead, multilamellar stacking is observed (magenta channel). Could this be a consequence of the high protein concentration used in the experiment? Moreover, z-stack microscopy experiments should be performed, as they would provide stronger support for the proposed interpretation.

We thank the reviewer for this important comment. We agree that contacts between biomolecular condensates and membranes can give rise to wetting/prewetting behaviour and, in some cases, membrane remodelling, as described in the studies cited by the reviewer. Condensate–membrane interactions can produce a range of morphologies, including partial wetting, membrane deformation, bending, and scission. We have therefore revised the text to avoid implying that our current images demonstrate a defined wetting/prewetting state or a specific membrane-remodelling mechanism.

Importantly, in Fig. 6b, Ent5-mEGFP was not added as preformed bulk condensates. Under our imaging conditions, 3.5 μM Ent5-mEGFP is below the bulk saturation concentration, and we do not detect bulk Ent5 droplets in solution by fluorescence microscopy. We therefore interpret the structures in Fig. 6b as Ent5-rich membrane-associated assemblies on PI(3,5)P_2-containing GUVs, rather than recruitment of pre-existing condensates from solution. This interpretation is consistent with the concept that membrane binding can locally enrich phase-separating proteins and promote surface-associated condensation below the concentration required for bulk phase separation (Pombo-García et al. 2024).

We agree that a clear cap-like wetting morphology is not apparent in Fig. 6b. We have therefore avoided assigning these structures to a classical wetting or prewetting regime. Instead, we now describe them more conservatively as membrane-associated Ent5 assemblies. The absence of a classical wetting morphology does not affect the main conclusion of this experiment, namely that Ent5 is recruited to PI(3,5)P_2-containing GUV membranes and forms protein-enriched membrane-associated assemblies under crowding conditions.

Regarding membrane morphology, we agree that the magenta signal appears complex in some images. However, from single-plane confocal images we cannot determine whether this reflects multilamellar membrane stacking, membrane folds or deformation, adjacent vesicles, or local enrichment of lipid signal. We also cannot exclude that local Ent5 surface density contributes to membrane deformation. Nevertheless, high bulk protein concentration alone is unlikely to explain the observed phenotype, because Ent5Δ249–287-mEGFP was tested at the same protein concentration and PEG condition but did not produce comparable membrane-associated structures (Fig. 6c). Furthermore, Supplementary Fig. S12 shows that membrane-associated Ent5 puncta are already observed at 500 nM Ent5 under crowding conditions, and that WT NTD–CHC is recruited to these Ent5-rich assemblies, whereas NTD–CHC alone and the CAW mutant do not show comparable recruitment. These controls support an Ent5-dependent assembly process rather than a nonspecific effect of high protein concentration.

We agree that z-stack imaging would provide additional information about the three-dimensional organisation of these structures. However, in the current assay the GUVs were freely suspended and not tethered. Under these conditions, vesicles undergo substantial translational and rotational movement during image acquisition. Because z-stacks are acquired sequentially, individual optical sections would not reliably correspond to the same vesicle position and orientation, making 3D

reconstruction unreliable and potentially misleading. Immobilising or tethering GUVs would also not constitute a neutral modification for this experiment, because surface adhesion can alter GUV structure, membrane tension, permeability, pore formation, and even rupture behaviour (Dolder et al. 2022). Since membrane tension and adhesion are directly relevant to condensate–membrane wetting and remodelling, such experiments would require separate optimisation and validation and fall beyond the scope of the present study.

To avoid overinterpretation, we have modified the text and Figure 6 title accordingly:

"Ent5 and Sla2 are recruited to signalling-lipid-containing GUVs and form membrane-associated protein-enriched assemblies under crowding conditions. Because the GUVs were imaged as freely suspended vesicles in single optical planes, these data do not resolve the three-dimensional membrane topology or bilayer number of the observed structures. Thus, while the images support membrane recruitment and membrane-associated assembly, they do not distinguish between membrane folding, vesicle apposition, multilamellar stacking, or other forms of membrane remodelling"

Figure 6. Ent5 and Sla2 form membrane-associated assemblies on GUVs containing signaling lipids. a-c) Preformed GUVs, containing 8% PI(3,5)P_2, were incubated at 21 °C with 3.5 µM Ent5-mEGFP. Fluorescence images were acquired in two channels: the protein is visualized in the green channel (mEGFP) and the lipid in the magenta channel (Cy5.5). Three conditions are shown: a) 0% (w/v) PEG 8000, b) 5% (w/v) PEG 8000 and c) 3.5 µM Ent5 Δ249-287-mEGFP in the presence of 5% (w/v) PEG 8000. d-e) Preformed GUVs containing 8% PI(4,5)P_2 were incubated at 21 °C with a mixture of 1 µM Sla2–mEGFP and 1 µM Ent1-ENTH. Two conditions are displayed: d) 0% (w/v) PEG 8000 and e) 2.5% (w/v) PEG 8000. White scale bars in all images represent 5 µm.

3) Finally, PIP_2 is generally a minor lipid component of the plasma membrane. In eukaryotic cells, its abundance is typically below 2%, and most in vitro reconstitution studies use ≤5% PIP_2 to approximate physiological conditions (PMID:15107463, 33202250, 29606581). In this study, however, 8% PIP_2 was used in the GUV experiments. The authors should justify the choice of this relatively high PIP_2

concentration. Excessive PIP_2 may substantially alter membrane electrostatics, surface potential, and other biophysical properties of the bilayer, potentially leading to non-physiological behavior. It would therefore be important to clarify whether the observed effects depend quantitatively on PIP_2 content, and whether similar results can be obtained at lower, more physiological PIP_2 fractions. Otherwise, there is a concern that the experimental conditions may be overly artificial.

We thank the reviewer for this important comment.

We agree that the average abundance of PIP_2 in the plasma membrane is typically below 2%. However, the lipid composition used in our GUV assays should be viewed as a simplified model system designed to promote phosphoinositide-dependent membrane recruitment rather than as a direct recapitulation of the native membrane environment.

The choice of 8 mol% PIP_2 was not arbitrary. We used the same GUV preparation and lipid composition as in our previous published work investigating ENTH- and ANTH-domain membrane interactions (Lizarrondo et al. 2021), where 8 mol% PI(4,5)P_2 supported robust and reproducible membrane recruitment. Similar phosphoinositide concentrations have also been used in other reconstitution studies of ENTH/ANTH-containing proteins (Skruzny et al. 2015).

We agree that phosphoinositide content influences membrane surface charge and protein recruitment and may therefore shift the quantitative threshold for condensate formation. Accordingly, our results should be interpreted as demonstrating the capacity of membrane recruitment to promote condensate formation under conditions of efficient phosphoinositide-dependent binding, rather than as a quantitative recreation of native membrane composition. We have clarified this point in the revised manuscript.

The following statement was added to the Methods section with the related references:

"The GUV lipid composition was chosen to match conditions previously validated for ENTH/ANTH membrane recruitment and remodelling assays (Lizarrondo et al., 2021 and Skruzni et al, 2015)."

4. Discussion Language:

In line 393, “This helix alone is insufficient to promote condensation, but together with the surrounding disordered region and the ENTH domain, it enables robust LLPS at physiological concentrations.” should be toned down. The authors state that Ent5 undergoes LLPS at physiological concentrations. However, the manuscript does not provide any quantitative information regarding the physiological abundance or concentration of Ent5 in yeast, nor is this discussed with reference to existing literature. Or the authors should therefore explicitly state the physiological abundance/concentration of Ent5 in yeast (with appropriate citation or measurement); discuss how the in vitro concentrations used relate to the estimated in vivo levels; if membrane binding and PIP_2-dependent recruitment are proposed to elevate the local effective concentration of Ent5 to the phase separation regime, this should be quantitatively justified or experimentally supported. Clarifying these points is essential to substantiate the claim that Ent5 undergoes LLPS under physiologically relevant conditions.

We thank the reviewer for this important point. We agree that the original wording overstated the physiological relevance.

Proteome-wide quantification studies estimate Ent5 to be present at approximately 8,100 molecules per cell in yeast during logarithmic growth (Ghaemmaghami et al. 2003) and 42 fL in volume (Jorgensen et al. 2002), corresponding to an average cellular concentration of approximately 0.3 μM. However, this value represents a whole-cell average and does not account for the highly heterogeneous spatial distribution of Ent5, which is recruited to discrete membrane-associated sites within the trafficking machinery.

In vivo, adaptor proteins such as Ent5 are concentrated through membrane binding and multivalent interactions with clathrin and other adaptor proteins. These processes are expected to increase the local effective concentration relative to the bulk cytosolic concentration and may facilitate condensate formation. Consistent with this idea, we now observe membrane-associated Ent5 condensates on GUVs at a bulk protein concentration of 500 nM (Supplementary Fig. S12), substantially lower than that used in our original membrane assays. However, the local concentration of Ent5 at endocytic sites is not known, and our data do not allow us to quantitatively relate the in vitro phase boundary to physiological conditions.

We therefore agree that the original statement was too strong. Rather than claiming that Ent5 undergoes LLPS at physiological concentrations, our data demonstrate that the α-helical element contributes to phase separation under the experimental

conditions examined and that membrane recruitment is likely to promote local protein enrichment.

Accordingly, we have revised the manuscript as follows:

“The peptide containing the 249-287 helix is insufficient to promote condensation, but together with the surrounding disordered region and the ENTH domain, it promotes robust LLPS under the experimental conditions examined. Membrane recruitment is likely to further enhance local protein concentration and facilitate condensate formation.”

Minor Comments

1. The biochemical experiments described in the manuscript employ various full-length proteins and truncated variants. Please provide protein purification data or representative gel images (e.g., SDS-PAGE) to demonstrate protein quality and integrity.

We thank the reviewer for pointing out this omission.

We have now included representative SDS–PAGE gels and size-exclusion chromatography (SEC) profiles for all full-length proteins and truncation variants used in this study in the Supplementary Information. In addition, for constructs displaying minor degradation products, we have included mass spectrometry identification of the corresponding bands.

2. Fluorescent tags can be sufficiently large to influence phase behavior. Indeed, figure S3 indicates that fluorescent tags can promote phase separation. However, to avoid potential artifacts, it would be preferable to also employ small hydrophobic probes to investigate LLPS and confirm that the observed phase behavior is not tag-induced.

We thank the reviewer for raising this important point regarding the potential effects of fluorescent tags on phase behaviour. We agree that fluorescent proteins can, in some cases, influence LLPS and therefore appropriate controls are essential.

Importantly, as shown in Supplementary Fig. S3, untagged Ent5 undergoes phase separation under conditions comparable to those used for Ent5-mEGFP, as observed by DIC microscopy. This demonstrates that LLPS is an intrinsic property of Ent5 and does not require the presence of a fluorescent tag.

In our study, fluorescent tags were primarily used to enable visualization and quantification of protein partitioning and client recruitment. Furthermore, the recruitment behaviour observed is highly specific and dependent on defined molecular interactions rather than generic effects of tagging. For example, the NTD-CHC CAW mutant, which is unable to bind Ent5, does not partition into condensates, and mCherry alone likewise shows no enrichment within droplets. These controls argue against non-specific partitioning driven by the fluorescent moieties.

We agree that the use of small fluorescent dyes could provide additional orthogonal validation. However, given that (i) untagged Ent5 undergoes LLPS as observed by DIC microscopy, and (ii) client recruitment depends on specific protein–protein interactions, we believe the current data provide strong evidence that the observed phase behaviour and selective recruitment are not artifacts of fluorescent tagging.

2. Line 805, “White scale bars (5 µm) in panels b and d indicate the scale for all microscopy images.” Correction: they may be referring to panels d and f.
3. Correction: the Figure 2 contains two panels labeled g, and one of them should be corrected to panel h.

We have corrected both the reference to the scale bars and the panel labels. Below is the modified Figure 2 with its caption.

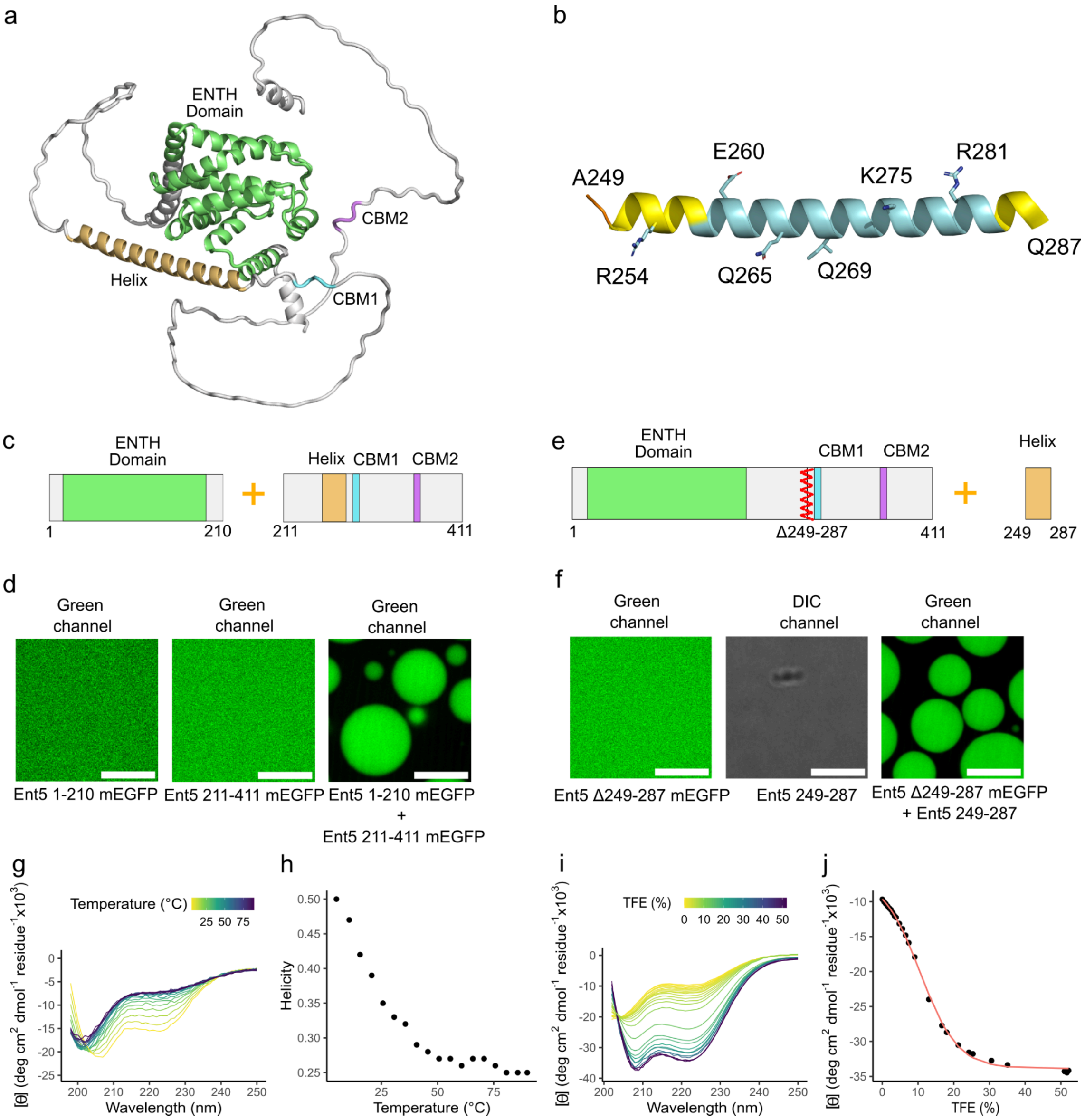

Figure 2. The Ent5 ENTH domain and IDR region containing a predicted helix (residues 249–287) cooperate to promote condensate formation. a) AlphaFold model of Ent5: In green the ENTH domain, the predicted helix in orange, CBM1 in cyan and CBM2 in purple. b) Zoom into the predicted Ent5 249-287 helix. In light blue the model confidence prediction is high (90 > pLDDT > 70) and lower in yellow (70 > pLDDT > 50). c) Constructs used for the complementation experiment: ENTH domain (residues 1-210) and the IDR region (residues 211-411). d) Ent5 ENTH domain (16 μM, left panel) and the IDR region (16 μM, middle panel) in 100 mM Tris (pH 8), 20

mM NaCl, and 0.5 mM TCEP at room temperature (21 °C) do not phase separate. When the two constructs are mixed (both at 16 μM) the sample forms condensates (right panel). e) Constructs used for the complementation experiment: Ent5 Δ249-287 (mutant with a deletion of residues 249-287) and a peptide for residues 249-287 (Ent5 249-287 helix). f) Ent5 ΔHelix (16 μM, left panel) and Ent5 249-287 helix (520 μM, right panel) do not phase separate (same buffer conditions as used in d). When the two constructs are mixed at these concentrations the sample forms condensates (right panel). g) Circular Dichroism (CD) spectra of Ent5 249-287 helix were recorded over a temperature ramp from 5-90 °C. h). The percentage contribution of helical secondary structure,calculated from the molar ellipticity spectra using the Peptide Helicity module of ChiraKit(Burastero et al. 2025), is plotted against temperature. i) Far UV CD spectra for the Ent5 249-287 helix were recorded in the presence of TFE titrated from 0 to 52% (v/v) in 10 mM Tris (pH 7.5). j) Molar ellipticity at 222 nm plotted against TFE percentage. Data were fitted using the Custom Analysis module of ChiraKit (Burastero et al. 2025) with Eq. 2 the following parameters (and their corresponding percentage errors calculated as absolute values): θ_{TFE} = -6470.5 ± 532.7, θ_{water} = -33867 ± 159.5, ΔG_{water} = -1.19 ± 0.07 kcal/mol, m = 11.23 ± 0.44. White scale bars (5 μm) in panels d and f indicate the scale for all microscopy images. Panels d and f are representative of three independent experiments.

4. I suggest labeling Figure 7b directly as Ent5Δhelix, which would improve the visual clarity and readability of the data. Previous studies have reported that Gga2 interacts with Ent5 (PMID: 22457357 and 20704189). In the same context as Figure 7b, it would be important to ask whether the use of the Ent5Δhelix mutant affects the ability of Ent5 to bind Gga2. If so, could the observed prolongation of lifetimes be a consequence of impaired Gga2–Ent5 interaction?

We thank the reviewer for this insightful comment. We have now modified the figure label from "ΔHelix" to "Ent5 Δ249–287" for consistency throughout the manuscript. We agree that the possibility of altered Gga2–Ent5 interaction should be considered when interpreting the observed phenotype.

Previous studies have mapped the Gga2-binding region of Ent5 to residues 327–337 (PMID: 20704189), which is distinct from the helical segment identified in this work (residues 249–287). This spatial separation suggests that deletion of the 249-287 helix is unlikely to directly disrupt the canonical Gga2–Ent5 interaction interface.

Consistent with this, our in vivo data show that recruitment of Ent5 to Gga2-positive structures is not detectably altered in the Δ249–287 mutant, arguing against a major defect in binding. Instead, the primary effect observed is a change in residence time, with Ent5 Δ249–287 exhibiting prolonged retention at these sites.

While we cannot fully exclude indirect effects of the deletion on the interaction, these observations support a model in which the 249-287 helix primarily regulates the dynamics of Ent5 within Gga2-dependent coats rather than its initial recruitment. We have clarified this point in the revised manuscript.

"In addition, recruitment of Ent5 to Gga2-positive structures is not detectably altered in the Ent5 Δ249–287 mutant, indicating that the initial interaction with Gga2 is preserved. Consistent with this, the Gga2-binding region of Ent5 (residues 327–337) is spatially distinct from the deleted helical segment (residues 249–287), suggesting that the Δ249-287 mutation does not directly disrupt the canonical interaction interface."

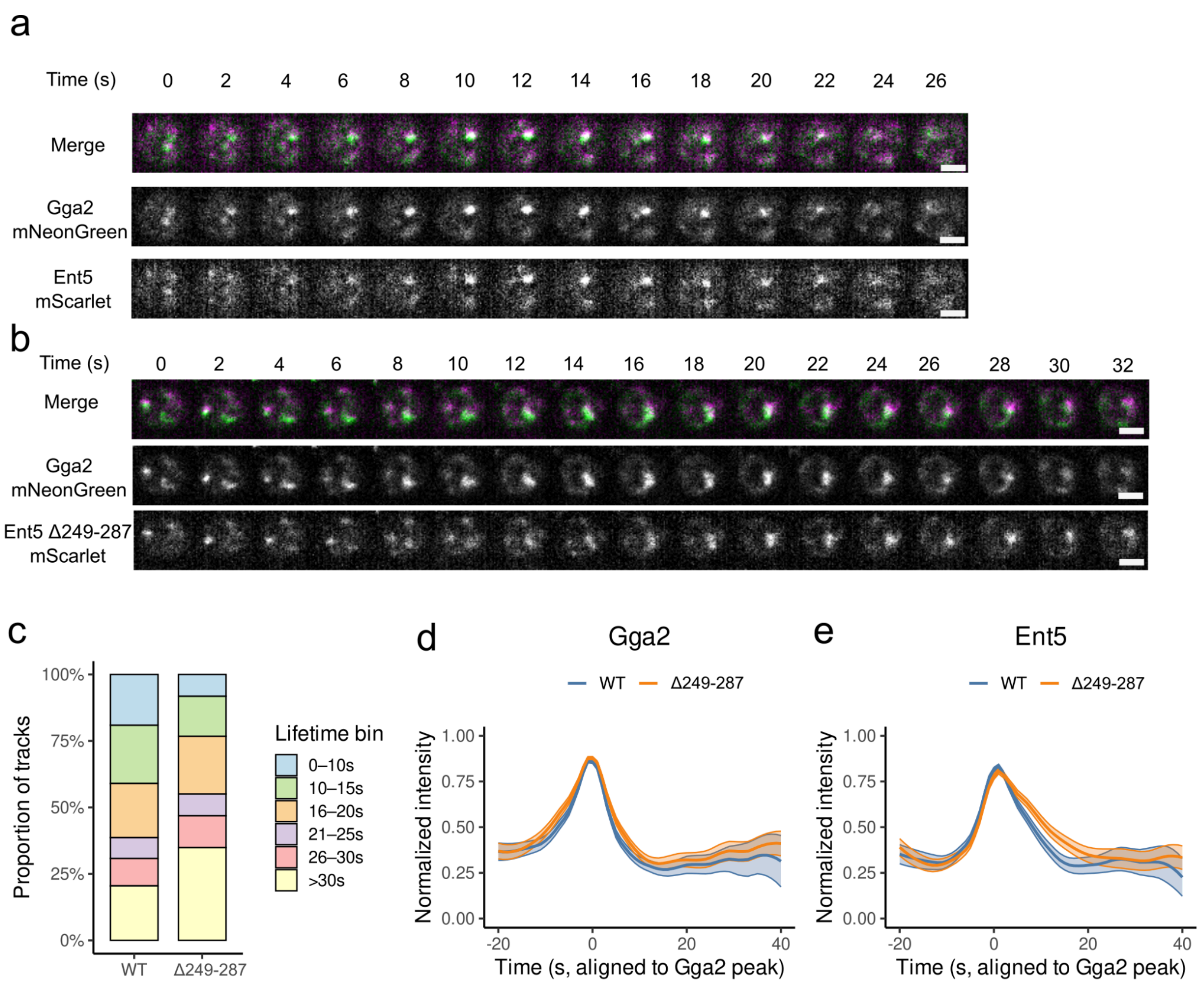

Figure 7. Removal of the Ent5 helix prolongs Gga2-associated coating events in *S. cerevisiae*. a–b) Representative time-lapse montages centered on tracked spots for WT a) and Ent5 Δ249–287. b) Gga2 (Green), Ent5 (Magenta); merged composites shown. Scale bar, 2 µm. c) Proportional stacked bar plots showing the distribution of track lifetimes across six duration bins (0–10, 10–15, 16–20, 21–25, 26–30, >30 s) for WT (left) and Ent5 Δ249–287 (right). Statistical comparison using a χ^2 test of independence on the full contingency table: $\chi^2 = 54.43$, df = 5, $p = 1.71 \times 10^{-10}$. d–e) Mean normalized intensity profiles of Gga2 d) and Ent5 e) for WT (blue) and Ent5 Δ249–287 (orange). Traces are aligned to the Gga2 intensity peak (t = 0), with each curve showing the LOESS fit to the dataset mean (solid line) and 95% confidence interval (shaded region). Individual trajectories were not smoothed prior to averaging. Sample sizes: WT, n = 639; Ent5 Δ249–287, n = 525.

Overall Assessment

This is a strong and carefully executed study that makes a significant contribution to the emerging understanding of LLPS in endocytosis. Addressing the points above-particularly those concerning mechanism and conceptual clarity-would substantially strengthen the manuscript and its impact.

Recommendation:
Minor to moderate revision.

We thank the reviewer for their positive assessment of our work and for their constructive and insightful comments. We have carefully addressed all points raised and believe that the revised manuscript has been substantially strengthened as a result.

Reviewer #2 (Remarks to the Author):

Authors present a study highlighting the role of two distinct clathrin adapters in promoting condensate formation, providing a link between clathrin recruitment to a membrane site and phase separation. The work focuses on Ent5 and Sla2, both of which contain N-terminal phosphoinositide-binding domains and either an intrinsically disordered region (Ent5) or a coiled-coil followed by an actin-binding domain (Sla2). These proteins are used as structure–function examples to support the broader idea that adaptor-driven LLPS underlies clathrin-mediated endocytosis.

The study screened for the role of LLPS using in silico approach and possibly chemical follow ups, but screen data are not shown or extremely challenging for this reviewer to find (??).

We thank the reviewer for pointing this out. We agree that the original manuscript did not sufficiently describe the experimental screening that followed the computational predictions.

Our experimental screen focused on four membrane-associated adaptor proteins (Ent1, Ent3, Ent5, and Sla2) selected from the computational predictions. Other proteins identified by the computational screen were not prioritised for experimental validation. Yap1801 and Yap1802, although predicted to undergo LLPS, function as AP180/CALM-like clathrin adaptors with more specialised roles in endocytosis and

were therefore considered lower-priority candidates than the core endocytic and trafficking adaptors investigated here. Clathrin light chain (CLC) was also not pursued further because its biological function is intrinsically linked to assembly within the clathrin coat, making analysis of its phase-separation behaviour as an isolated component difficult to interpret. Sla1 and Ede1 were not included because their phase-separation properties have already been investigated previously.

We have now incorporated a dedicated Results section describing the experimental screening strategy and outcomes for the epsins Ent1, Ent3, and Ent5, together with the corresponding data in Supplementary Fig. S2. These experiments include turbidity measurements, nanoDSF/static light scattering analyses, fluorescence microscopy, and condition-dependent screening by varying protein concentration, macromolecular crowding, and ionic strength. The results for Sla2 are presented separately in the main figures, where its phase-separation behaviour is characterised in detail.

The new data show that, despite their favourable computational scores, Ent1 and Ent3 did not display behaviour consistent with reversible LLPS under the conditions tested, whereas Ent5 reproducibly underwent LLPS in vitro.

We have revised both the Results and Discussion sections accordingly.

Ent5 and Sla2 were then subjected to standard in vitro LLPS assays, which generally supported the predictions. Interestingly, MP experiments were performed alongside SAXS. These provided some limited evidence for higher MW species.

We thank the reviewer for this comment. We would like to clarify that both mass photometry (MP) and SAXS were employed to investigate the early stages of assembly preceding macroscopic phase separation rather than the condensed phase itself.

Both techniques are well suited to detect the formation of higher-molecular-weight species and nanoclusters in solution but are not appropriate for characterising large micron-sized condensates. In our study, MP and SAXS provided two orthogonal lines of evidence for the formation of higher-order Ent5 assemblies prior to macroscopic LLPS. MP detected the appearance of higher-molecular-weight species, while SAXS revealed the rapid formation and growth of nanoclusters following the reduction in ionic strength.

To characterise macroscopic phase separation, we relied on complementary microscopy-based approaches, including DIC and fluorescence microscopy, which directly visualise condensate formation. Thus, MP and SAXS were not intended as standalone evidence for LLPS, but rather as biophysical methods to probe the initial assembly events that precede condensate formation.

Further experiments tested client/driver hypotheses, ending with a test of the effect of the helix of Ent5 that seemed to drive LLPS. The outcome was limited.

We respectfully disagree with the characterization of these experiments as providing only limited insight. A major objective of this part of the study was to distinguish between LLPS drivers and recruited clients and to determine whether Ent5 condensates selectively enrich biologically relevant interaction partners.

To address this, we first examined the recruitment of the clathrin heavy chain N-terminal domain (NTD-CHC). Fluorescence microscopy experiments demonstrated that NTD-CHC-mScarlet is selectively recruited into Ent5-mEGFP condensates (Fig. 4b). Importantly, recruitment depends on a defined molecular interaction: mutation of the Clathrin-, Arrestin-, and W-box binding sites (CHC CAW mutant) abolishes recruitment (Fig. 4c), consistent with disruption of the interaction between NTD-CHC and the CBM motifs of Ent5. We have further strengthened this conclusion by demonstrating selective recruitment of WT NTD-CHC, but not the CAW mutant, into Ent5 membrane-associated condensates on GUVs (Supplementary Fig. S12). These results demonstrate that client partitioning is not driven by nonspecific incorporation into condensates but instead depends on specific protein–protein interactions.

We next investigated Ent3, an epsin involved in trans-Golgi network trafficking that lacks clathrin-binding motifs. In contrast to NTD-CHC, Ent3 behaves as a recruited client whose concentration increases within the Ent5-rich phase, while Ent3 does not measurably alter the concentration of Ent5 in the condensates (Fig. 4i–j). Furthermore, Ent3 recruitment depends on the concentration of Ent5 (Supplementary Fig. S9a), which is consistent with a client-like behaviour rather than a driving role in condensate formation.

Together, these experiments provide mechanistic evidence that Ent5 acts as a condensate-forming scaffold capable of selectively recruiting interaction partners through defined molecular interactions. We therefore believe that these data substantially strengthen the driver/client model proposed in the manuscript.

Conceptually, the study shows in vitro protein experiments suggestive of LLPS behavior, but without the necessary biochemical controls requisite for most in vitro protein biochemistry work, especially reconstitution. The LLPS field is plagued with multiple tagged constructs, no purification information, and generally biochemistry that is not of acceptable quality to support conclusions drawing on sophisticated physical phenomenon. In this case, I would generally trust that the biochemical reagents are properly assessed (there is a structure even), but it is not shown. Taken as granted, the results are novel and supportive of some data in the literature suggesting the roles of clathrin adaptors linking endocytosis to phase separation. Some molecular data is presented in support of this, and the findings are novel.

We thank the reviewer for pointing out this omission. We have now included representative SDS–PAGE gels and size-exclusion chromatography (SEC) profiles for all full-length proteins and truncation variants used in this study in the Supplementary Information. In addition, for constructs displaying minor degradation products, we have included mass spectrometry identification of the corresponding bands.

Finally, we thank the reviewer for their positive assessment of the novelty of our findings and for recognizing their relevance to the emerging links between clathrin adaptors, endocytic trafficking, and phase separation.

That distinct structures including IDR and CC can take part in LLPS in vitro is undisputed. The finding of "robust LLPS at physiological concentration" doesn't yet apply until we know the in vivo concentration. And there is I think the major problem. The purpose of this behavior is not clearly described nor pursued. So, a phenomenon which is observed in vitro, and supported from a couple orthogonal angles, has a challenging route for impact.

We thank the reviewer for this thoughtful comment. We agree that the original wording overstated the physiological relevance of our in vitro observations and have revised the manuscript accordingly. Throughout the revised text, we no longer claim that either Ent5 or Sla2 undergo LLPS at physiological bulk protein concentrations, as the local concentrations of these adaptor proteins at membrane-associated trafficking sites remain unknown.

The aim of this study was not to establish that LLPS occurs constitutively in vivo, but rather to identify molecular features that enable condensate formation and to investigate their potential functional consequences. For both adaptor proteins, we show that distinct structural elements promote condensation in vitro (the inducible

249-287 α-helix in Ent5 and the coiled-coil-REND region in Sla2). We further demonstrate that membrane association lowers the bulk concentration required for condensate formation, that Ent5 selectively recruits biologically relevant client proteins through specific interactions, and that deletion of the Ent5 249-287 helix alters Gga2 dynamics in vivo.

We have revised the Discussion to better emphasize that these findings provide a mechanistic framework for how phase separation could contribute to the local organisation of endocytic and trafficking machinery. We agree that direct demonstration of the physiological relevance of these condensates in vivo will require future studies.

"While our data establish the molecular determinants and biophysical properties that promote condensate formation in vitro, they do not demonstrate that these assemblies occur as phase-separated condensates in vivo. Rather, our findings provide a mechanistic framework for how membrane recruitment and multivalent interactions could promote local protein enrichment and condensate formation during endocytic and trafficking processes."

The manuscript does not appear ready for publication. The manuscript reads as a thesis-style catalog of proteins and domains rather than a coherent narrative, includes discussion of proteins not pursued experimentally, and is often overly didactic. Some 'lab lingo' used that needs cleanup (e.g. "the helix").

We thank the reviewer for this candid assessment and for highlighting areas where the manuscript could be improved.

We agree that the original version contained sections that were overly descriptive and that some aspects of the narrative could be streamlined. In response, we have revised the manuscript to improve the overall flow and focus on the mechanistic findings.

In addition, we have carefully revised the text to remove colloquial or laboratory-specific terminology and to adopt more precise language throughout. For example, terms such as "the helix" have been replaced by more explicit descriptions of the corresponding 249-287 α-helical region within the Ent5.

Major

The standard for structural protein biochemistry requires the relevant purification details: protein gels, size-exclusion chromatograms, and ideally an independent

measure of the protein size (DLS, native, MP, etc). Size-exclusion chromatography should remove aggregates without reliance on centrifugation; detectable aggregation would indicate poor protein behavior (Methods, Recombinant Protein Purification). Authors should show this information.

We thank the reviewer for pointing out this omission.

We agree that documentation of protein quality and homogeneity is important for the interpretation of in vitro biophysical experiments. We have therefore added representative SDS–PAGE gels and size-exclusion chromatography (SEC) profiles for all full-length proteins and truncation variants used in this study to the Supplementary Information.

We note that several proteins were also characterised using independent biophysical methods, including mass photometry (MP), nDSF and SAXS, which provide information on the folding state, size and oligomeric state of the protein species analysed. These measurements are now more clearly referenced in the revised manuscript.

The centrifugation step following SEC was included as a precautionary measure immediately prior to freezing and storage to remove any particles that may have formed during concentration. It was not intended to compensate for insufficient chromatographic separation. The SEC profiles indicate that the proteins elute as well-defined major peaks, and only fractions corresponding to these peaks were pooled for subsequent experiments.

Mass photometry experiments draw conclusions from n=1 singular insets, despite triplicate measurements. They show very little difference. Technical factors can drive these observations, please refer to https://www.nature.com/articles/s41596-025-01255-4 (unfortunately not field-representative as a single lab published it, but we have replicated many of the findings). Conclusions should be toned down – the reported higher-molecular-weight species appear at extremely low abundance (on the order of ~0.01% of particles, corresponding to only 2–3 particles per cluster), raising questions about their significance. Authors should comment.

We thank the reviewer for this important comment and agree that mass photometry (MP) has limitations when used to quantify low-abundance higher-molecular-weight species.

Our intention was not to use MP as a standalone demonstration of nanocluster formation, but rather as an orthogonal method to probe the presence of higher-order assemblies in solution. We agree that the abundance of these species is low and that caution is required when interpreting rare events detected by MP. Accordingly, we have revised the text to avoid overinterpreting the MP data and to present them as supportive evidence rather than definitive proof of nanocluster formation.

Importantly, the conclusions of the manuscript do not rely solely on MP measurements. The presence of higher-order assemblies is independently supported by SAXS experiments, which reveal the rapid emergence and growth of larger species following the reduction in ionic strength, as well as by stopped-flow light scattering measurements that detect a corresponding increase in scattering intensity. Furthermore, macroscopic phase separation is directly visualised by DIC and fluorescence microscopy.

We therefore view the MP measurements as one component of a broader set of orthogonal observations that collectively support the formation of higher-order Ent5 assemblies. To address the reviewer's concern, we have toned down the corresponding statements in the manuscript and clarified the limitations of the MP analysis.

"In addition, we detected a population of higher-molecular-weight species (>200 kDa) for the wild-type construct specifically under low-salt conditions. Although these assemblies represent only a low-abundance fraction of the total population and should not be overinterpreted quantitatively, their appearance was reproducible and correlated with conditions that promote condensate formation. In contrast, Ent5 Δ249-287 showed reduced signal in this higher-mass range."

We have also revised the figure to include an inset showing the corresponding duplicate measurement and the calculated area. The insets magnify the MWapp region between 200 and 600 kDa. Values above each condition indicate the percentage of the total normalized histogram area within this range.

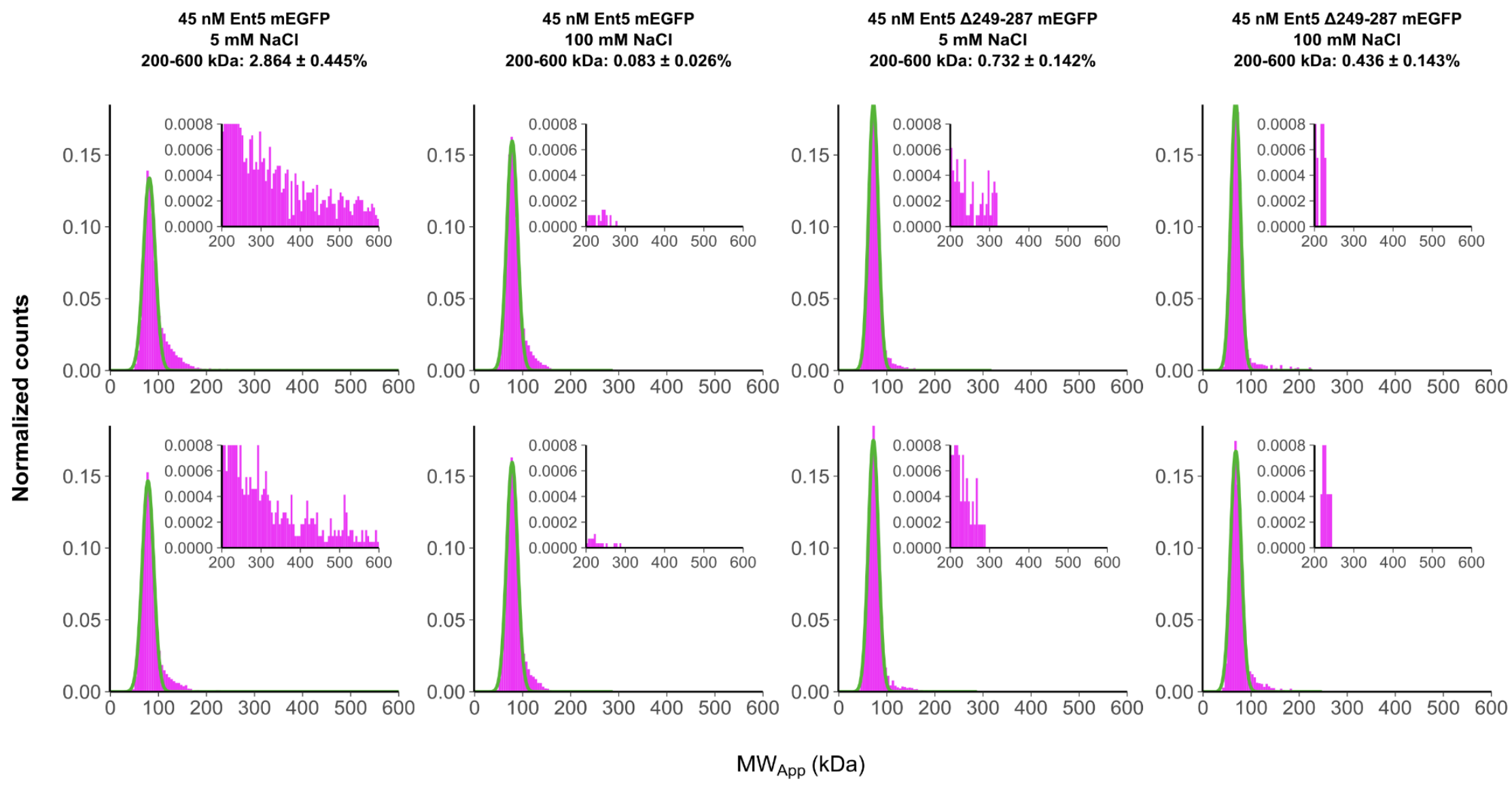

Supplementary Figure 8. Characterization of Ent5-mEGFP nanoclusters by mass photometry. Apparent molecular weight, MW_{app}, histograms obtained from mass photometry measurements of 45 nM Ent5-WT–mEGFP and 45 nM Ent5-Δ249–287–mEGFP in 100 mM Tris, pH 7.5, containing either 5 mM or 100 mM NaCl. Data are shown for n = 2 independent experiments per condition. Normalized histograms are shown in magenta, and fitted Gaussian distributions are shown in green. The expected monomeric molecular masses are 74 kDa for Ent5-WT–mEGFP and 69 kDa for Ent5-Δ249–287–mEGFP. Insets magnify the MW_{app} region between 200 and 600 kDa. Values above each condition indicate the percentage total normalized histogram area within this range, reported as mean ± SD across the two independent experiments. Measurements were performed at room temperature.

Standard LLPS experiments were performed. Purify, incubate, observe droplets, FRAP, domain dissection, mix, conclude LLPS. What is the biological consequence and significance? Do we have concentration, salt, detergent dependence? Crowding agents? The experiments feel somewhat underwhelming in impact. Authors should make clear

We thank the reviewer for this comment. Our intention was to combine complementary biophysical approaches with functional assays to identify endocytic adaptor proteins capable of undergoing LLPS, define the molecular determinants underlying this behaviour, and establish a mechanistic framework for their interactions with membranes and client proteins.

We believe the principal advances of this study lie beyond the observation of LLPS itself. First, we identify Ent5 and Sla2 as condensate-forming adaptor proteins and define the structural elements that drive condensation, namely the inducible α-helical region of Ent5 and the coiled-coil-REND region of Sla2. Second, we demonstrate that Ent5 functions as a condensate-forming scaffold that selectively recruits biologically relevant client proteins through specific molecular interactions, rather than nonspecific partitioning. Third, we show that membrane association promotes condensate formation at substantially lower bulk protein concentrations, supporting a mechanism whereby membrane recruitment facilitates local protein enrichment. Finally, deletion of the Ent5 helix alters Gga2 dynamics in vivo, linking the identified molecular determinant to trafficking behaviour in cells.

Regarding the biophysical characterisation, the revised manuscript now explicitly describes the dependence of condensate formation on protein concentration, ionic strength, and macromolecular crowding for Ent5 (Supplementary Fig. S2), while the effects of crowding and membrane association on Sla2 are presented in the main figures. We note that we did not perform an exhaustive survey of all physicochemical variables, such as detergent dependence, as this was beyond the scope of the present study. We have revised the Discussion to more clearly emphasize the biological implications and to avoid overstating the physiological relevance of the in vitro observations.

Sla2 recruits to Ede1 via “cc-interactions”. What are these? Is coiled coil packing meant, i.e. h-b-c-h-d-e-f? That could be oligomerization, well understood to be a challenge of working with coiled coils at reagent concentrations. Authors need to specifically address what is meant and evidence it more clearly.

We thank the reviewer for pointing out that the term “CC-interactions” was misleading. We have revised the text throughout the manuscript to avoid this terminology. We did not intend to imply a structurally defined coiled-coil packing interface between Sla2 and Ede1, nor that Sla2 recruitment occurs through canonical heptad-repeat-mediated coiled-coil interactions.

Previous work showed that Sla2 is recruited into Ede1 condensates and that regions containing predicted coiled-coil motifs contribute to this process. However, the molecular basis of this recruitment remains unresolved. Our data similarly show that the central coiled-coil-REND region of Sla2 promotes intermolecular association and condensation, suggesting that this region contributes interaction valency. We cannot

distinguish whether these effects arise from canonical coiled-coil packing, self-association, transient surface interactions, or other weak multivalent contacts.

We therefore revised the manuscript to refer to recruitment mediated by coiled-coil-containing regions rather than “CC-interactions”, and we explicitly state that the underlying molecular interface remains unknown. We also agree that isolated coiled-coil domains can oligomerize at elevated protein concentrations, and therefore do not interpret condensation of the isolated Sla2 coiled-coil region as evidence for a specific physiological coiled-coil interaction.

“In Sla2, by contrast, the central coiled-coil-REND containing region appears to make a major contribution to condensate formation. Our truncation and complementation analyses indicate that this region provides interaction valency that promotes condensation, particularly under crowding conditions and in combination with adjacent domains. This observation is consistent with emerging evidence that coiled-coil-containing regions can contribute to LLPS through multivalent intermolecular interactions, rather than functioning solely as static oligomerization motifs (Ramirez et al., 2024; Basalla et al., 2023). Unlike the Ent5 helix region (249-287), the Sla2 coiled-coil-REND containing region is sufficient to form droplets under crowding conditions, and its condensation is further enhanced when combined with neighbouring domains. Together, these findings suggest that endocytic adaptors can employ distinct structural features as determinants of condensate formation. However, our data do not distinguish whether the contribution of the Sla2 coiled-coil-REND-containing region arises from canonical coiled-coil packing, self-association, or other weak multivalent interactions, and the underlying molecular interface remains to be determined.”

The structure is barely connected to the rest of the manuscript. I don't see the point. Multivalency? Avidity? The authors need to rethink how this reads.

We thank the reviewer for this comment. We agree that, in the original manuscript, the structural data were insufficiently integrated into the overall mechanistic framework and their relevance to phase separation was not clearly explained.

We have revised both the text and the discussion of the structural results to better connect them to the LLPS model. Specifically, the structure establishes that Ent5 contains two functional clathrin-binding motifs capable of engaging the N-terminal domain of clathrin through related interaction modes. Together with our biochemical and phase-separation data, this finding supports the view that Ent5 can provide

multiple interaction sites for clathrin engagement. Such multivalency is a central feature of biomolecular condensates and may enhance the recruitment and retention of clathrin within Ent5-containing assemblies.

To improve readability, we now introduce the structural section in the context of multivalent interactions and explicitly discuss how the presence of two functional clathrin-binding motifs may contribute to the interaction network underlying condensate formation. We have also revised the concluding paragraph of this section to better articulate the mechanistic implications of the structural findings.

"Given the central role of multivalent interactions in phase separation, we next examined how Ent5 engages clathrin. Ent5 contains two predicted clathrin-binding motifs (CBM1 and CBM2; Fig. 1a), one of which (CBM1) has previously been shown to bind the N-terminal domain of clathrin heavy chain (NTD-CHC) with low micromolar affinity (Defelipe et al. 2024). To determine whether CBM2 also functions as a clathrin-binding motif, we solved the crystal structure of CBM2 in complex with S. cerevisiae NTD-CHC. The CBM2 peptide engages simultaneously with the three canonical binding sites: Clathrin box, Arrestin box, and W-box (Supplementary Fig. S10; Supplementary Table 4). The overall backbone binding mode is conserved between CBM1 and CBM2, although CBM2 engages NTD-CHC through distinct side-chain interactions. This demonstrates that the overall recognition mode is maintained despite differences in the underlying molecular contacts, highlighting the adaptability of Ent5 clathrin-binding motifs and providing a structural basis for multivalent clathrin engagement. In the Clathrin box site (Asn89), the bulky Trp residue at position +4 cannot occupy the Leu+4 pocket as in CBM1; instead, it stacks against Phe91 through a π–π interaction and forms a hydrogen bond between its indole nitrogen and Asp−1, likely stabilizing its conformation. In the Arrestin box, Trp+4 occupies the same site as Leu+4 in CBM1, enabled by a repositioning of Arg235, which also permits a cation–π interaction between Arg235 and Trp+4. In the W-box site, CBM2 Trp+4 forms an additional hydrogen bond with Gln175, an interaction absent in CBM1, replacing an internal Leu+4–Leu+1–Pro−2 contact. Together, these results establish that Ent5 contains two functional clathrin-binding motifs capable of engaging NTD-CHC through distinct but conserved interaction

modes. The presence of two clathrin-binding sites expands the interaction valency of Ent5 and provides a structural framework for multivalent clathrin engagement. In the context of our LLPS model, such multivalency could facilitate clathrin recruitment and retention within Ent5-containing condensates.

Assumptions from quite old literature have led to the view that certain ENTH domains bind PI4,5P2 and some PI3,5P2. This would be best explicitly tested with modern approaches as in Fig. 6. I don't think this has been systematically done. What if there is no PI35P2? Or PI45P2? These should be explicitly tested and controlled for. The GUV experiments are unfortunately rather haphazard and non-systematic.
Similarly, it is admirable how forthright the authors are on concentrations. However, these appear to vary from place to place, and it is very difficult to conceptualize as currently presented. Authors should not necessarily repeat everything systematically, but consider how the presentation currently looks.

We thank the reviewer for this comment. We agree that a systematic comparison of phosphoinositide specificity would be valuable and could further refine our understanding of ENTH-domain membrane recognition. However, such an analysis falls beyond the scope of the present study.
For the experiments presented here, we focused on $PI(3,5)P_2$ because Ent5 functions in endosomal and Golgi trafficking pathways and, to our knowledge, has not been observed at the plasma membrane. In contrast, $PI(4,5)P_2$ is predominantly associated with the plasma membrane and is best established as a ligand for endocytic adaptors such as Sla2. Indeed, we previously solved the structure of the Sla2 ENTH domain bound to $PI(4,5)P_2$ (Lizarrondo et al. 2021), providing direct structural evidence for this interaction. To better reflect the current literature, we have added a more recent review on phosphoinositides function on membrane trafficking and lipid signaling pathways (De Craene et al. 2017).

Our intention in the GUV experiments was therefore not to establish the phosphoinositide specificity of Ent5, but rather to determine whether recruitment to $PI(3,5)P_2$-containing membranes can promote condensate formation. We chose $PI(3,5)P_2$ because it is more consistent with the known subcellular localization and biological function of Ent5. While we cannot exclude that other phosphoinositides may also support membrane recruitment.
We have clarified this rationale in the revised manuscript and now explicitly state that our conclusions concern condensate formation following $PI(3,5)P_2$-mediated membrane recruitment rather than the phosphoinositide specificity of Ent5.

“In this study, we report that Ent5, an epsin involved in trafficking, and Sla2, a key component of the endocytic mid-coat, both undergo LLPS. We further characterize their roles as drivers and clients. Ent5 consists of an Epsin N-Terminal Homology (ENTH) domain, which has been associated with phosphatidylinositol-3,5-bisphosphate ($PI(3,5)P_2$)-dependent membrane recruitment, followed by an IDR.”

“Regarding proteins involved in intracellular trafficking, Ent3 and Ent5 both contain N-terminal ENTH domains and C-terminal intrinsically disordered regions, similar to Ent1 and Ent2. However, Ent3 and Ent5 function primarily in trans-Golgi network and endosomal trafficking pathways rather than at the plasma membrane. Consistent with their subcellular localization, their recruitment has been linked to $PI(3,5)P_2$-containing membranes. Both Ent3 and Ent5 are predicted to spontaneously phase separate.”

Finally, and in positive spirit, a major challenge remains unresolved: how a ~50 nm coiled coil dimer, exceptionally stable and likely quite rigid, undergoes LLPS. How should one conceive the internal structure of such a droplet? I am not aware of any in vivo or in vitro structural evidence for how these assemblies can be conceived. Moreover, if CC interactions drive LLPS then we would expect turbidity increase with time and irreversible aggregation akin to amyloid/TDP43 aggregation, rather than reversible condensates. Authors should spend time addressing this concept, as it would be central to one of the two adaptors investigated here.

We thank the reviewer for raising this important conceptual point. We agree that the ability of a long, stable coiled-coil dimer to support LLPS requires careful consideration.

We do not envision Sla2 condensates as ordered bundles of coiled coils or amyloid-like assemblies formed through canonical coiled-coil packing interactions. Rather, we favour a model in which the coiled-coil-containing region contributes multivalent interaction capacity and participates in a dynamic associative network. In this view, folded coiled-coil domains can function as interaction modules (“stickers”) connected by flexible regions and engage in weak, reversible intermolecular interactions. This concept is consistent with recent simulations showing that coiled-coil domains can be sufficient to drive LLPS through multivalent inter-chain interactions (Ramirez et al. 2024).

Importantly, stable coiled-coil dimers do not necessarily lead to irreversible aggregation. Coiled-coil-mediated LLPS has been described in several systems, including McdB and Spef1, where folded coiled-coil dimers promote condensate formation while maintaining liquid-like behaviour and reversibility (Basalla et al. 2023; Ren et al. 2026). These studies support the idea that weak interactions between coiled-coil-containing assemblies can generate dynamic condensates without requiring unfolding of the coiled-coil core or formation of ordered fibrils.

Our experimental observations are consistent with such a model. We do not observe progressive time-dependent turbidity indicative of runaway aggregation. Furthermore, Thioflavin T assays performed over seven days and negative-stain electron microscopy do not support the formation of amyloid-like fibrils (Supplementary Fig. S6). We therefore favour a model in which Sla2 condensates consist of a dynamic, physically cross-linked network in which the coiled-coil-containing region contributes multivalent interaction capacity, rather than an irreversible fibrillar aggregate.

We have added the following text to the Results section:
"To further assess whether condensation of the Sla2 IDR-CC construct reflects reversible LLPS rather than progression towards an amyloid-like aggregated state, we monitored Thioflavin T (ThT) fluorescence over seven days using two ThT concentrations in the presence or absence of molecular crowding (Supplementary Fig. S6). In contrast to the amyloid-forming control peptide Temporin-1CEa, Sla2 IDR-CC-mScarlet did not exhibit a time-dependent increase in ThT fluorescence. Consistent with this observation, negative-stain electron microscopy performed after 140 h incubation did not reveal fibrillar or amyloid-like structures (Supplementary Fig. S6e). Together, these results indicate that condensation of the Sla2 coiled-coil-containing region does not progress to detectable amyloid formation under the conditions tested and is consistent with the formation of dynamic condensates rather than irreversible aggregates."

We have also revised the Discussion to clarify this conceptual distinction:

"In Sla2, by contrast, the central coiled-coil-containing and REND regions appear to make a major contribution to condensate formation. Our truncation and complementation analyses indicate that this region provides interaction valency that promotes condensation, particularly under crowding conditions and in combination with adjacent domains. This observation is consistent with emerging evidence that coiled-coil-containing regions can contribute to LLPS through multivalent intermolecular interactions, rather than functioning solely as static oligomerization motifs (Ramirez et al., 2024; Basalla et al., 2023). Unlike the Ent5 helix region (249-287), the Sla2 coiled-coil-containing region is sufficient to form droplets under

crowding conditions, and its condensation is further enhanced when combined with neighbouring domains. Together, these findings suggest that endocytic adaptors can employ distinct structural features as determinants of condensate formation.”

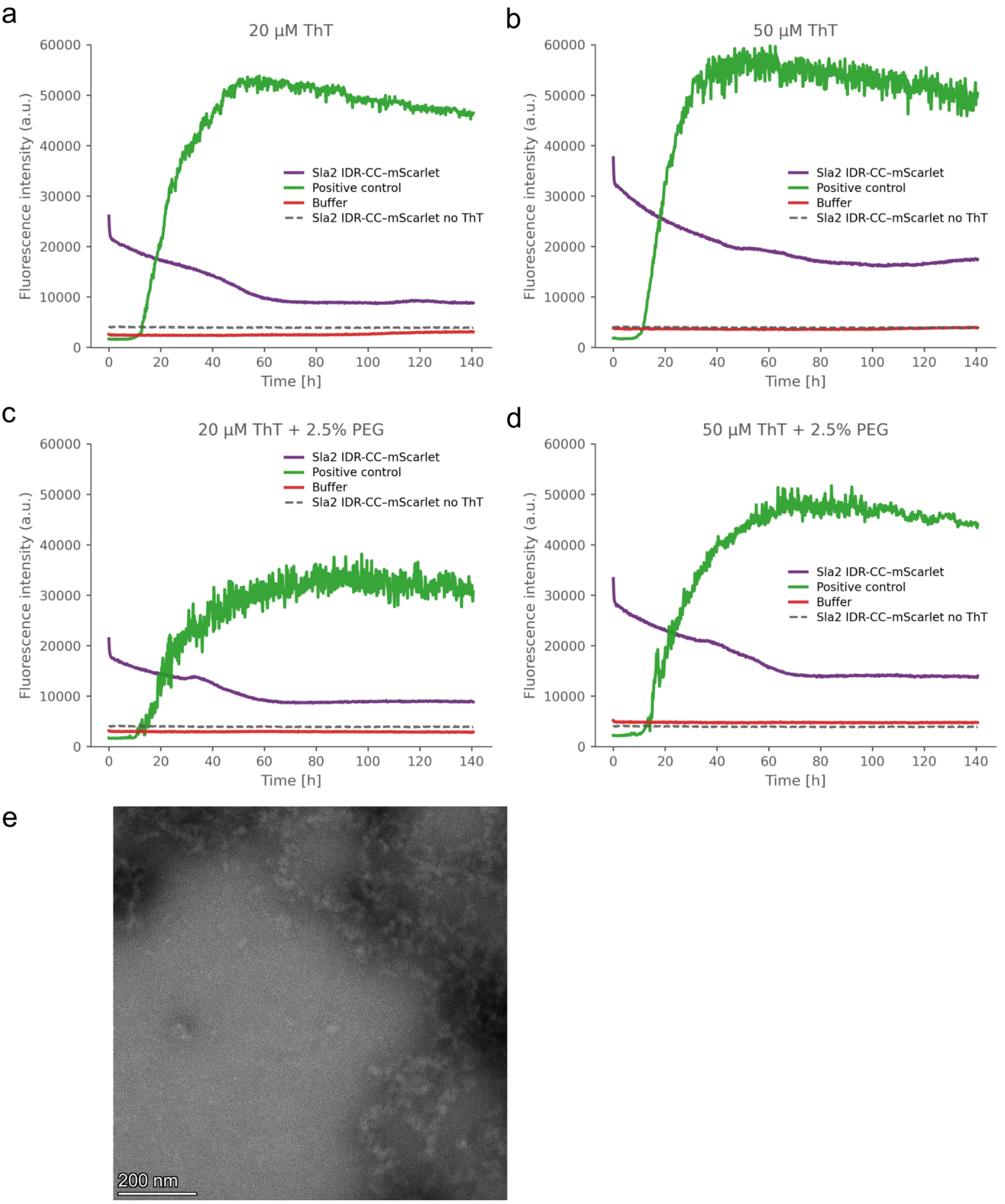

Supplementary Figure 6. Thioflavin T fluorescence and electron microscopy analysis of Sla2 IDR-CC-mScarlet assemblies. Sla2 IDR-CC-mScarlet (violet) and the amyloid-forming positive control Temporin-1CEa (green) were incubated in 30 mM HEPES, pH 8.0, 150 mM NaCl, and 0.5 mM TCEP in the presence of **(a)** 20 µM ThT, **(b)** 50 µM ThT, **(c)** 20 µM ThT plus 2.5% (w/v) PEG 8000, or **(d)** 50 µM ThT plus 2.5% (w/v) PEG 8000. Buffer-only controls (red) and the autofluorescence control of Sla2 IDR-CC-mScarlet in the absence of ThT (grey, dashed) were measured in parallel. Fluorescence was monitored every 10 min for 140 h at 37 °C. Traces represent the mean of three replicates; standard deviations are included but are not visible at this scale. **(e)** Negative-stain electron microscopy micrograph of 20 µM Sla2 IDR-CC-mScarlet after 140 h incubation under the conditions shown in (d). No fibrillar or amyloid-like structures were observed. Scale bar: 200 nm.

Minor

-Should cite the below re: Ent5 binding PI35P2 (current citations 17 Duncan, and 18 Eugster): Chidambaram et al. Journal of Biological Chemistry, Volume 279, Issue 6, 2004, Pages 4175-4179, ISSN 0021-9258, https://doi.org/10.1074/jbc.M308667200

We have added the suggested citation.

-Target selection from the screen is not clear.

This has been already addressed in a previous response and Supplementary Figure 2 has been added to the manuscript.

-Fig 1 should say “in vitro” in the title and in legend should say “droplets existed at” rather than formed, as no concentration dependent experiments were performed.

We have performed the suggested corrections to Figure 1.

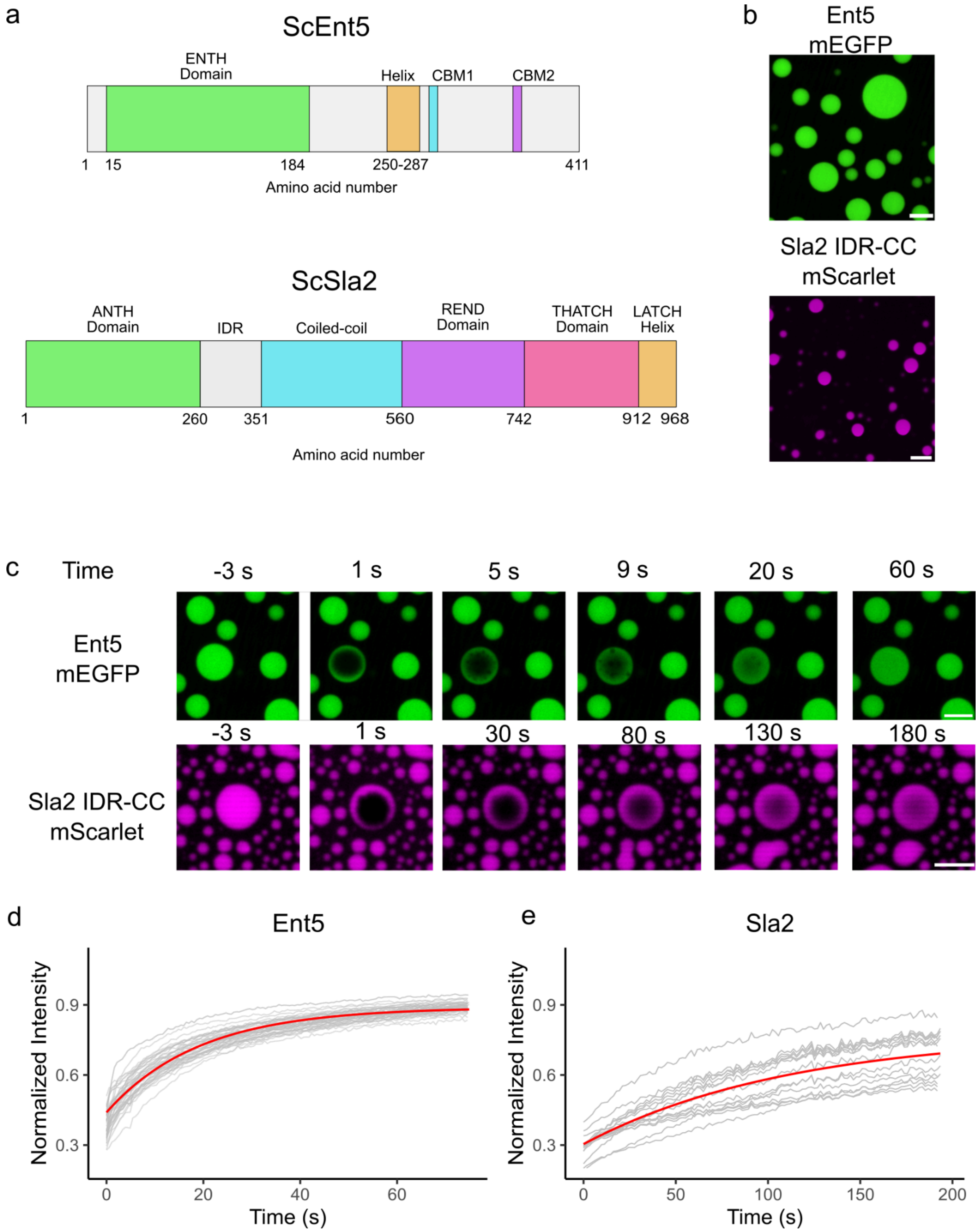

Figure 1. Ent5 and Sla2 form liquid condensates *in vitro*. a) Domain architecture of Ent5 and Sla2 from *Saccharomyces cerevisiae*. Ent5 is formed by a membrane binding ENTH

domain (15-184) and a IDR (211-411), which is composed of a predicted central helix (249-287), Clathrin Binding Motif (CBM) 1 (LIDL, 293-296) and 2 (LLDW, 355-358). Sla2 is composed of a membrane binding ANTH domain (1-260), IDR (260-351), Coiled-coil (CC,351-560), REND domain (560-742), THATCH domain (742-912) and a LATCH helix (912-968). b) Exemplar images of condensates at room temperature (21 °C) for Ent5 full-length N-terminal tagged with mEGFP (upper panel) and a truncated Sla2 (IDR and CC) tagged C-terminally with mScarlet (bottom panel). Ent5 droplets existed at 18 μM protein concentration in buffer (100 mM Tris pH 8, 20 mM NaCl, and 0.5 mM TCEP). Sla2 droplets existed at 25 μM protein concentration in the presence of PEG (2,5 % PEG 8000, 30 mM HEPES pH 8, 150 mM NaCl, 0.5 mM TCEP). The white scale bar represents 5 μm. c) Representative FRAP time course of droplets for Ent5-mEGFP (upper panels) and Sla2 IDR-CC-mScarlet (lower panels). Upper panels show confocal images of an Ent5-mEGFP droplet before photobleaching (–3 s) and at 1, 5, 9, 20, and 60 s after bleaching; lower panels show confocal images of a Sla2 IDR-CC-mScarlet droplet before photobleaching (-3 s) and at 1, 30, 80, 130, and 180 s after bleaching. d-e) Normalized intensity recovery plots for individual droplets are shown in grey, n=45 for Ent5 (panel d) and n=24 for Sla2 (panel e). A simple diffusion model fit is overlaid in red. Ent5 recovered with a time constant of (19.2 ±0.2) s (d). Sla2 recovered with a time constant of (112.2 ± 2.4) s (e).

-Fig 2 has two ‘g’ panels. “The helix” should define precisely the construct used (Ent5249-287, I believe).

We have corrected Figure 2 according to the reviewer’s suggestions

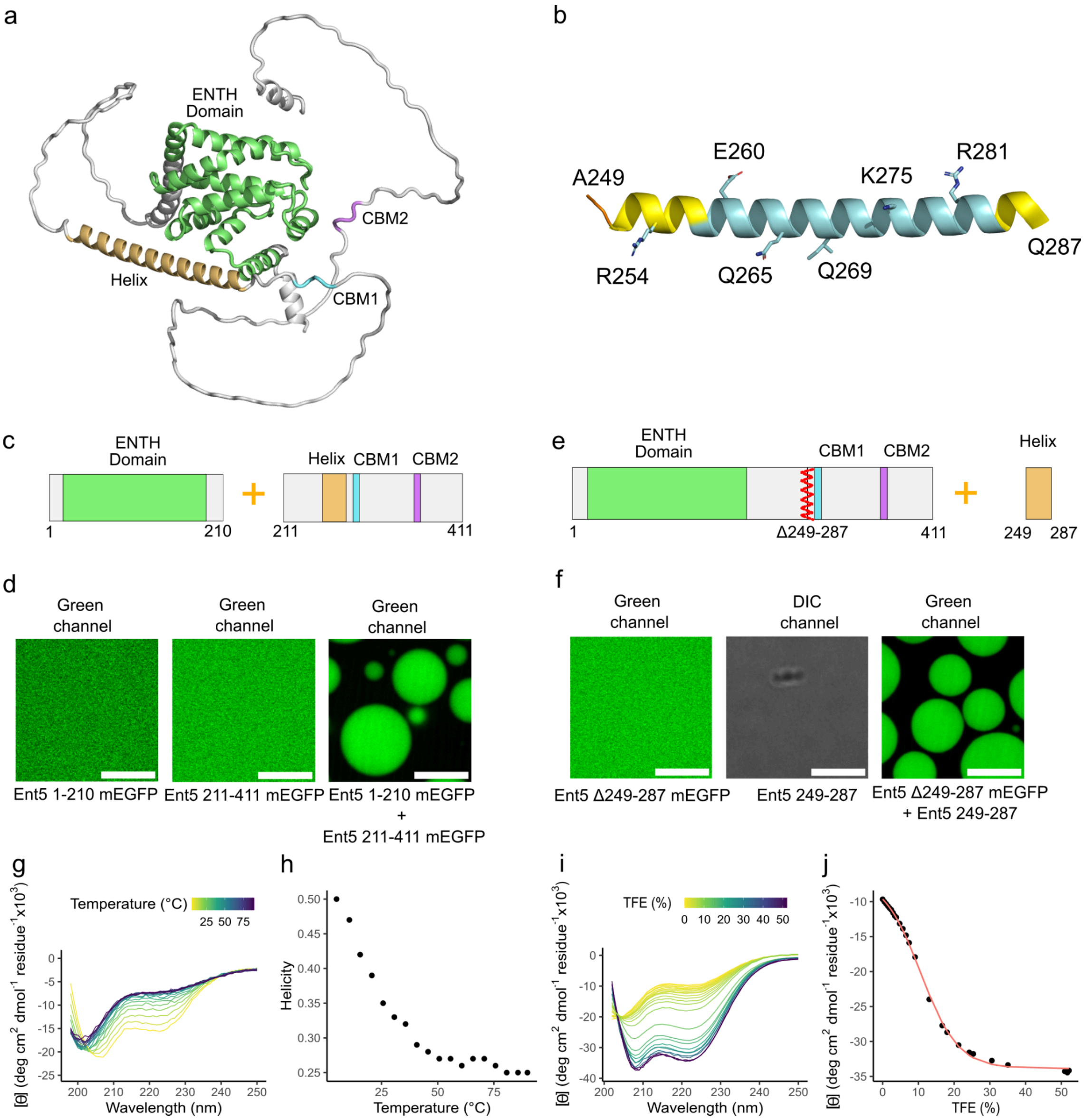

Figure 2. **The Ent5 ENTH domain and IDR region containing a predicted helix (residues 249–287) cooperate to promote condensate formation.** a) AlphaFold model of Ent5: In green the ENTH domain, the predicted helix in orange, CBM1 in cyan and CBM2 in purple. b) Zoom into the predicted Ent5 helix (249-287). In light blue the model confidence prediction is high (90 > pLDDT > 70) and lower in yellow (70 > pLDDT > 50). c) Constructs used for the complementation experiment: ENTH domain (residues 1-210) and the IDR region (residues 211-411). d) Ent5 ENTH domain (16 µM, left panel) and the IDR region (16 µM, middle panel) in 100 mM Tris (pH 8), 20 mM NaCl, and 0.5 mM TCEP at room temperature (21 °C) do not

phase separate. When the two constructs are mixed (both at 16 μM) the sample forms condensates (right panel). e) Constructs used for the complementation experiment: Ent5 ΔHelix (249-287) (mutant with a deletion of residues 249-287) and a peptide for residues 249-287 (Ent5 helix (249-287)). f) Ent5 ΔHelix (16 μM, left panel) and Ent5 helix (249-287) (520 μM, right panel) do not phase separate (same buffer conditions as used in d). When the two constructs are mixed at these concentrations the sample forms condensates (right panel). g) Circular Dichroism (CD) spectra of Ent5 helix (249-287) were recorded over a temperature ramp from 5-90 °C. h). The percentage contribution of helical secondary structure,calculated from the molar ellipticity spectra using the Peptide Helicity module of ChiraKit(Burastero et al. 2025), is plotted against temperature. i) Far UV CD spectra for the Ent5 helix (249-287) were recorded in the presence of TFE titrated from 0 to 52% (v/v) in 10 mM Tris (pH 7.5). j) Molar ellipticity at 222 nm plotted against TFE percentage. Data were fitted using the Custom Analysis module of ChiraKit(Burastero et al. 2025) with Eq. 2 the following parameters (and their corresponding percentual errors calculated as absolute values): θ_{TFE} = -6470.5 ± 532.7, θ_{water} = -33867 ± 159.5, ΔG_{water} = -1.19 ± 0.07 kcal/mol, m = 11.23 ± 0.44. White scale bars (5 μm) in panels d and f indicate the scale for all microscopy images.

-Use of PEG8000 not justified anywhere and alternative crowding not employed.

We thank the reviewer for this comment. PEG8000 was used as a macromolecular crowding agent to approximate the excluded-volume effects of the highly crowded intracellular environment and to facilitate the observation of phase separation under experimentally tractable conditions. Similar concentrations of PEG have been widely employed in in vitro LLPS studies of endocytic and trafficking proteins, as well as in studies of biomolecular condensates more broadly.

Our objective was not to reproduce the precise physicochemical composition of the cytoplasm, but rather to determine whether the proteins and protein regions investigated possess an intrinsic capacity to undergo condensation. We agree that alternative crowding agents could be used and may influence the quantitative thresholds for condensate formation.

Importantly, for Ent5 we also observed condensate formation under low-salt conditions in the absence of PEG8000. These observations indicate that condensation is not uniquely dependent on PEG-mediated crowding, but can also be promoted by modulation of electrostatic interactions. Together with the concentration-dependent droplet formation, FRAP measurements, membrane recruitment experiments, and complementation analyses presented throughout the manuscript, these data support the conclusion that Ent5 possesses an intrinsic capacity to undergo LLPS.

A relevant "**LLPS induction and monitoring**" section was added to the Methods section.

-Fig 3 – what are the statistic here? What is reproducibility of such experiments?

We thank the reviewer for raising this point. The experiment was performed in three independent replicates (n = 3). This information has now been added to the figure legend.

-Structural descriptions page 7 – what is significance?

This has been already addressed in a previous response to the reviewer.

-Pg. 9 data not shown should be shown. Where is the PI45P2 data?

We thank the reviewer for this comment. We agree that the statement "data not shown" was confusing and have removed it from the revised manuscript.

The comment refers to membrane deformation rather than phosphoinositide binding or condensate formation. Previous work from our laboratory (Lizarrondo et al. 2021) and others (Skruzny et al. 2015) demonstrated that the ANTH domain of Sla2 together with epsin ENTH domains can induce membrane remodelling and tubule formation on PI(4,5)P_2-containing GUVs at sufficiently high protein-lipid concentrations. In the original text, we intended to indicate that the protein concentrations used in the present study are below those previously found to induce detectable membrane deformation.

To clarify this point for the reviewer, we provide an example experiment for the ENTH:Sla2:PIP_2 preformed complex at 10 µM ENTH and Sla2-GFP preincubated with 400 µM PI(4,5)P_2 and added to the GUVs in the absence of PEG 8000, where membrane protrusions consistent with the previously reported membrane remodelling phenotype can be observed. While these observations are in agreement with previous reports, we have not included them in the revised manuscript because membrane deformation is beyond the scope of the present study and does not alter the conclusions regarding condensate formation.

To avoid confusion, we have rephrased the manuscript text to simply state that under the conditions used in this work we observed membrane recruitment and condensate formation, but not detectable membrane deformation.

The previous sentence has been now replaced by:

"At the protein concentrations used in this study, Sla2-mEGFP was uniformly recruited to the membrane without detectable membrane remodelling."

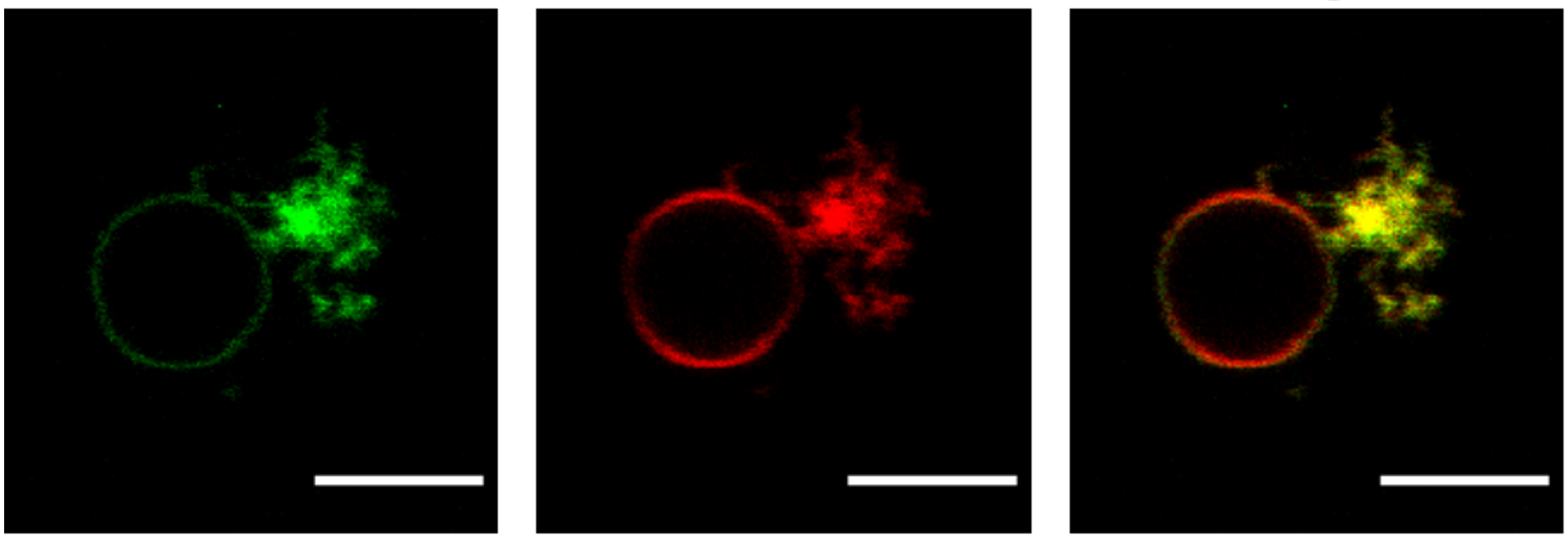

Supplementary Figure for the reviewer: Membrane deformation/tubulation induced by Sla2 and the Ent1 ENTH domain on PI(4,5)P_2-containing GUVs. GUVs were prepared by electroformation using a lipid mixture containing 76 mol% DOPC, 16 mol% DOPS, and 8 mol% 08:0 PI(4,5)P_2 dissolved in chloroform at a final lipid concentration of 1.25 mg ml^{-1}. The lipid mixture was supplemented with 0.1

mol% 14:0 Liss Rhod PE, spread onto two platinum (Pt) electrodes, and dried under vacuum for 1 h. Following electroformation, GUVs were allowed to settle for 30 min and imaged using a Leica SP8 confocal microscope equipped with a white-light laser and a ×63/1.4 NA oil-immersion objective. To assess membrane deformation, full-length Sla2-GFP and the Ent1 ENTH domain were pre-incubated with 400 µM PI(4,5)P_2 and subsequently added to GUVs at final protein concentrations of 10 µM each. Representative images show membrane protrusions and tubule-like structures induced under these conditions. Right, Sla2-GFP fluorescence (green channel); middle, membrane fluorescence from Liss Rhod PE (red channel); left, merged image. Scale bars, 5 µm.

-Simply as a curiousity, have the authors considered Pegylated lipids vs. soluble PEG as crowding agent?

We thank the reviewer for this interesting suggestion. We have not tested PEGylated lipids in this study and instead used soluble PEG 8000 as a conventional macromolecular crowding agent. We agree that PEGylated lipids could provide an interesting alternative strategy to investigate membrane-associated phase separation and will consider this approach in future work.

-Fig 6 no panel f present but referenced in legend

We thank the reviewer for pointing this out. The reference to panel “f” in the Figure 6 legend was incorrect and has now been removed.

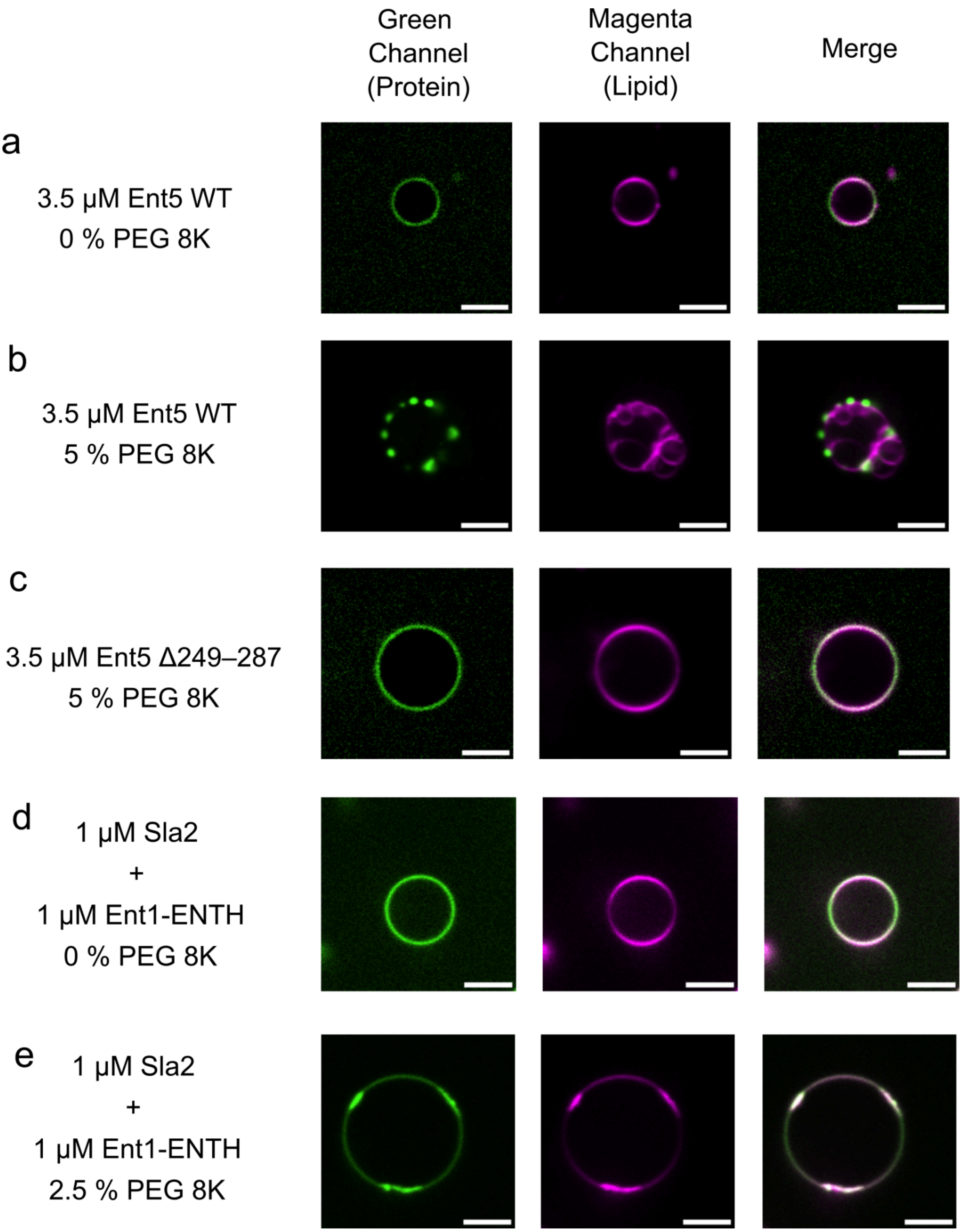
Green Channel (Protein)
Magenta Channel (Lipid)
Merge
a
3.5 μM Ent5 WT
0 % PEG 8K
b
3.5 μM Ent5 WT
5 % PEG 8K
c
3.5 μM Ent5 Δ249–287
5 % PEG 8K
d
1 μM Sla2
+
1 μM Ent1-ENTH
0 % PEG 8K
e
1 μM Sla2
+
1 μM Ent1-ENTH
2.5 % PEG 8K

Figure 6. Ent5 and Sla2 form membrane-associated assemblies on GUVs containing signaling lipids. a-c) Preformed GUVs, containing 8% PI(3,5)P_2, were incubated at 21 °C with 3.5 µM Ent5-mEGFP. Fluorescence images were acquired in two channels: the protein is visualized in the green channel (mEGFP) and the lipid in the magenta channel (Cy5.5). Three conditions are shown: a) 0% (w/v) PEG 8000, b) 5% (w/v) PEG 8000 and c) 3.5 µM Ent5 Δ249-287-mEGFP in the presence of 5% (w/v) PEG 8000. d-e) Preformed GUVs containing 8% PI(4,5)P_2 were incubated at 21 °C with a mixture of 1 µM Sla2–mEGFP and 1 µM Ent1-ENTH. Two conditions are displayed: d) 0% (w/v) PEG 8000 and e) 2.5% (w/v) PEG 8000. White scale bars in all images represent 5 µm.

Reviewer #3 (Remarks to the Author):

This study is based on an in silico screen for clathrin adaptors with predicted IDRs, that might undergo phase separation. Select candidates were tested for their ability to induce LLPS in vitro. Three of these candidates (Ent5 and Sla2 as validated in this study; Ede1 also based on previous observations) were analyzed in more detail. Indeed, isolated Ent5 and Sla2 can undergo phase separation in vitro. In case of Ent5 this property is further shown to require an α-helix that acts as a sticker. Subsequent experiments reveal that Ent5 can recruit NTD-clathrin into droplets, and that Ede1-droplets can recruit Sla2.
Furthermore, the authors demonstrate that Ent5 and Sla2/Ede1-ENTH can form condensates on PI(3,5)P2- and PI(4,5)P2-enriched membranes, respectively. Finally, the impact of Ent5-dependent LLPS was investigated in living yeast, where loss of a crucial α-helix present in Ent5 is shown to delay the release of Gga2-dependent coats.

The study summarizes very interesting findings on the phase separating properties of a variety of clathrin-associated adaptor proteins, and suggests important novel concepts on how membrane binding, adaptor interactions and clathrin recruitment might be coordinated by condensation. However, a few aspects need to be addressed to render the manuscript suitable for publication in Communications Biology.

Major questions and concerns:

1) The authors need to show the purity of all recombinant proteins used for this study.

We have added this information to the Supplementary material.

2) On page 6 the authors claim that ”Ent5 α-Helix showed significantly less signal above 200 kDa”. How has significance been determined? What is the P value?

We thank the reviewer for pointing this out. We agree that the word “significantly” was inappropriate in this context. No statistical test was performed for this statement, and no P value was calculated. Our intention was to describe a qualitative difference in the representative mass photometry distributions, not to imply statistical significance.

We have therefore removed the term “significantly” from the manuscript and toned down the interpretation. Given the low abundance of events above 200 kDa and the sensitivity of mass photometry to technical factors such as background, event density, detection thresholds and analysis parameters, we now describe these observations only qualitatively.

“In addition, we detected a population of higher-molecular-weight species (>200 kDa) for the wild-type construct specifically under low-salt conditions. Although these assemblies represent only a low-abundance fraction of the total population and should not be overinterpreted quantitatively, their appearance was reproducible and correlated with conditions that promote condensate formation. In contrast, Ent5 Δ249-287 showed reduced signal in this higher-mass range.”

We have also revised the figure to include an inset showing the corresponding duplicate measurement and the calculated area. Insets magnify the MW_{app} region between 200 and 600 kDa. Values above each condition indicate the percentage of the total normalized histogram area within this range.

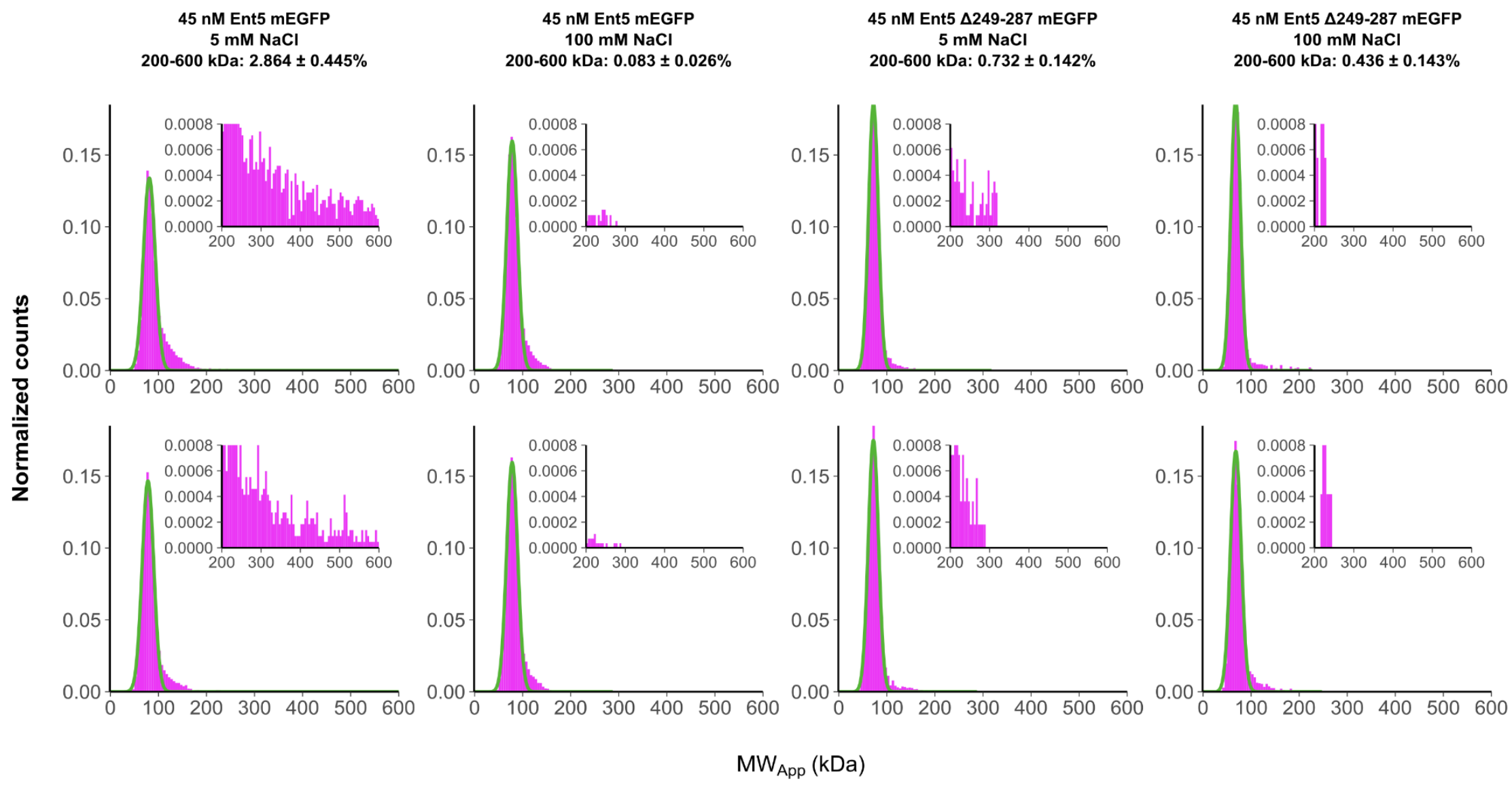

Supplementary Figure 8. Characterization of Ent5-mEGFP nanoclusters by mass photometry. Apparent molecular weight, MW_{app}, histograms obtained from mass photometry measurements of 45 nM Ent5-WT–mEGFP and 45 nM Ent5-Δ249–287–mEGFP in 100 mM Tris, pH 7.5, containing either 5 mM or 100 mM NaCl. Data are shown for n = 2 independent experiments per condition. Normalized histograms are shown in magenta, and fitted Gaussian distributions are shown in green. The expected monomeric molecular masses are 74 kDa for Ent5-WT–mEGFP and 69 kDa for Ent5-Δ249–287–mEGFP. Insets magnify the MW_{app} region between 200 and 600 kDa. Values above each condition indicate the percentage total normalized histogram area within this range, reported as mean ± SD across the two independent experiments. Measurements were performed at room temperature.

3) According to Figs. 1/3 Sla2 IDR-CC forms droplets at 20 µM upon the addition of crowding agent (2.5% PEG 8000). For the data depicted in Figure 5, however, neither results section, nor legend indicate that PEG was added. This difference needs to be explained.

We thank the reviewer for noticing this inconsistency. PEG 8000 was indeed added at 2.5% (w/v) for the experiments shown in Fig. 5, consistent with the conditions used in Figs. 1 and 3. We have corrected the Results section and the Fig. 5 legend to explicitly state the presence and concentration of PEG 8000.

4) Also, I´m confused about the identity of the Sla2 IDR-CC. Based on Fig. 1a/b one would suspect that it covers aa260-560. According to the sketch shown in Fig. 3c/d,

however, the CC additionally contains the REND domain. Have the same protein fragments been used for the two experiments? Then the text needs to be adapted accordingly.

We agree with the reviewer that the nomenclature was confusing. We have addressed this in the revised manuscript by adopting consistent nomenclature and clearly defining each construct in the text.

“To identify the protein regions responsible for LLPS, we designed truncated Sla2 constructs and tested them in vitro (Fig. 3). Each region was expressed with a C-terminal fluorescent tag (either mEGFP or mScarlet). The Sla2 ANTH domain formed condensates at 20–50 μM, while the Sla2 construct containing the IDR, coiled-coil, and REND regions (residues 261–746; hereafter referred to as IDR-CC) formed larger, fluid condensates at a lower threshold (~10 μM). The IDR construct (residues 261–350; hereafter referred to as IDR) and the construct containing the coiled-coil and REND regions (residues 351–746; hereafter referred to as CC) formed condensates in 10% (w/v) PEG 8000 at concentrations of 50 μM and 10 μM, respectively, suggesting that the coiled-coil and REND regions make a major contribution to condensate formation. Our results further indicate that both the IDR-CC and CC constructs form liquid condensates in the presence of 2.5% PEG 8000. These condensates exhibit uniform fluorescence recovery in FRAP experiments, confirming their liquid-like nature (Fig. 3c).”

5) Where relevant the authors need to indicate numbers of experiments/numbers of droplets per experiment underlying their statistics.

We thank the reviewer for this suggestion. We have revised the figure legends, where appropriate, to specify the number of independent experiments and, where relevant, the number of droplets analysed for the corresponding quantitative measurements.

6) In Fig. 5 Ede1 IDR-CC is applied at a concentration of 5 μM, based on a paper that showed condensate formation of the homologous region in human Eps15 (ref. 27). Would 5 μM be sufficient for yeast Ede1 IDR-CC as well? And would Sla2 IDR-CC maybe act as a driver at 5 μM Ede1 IDR-CC?

We thank the reviewer for this insightful comment. We agree that the phase-separation behaviour of the yeast Ede1 IDR-CC construct differs from that

reported for the homologous region of human Eps15. In our hands, purified yeast Ede1 IDR-CC did not undergo detectable phase separation at 5 µM under the assay conditions used in this study. Instead, robust condensate formation was observed only at approximately 45 µM. We have clarified this point in the revised manuscript to avoid implying that the concentration reported for human Eps15 is directly applicable to yeast Ede1.

We also agree that our data reveal a concentration-dependent interplay between Ede1 and Sla2. At 5 µM, Ede1 IDR-CC alone did not form detectable condensates. However, upon addition of 20 µM Sla2 IDR-CC, Ede1 became enriched within Sla2 condensates (ID/IS ≈ 4), demonstrating that Sla2 can recruit Ede1 under conditions where Ede1 alone does not undergo detectable phase separation. In contrast, at 45 µM Ede1, where Ede1 readily forms condensates independently, the partitioning of Sla2 increased substantially, whereas Ede1 exhibited only a small but statistically significant decrease in partitioning ($p = 5.78 \times 10^{-3}$). These observations indicate that the dominant scaffold depends on the relative protein concentrations: Sla2 can recruit Ede1 at low Ede1 concentrations, whereas at higher Ede1 concentrations Ede1 acts as the primary scaffold and promotes the enrichment of Sla2. We have revised the manuscript to clarify this concentration-dependent behaviour.

We have revised manuscript text accordingly:

"Under our experimental conditions, yeast Ede1 IDR-CC did not undergo detectable phase separation at 5 µM, whereas robust condensate formation was observed at approximately 45 µM. Although 5 µM Ede1 alone remained soluble, addition of 20 µM Sla2 IDR-CC resulted in recruitment of Ede1 into Sla2 condensates (ID/IS ≈ 4), indicating that Sla2 can recruit Ede1 under conditions where Ede1 alone does not undergo detectable phase separation. Conversely, Sla2 IDR-CC alone formed condensates at 20 µM with an I_D/I_S ratio of 4.7. When mixed with 45 µM Ede1 IDR-CC, the partitioning of Sla2 increased to an ID/IS ratio of 7.8, whereas Ede1 exhibited only a small but statistically significant decrease in partitioning. Together, these findings suggest that the relative scaffold contribution within mixed condensates is concentration dependent".

For the Discussion section:

"Our findings suggest that the scaffold driving Sla2–Ede1 condensates is concentration dependent. At low Ede1 concentrations, Sla2 can recruit Ede1 into pre-existing condensates, whereas at higher Ede1 concentrations, Ede1 acts as the

EMBL

dominant scaffold and enhances Sla2 partitioning. This behaviour is consistent with the temporal organisation of the yeast endocytic machinery, in which Ede1 is among the earliest proteins recruited to nascent endocytic sites, while Sla2 arrives later during coat assembly (Kozak and Kaksonen 2022; Mund et al. 2018) . Dynamic shifts in the relative abundance of these proteins may therefore contribute to changes in condensate composition and scaffold dominance during endocytic site maturation".

7) Fig.6 shows that Sla2, in concert with Ent1-ENTH, forms condensates in presence of crowding agent, but unlike Ent5 does not deform membranes. Would membrane deformation occur in presence of full-length Ent1? Would phase separation be affected?

We thank the reviewer for this comment. Previous work from our laboratory (Lizarrondo et al. 2021) and others (Skruzny et al. 2015) demonstrated that the Sla2 ANTH domain together with the epsin ENTH domain can induce membrane remodelling and tubule formation on PI(4,5)P_2-containing GUVs at sufficiently high protein-to-lipid ratios. To verify that our experimental system reproduces these published observations, we performed a control experiment using 10 µM Sla2-GFP and 10 µM Ent1 ENTH domain pre-incubated with PI(4,5)P_2 in the absence of PEG 8000. Under these conditions, membrane protrusions consistent with the previously reported membrane-remodelling phenotype were readily observed (see Supplementary Figure for the Reviewer)

In contrast, under the conditions used throughout the present study, which employ lower protein concentrations, we observed membrane recruitment and condensate formation but no detectable membrane deformation. We have therefore rephrased the manuscript to avoid implying that Sla2–Ent1 complexes are unable to remodel membranes, and instead state only our experimental observations under the conditions tested.

We have not investigated the effect of full-length Ent1 on membrane remodelling or condensate formation. As full-length Ent1 is expressed as a GFP fusion in our experiments, we cannot conclude whether it would alter membrane deformation or the phase-separation behaviour compared with the isolated ENTH domain. Addressing this question would require a separate study and is beyond the scope of the present work.

To avoid confusion, we have rephrased the manuscript to state only that, under the conditions used in this work, we observed membrane recruitment and condensate formation:

"At the protein concentrations used in this study, Sla2-mEGFP was uniformly recruited to the membrane without detectable membrane remodelling."

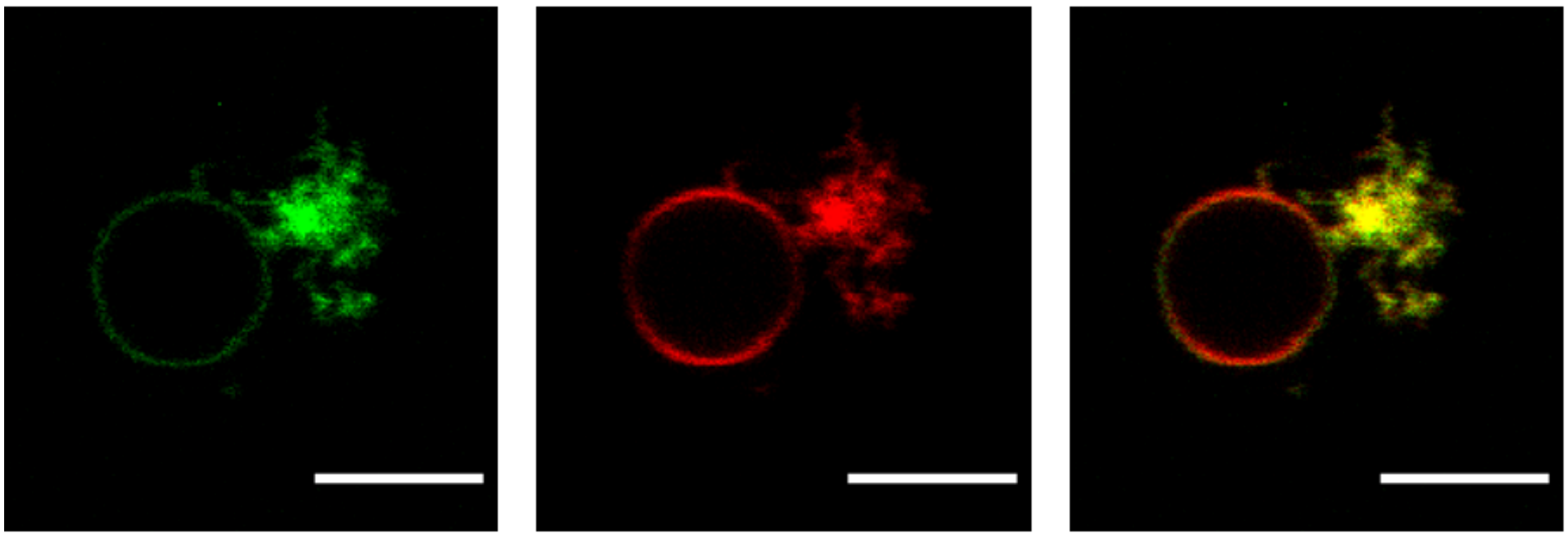

Supplementary Figure for the Reviewer: Membrane deformation/tubulation induced by Sla2 and the Ent1 ENTH domain on PI(4,5)P_2-containing GUVs. GUVs were prepared by electroformation using a lipid mixture containing 76 mol% DOPC, 16 mol% DOPS, and 8 mol% 08:0 PI(4,5)P_2 dissolved in chloroform at a final lipid concentration of 1.25 mg ml^{-1}. The lipid mixture was supplemented with 0.1 mol% 14:0 Liss Rhod PE, spread onto two platinum (Pt) electrodes, and dried under vacuum for 1 h. Following electroformation, GUVs were allowed to settle for 30 min and imaged using a Leica SP8 confocal microscope equipped with a white-light laser and a ×63/1.4 NA oil-immersion objective. To assess membrane deformation, full-length Sla2-GFP and the Ent1 ENTH domain were pre-incubated with 400 μM PI(4,5)P_2 and subsequently added to GUVs at final protein concentrations of 10 μM each. Representative images show membrane protrusions and tubule-like structures induced under these conditions. Right, Sla2-GFP fluorescence (green channel); middle, membrane fluorescence from Liss Rhod PE (red channel); left, merged image. Scale bars, 5 μm.

8) Related to this: The discussion indicates that in contrast to initial computational predictions Ent1, Ent2 or Ent3 did not form condensates in a chemical screen. The data are apparently missing in the manuscript, and also not mentioned in the results section. They should be included.

We thank the reviewer for pointing this out. We agree that the original manuscript did not describe the experimental screening that followed the computational predictions.

To address this, we have now incorporated a dedicated Results section describing the screening strategy and outcomes, together with the corresponding data in Supplementary Fig. S2.

Minor points:

a) On page 7 the authors indicate that “the concentration of Ent5 decreases in the presence of NTD-CHC”. This wording is misleading. Also, the authors should refer to Fig. 4d in the results text, where the relevant quantitative data are depicted. This would greatly assist the reader.

We thank the reviewer for this comment. We agree that the original wording was imprecise and could be interpreted as implying a change in the total protein concentration rather than a change in condensate partitioning. We have therefore revised the text and now explicitly refer the reader to the corresponding quantitative analysis in Fig. 4d.

The sentence has been changed to:

“The partitioning of Ent5 into droplets is reduced in the presence of NTD-CHC, with the I_D/I_S decreasing from 19.24 to 15.06, (Fig. 4d), consistent with Ent5 remaining enriched in the dense phase but partitions less strongly into droplets when NTD-CHC is present. ”

b) Table 1: To my knowledge membrane recruitment of Gga proteins is promoted by PI(4)P, in mammals as in yeast (Daboussie et al., 2016, PMID 22344030). In mammals the GAT domain has been shown to be sufficient for PI(4)P binding and recruitment to liposomes (Wang et al., 2007, PMID 17494868). The respective entries in the table should be corrected.

We thank the reviewer for pointing this out. We have corrected Table 1 to reflect that Gga proteins are recruited to membranes via PI(4)P, in agreement with previous studies in both yeast and mammals.

c) Headline on page 9: contains a spelling error (dependent). Also, the sentence is not really consistent with the actual results: The Ent5 helix rather affects Gga2-dependent coating. Cargo trafficking has not been monitored (though this would be an interesting readout).

We thank the reviewer for this comment. We have corrected the spelling and revised the heading to more accurately reflect the process being measured. Specifically, we now refer to Gga2-dependent coating rather than cargo trafficking, as our experiments monitor coat dynamics rather than trafficking outcomes. The revised title reads:

“**Removal of the Ent5 helix prolongs the lifetime of Gga2-dependent coating**”

d) Text section, as well as legend to fig.7 should indicate the organism the experiment has been conducted with.

We thank the reviewer for this comment. We have revised both the main text and the legend to Fig. 7 to explicitly indicate that the experiments were performed in *Saccharomyces cerevisiae*.

“We found that a dynamic helix (residues 249-287) of Ent5 behaves as a *sticker* for LLPS *in vitro*. To test its functional relevance *in vivo*, we generated a *S. cerevisiae* strain with a construct lacking this helix (ΔHelix) and asked whether its removal alters adaptor-mediated trafficking.”

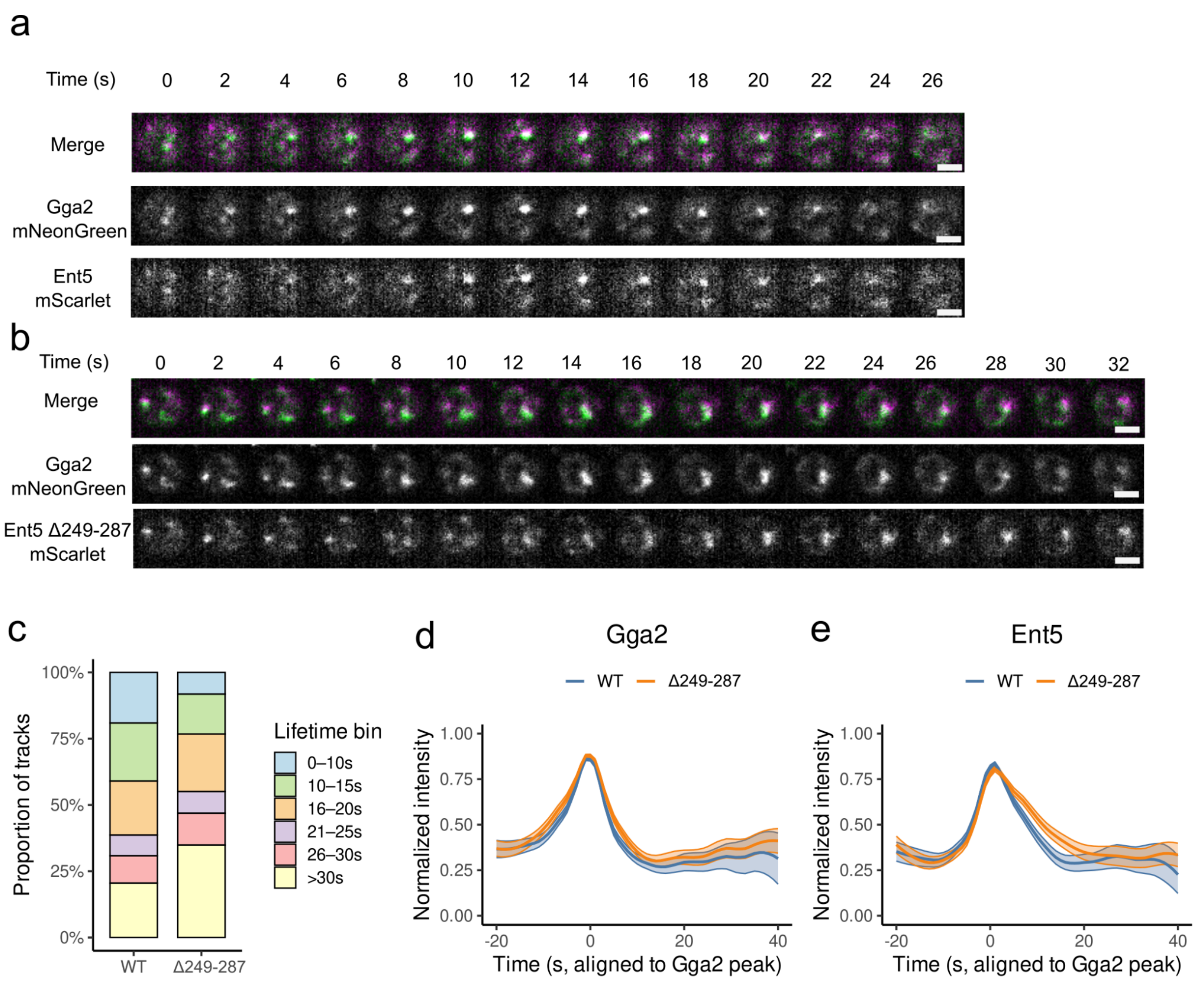

Figure 7. Removal of the Ent5 helix prolongs Gga2-associated coating events in *S. cerevisiae*. a–b) Representative time-lapse montages centered on tracked spots for WT a) and ΔHelix. b) Gga2 (Green), Ent5 (Magenta); merged composites shown. Scale bar, 2 µm. c) Proportional stacked bar plots showing the distribution of track lifetimes across six duration bins (0–10, 10–15, 16–20, 21–25, 26–30, >30 s) for WT (left) and ΔHelix (right). Statistical comparison using a χ^2 test of independence on the full contingency table: $\chi^2 = 54.43$, df = 5, $p = 1.71 \times 10^{-10}$. d–e) Mean normalized intensity profiles of Gga2 d) and Ent5 e) for WT (blue) and ΔHelix (orange). Traces are aligned to the Gga2 intensity peak (t = 0), with each curve showing the LOESS fit to the dataset mean (solid line) and 95% confidence interval (shaded region). Individual trajectories were not smoothed prior to averaging. Sample sizes: WT, n = 639; ΔHelix, n = 525.

e) Page 10, end of results section: the presence of the Ent5 helix affects release dynamics from Gga2-positive structures, rather than its recruitment dynamics to these structures.
We agree with the reviewer and thank them for this suggestion. We have revised the text to clarify that the Ent5 helix affects release dynamics rather than recruitment to Gga2-positive structures.

The sentence has been changed to:

"Together, these results indicate that removal of the Ent5 249-287 helix selectively extends the duration of Gga2-marked trafficking events and shifts the population toward longer-lived assemblies. While Gga2 dynamics remain largely unchanged, the data are consistent with impaired release of Ent5 from Gga2-positive structures rather than altered recruitment."

Remark:
The significance of this manuscript is diminished by a lack of coherence between the different experiments. For instance, the authors demonstrate that Ent5 forms liquid condensates (Figs. 1,2), and drives NTD-CHC or Ent3 recruitment into Ent5-droplets (Fig.4). But it remains unclear if NTD-CHC or Ent3 would also be recruited to Ent5 condensates on PI(3,5)P2-enriched GUVs (Fig.6). On the other hand, the live imaging approach investigates Ent5 recruitment to Gga2-positive Golgi structures (Fig.7). But these structures are most likely enriched in PI(4)P, and it is not further assessed if (membrane-associated) Ent5 could drive Gga2 into condensates (or vice versa: according to Table 1 Gga2 is predicted to undergo phase separation itself).
Similarly, it is shown that Sla2/Ent1-ENTH forms condensates on PI(4,5)P2-enriched GUVs in presence of crowding agents (Fig.6). But it is not investigated, if and how this process would be modulated if Ede1 or clathrin would be added, which are shown to act as drivers or clients of Sla2 condensation, respectively (Fig.5).
The study would greatly benefit if such inconsistencies could be resolved, and maybe reach an even wider field.

We thank the reviewer for this thoughtful suggestion. We agree that linking the solution-based reconstitution experiments with the membrane reconstitution assays strengthens the overall coherence of the study.

Following the reviewer's recommendation, we performed an additional membrane reconstitution experiment to test whether the client identified in solution, NTD–CHC, is also recruited to Ent5 condensates assembled on PI(3,5)P_2-containing GUVs. As shown in the new Supplementary Figure S12, 500 nM Ent5-mEGFP forms

membrane-associated condensates in the presence of 5% PEG 8000 that efficiently recruit WT NTD–CHC-mScarlet. In contrast, the CAW mutant, which is defective in Ent5 binding, fails to show comparable recruitment, and NTD–CHC alone does not associate with the membrane under the same conditions. These results demonstrate that the Ent5-mediated client recruitment observed in solution is preserved in membrane-associated condensates.

We have included these data as Supplementary Figure S12 and refer to them in the Results section.

Regarding the reviewer's additional suggestions to investigate Gga2 recruitment on Golgi-like membranes or the effects of Ede1 and clathrin on Sla2/Ent1 condensates assembled on GUVs, we agree that these are interesting directions. However, addressing all of these combinations would substantially expand the scope of the present work. In particular, the proposed Gga2 membrane reconstitution experiments would require purification of full-length Gga2, which was beyond the scope of the current study. Likewise, membrane reconstitution experiments with full-length Ent1 are technically challenging because the protein exhibits a strong tendency to aggregate under the conditions required for these assays. For this reason, we employed the well-characterised ENTH domain to investigate Sla2 membrane recruitment in a controlled and reproducible manner. Our goal here was to establish the intrinsic phase-separation properties of the core adaptors and demonstrate that the client recruitment observed in solution is also maintained in a membrane context. We believe that the new experiment substantially improves the coherence between the different experimental systems while keeping the manuscript focused.

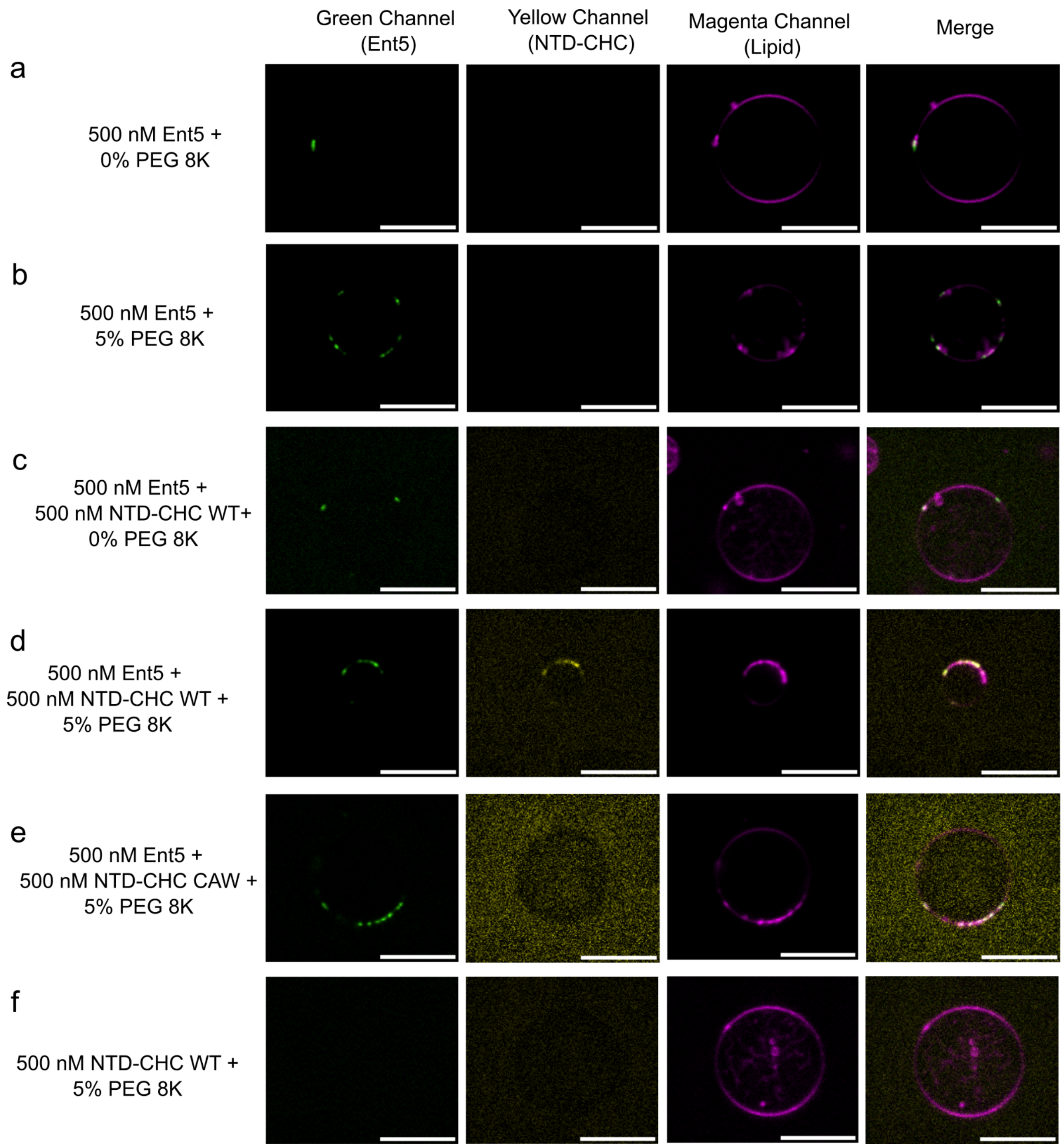

Supplementary Figure 12. NTD–CHC is recruited to Ent5 membrane condensates assembled on PI(3,5)P_2-containing GUVs. Representative spinning-disk confocal images of GUVs in presence of Ent5-mEGFP and NTD-CHC-mScarlet. GUVs were incubated for 5 min at room temperature with Ent5–mEGFP (green) and/or NTD–CHC–mScarlet (yellow) in 100 mM Tris-HCl, pH 7.5, 150 mM NaCl, and 0.5 mM TCEP. The lipid membrane is shown in magenta (DOPE-Cy5.5). (a) 500 nM Ent5–mEGFP without PEG 8000. (b) 500 nM

Ent5–mEGFP with 5% (w/v) PEG 8000. (c) 500 nM Ent5–mEGFP and 500 nM WT NTD–CHC–mScarlet without PEG 8000. (d) 500 nM Ent5–mEGFP and 500 nM WT NTD–CHC–mScarlet with 5% (w/v) PEG 8000. (e) 500 nM Ent5–mEGFP and 500 nM NTD–CHC CAW–mScarlet with 5% (w/v) PEG 8000. (f) 500 nM WT NTD–CHC–mScarlet with 5% (w/v) PEG 8000. Under crowding conditions, WT NTD–CHC is recruited to Ent5-rich membrane condensates, whereas the CAW mutant shows no comparable recruitment. Images are representative of three independent experiments. Scale bars, 10 μm.

References.

Basalla, Joseph L., Claudia A. Mak, Jordan A. Byrne, Maria Ghalmi, Y. Hoang, and Anthony G. Vecchiarelli. 2023. "Dissecting the Phase Separation and Oligomerization Activities of the Carboxysome Positioning Protein McdB." *eLife* 12 (e81362): e81362.

Burastero, Osvaldo, Nykola C. Jones, Lucas A. Defelipe, et al. 2025. "ChiraKit: An Online Tool for the Analysis of Circular Dichroism Spectroscopy Data." *Nucleic Acids Research* 53 (W1): W158–W168.

De Craene, Johan-Owen, Dimitri L. Bertazzi, Séverine Bär, and Sylvie Friant. 2017. "Phosphoinositides, Major Actors in Membrane Trafficking and Lipid Signaling Pathways." *International Journal of Molecular Sciences* 18 (3). https://doi.org/10.3390/ijms18030634.

Defelipe, Lucas A., Katharina Veith, Osvaldo Burastero, et al. 2024. "Subtleties in Clathrin Heavy Chain Binding Boxes Provide Selectivity among Adaptor Proteins of Budding Yeast." *Nature Communications* 15 (1): 9655.

Dolder, Nicolas, Philipp Müller, and Christoph von Ballmoos. 2022. "Experimental Platform for the Functional Investigation of Membrane Proteins in Giant Unilamellar Vesicles." *Soft Matter* 18 (31): 5877–5893.

Ghaemmaghami, Sina, Won-Ki Huh, Kiowa Bower, et al. 2003. "Global Analysis of Protein Expression in Yeast." *Nature* 425 (6959): 737–741.

Jorgensen, Paul, Joy L. Nishikawa, Bobby-Joe Breitkreutz, and Mike Tyers. 2002. "Systematic Identification of Pathways That Couple Cell Growth and Division in Yeast." *Science* 297 (5580): 395–400.

Kozak, Mateusz, and Marko Kaksonen. 2022. "Condensation of Ede1 Promotes the Initiation of Endocytosis." *eLife* 11 (April). https://doi.org/10.7554/eLife.72865.

Lizarrondo, Javier, David P. Klebl, Stephan Niebling, et al. 2021. "Structure of the Endocytic Adaptor Complex Reveals the Basis for Efficient Membrane Anchoring

during Clathrin-Mediated Endocytosis." *Nature Communications* 12 (1): 2889.

Mund, Markus, Johannes Albertus van der Beek, Joran Deschamps, et al. 2018. "Systematic Nanoscale Analysis of Endocytosis Links Efficient Vesicle Formation to Patterned Actin Nucleation." *Cell* 174 (4): 884–896.e17.

Pombo-García, Karina, Omar Adame-Arana, Cecilie Martin-Lemaitre, Frank Jülicher, and Alf Honigmann. 2024. "Membrane Prewetting by Condensates Promotes Tight-Junction Belt Formation." *Nature* 632 (8025): 647–655.

Ramirez, Dominique A., Loren E. Hough, and Michael R. Shirts. 2024. "Coiled-Coil Domains Are Sufficient to Drive Liquid-Liquid Phase Separation in Protein Models." *Biophysical Journal* 123 (6): 703–717.

Ren, Jinqi, Juyuan Liu, Kewei Gu, et al. 2026. "Coiled-Coil-Mediated Phase Separation of Spef1 for Central-Pair Microtubule Organization and Function." *Structure (London, England: 1993)* 34 (5): 718–731.e6.

Skruzny, Michal, Ambroise Desfosses, Simone Prinz, et al. 2015. "An Organized Co-Assembly of Clathrin Adaptors Is Essential for Endocytosis." *Developmental Cell* 33 (2): 150–162.

Our responses in blue.

Reviewer #1 (Remarks to the Author):

I appreciate the authors' thorough and thoughtful responses to my previous comments. Overall, the revised manuscript has improved substantially in clarity, terminology, experimental documentation, and mechanistic framing. Most of my concerns have been addressed satisfactorily. However, I still have a few remaining comments:

1.The new phase-diagram measurements and the lower-concentration GUV experiments are helpful additions. They provide further support for the effects of protein concentration, crowding, and membrane recruitment on Ent5 condensation. The authors have also been more careful in discussing the limitations of the GUV experiments and have toned down their conclusions about physiological relevance, membrane topology, and membrane remodelling.

We thank the reviewer for this positive assessment and for acknowledging that the new phase-diagram measurements and lower-concentration GUV experiments strengthen the manuscript, as well as the revisions made to clarify the limitations and interpretation of these experiments. No further changes were made in response to this comment.

2. I also find the revised discussion of the Ent5 249-287 region more balanced. The data are consistent with this region acting as a sticker through intermolecular interactions, while the exact molecular contacts remain unknown. The additional protein-quality controls, methodological details, replicate information, and revised analysis of the Gga2-associated events have further improved the manuscript.

We thank the reviewer for this positive assessment and for acknowledging that the revised discussion of the Ent5 249–287 region, the additional protein-quality controls and methodological details, and the revised analysis of the Gga2-associated events have improved the manuscript. No further changes were made in response to this comment.

3. I appreciate the authors' attempt to examine peptide-induced oligomerization by mass photometry. I do not think this experiment can be interpreted conclusively. The expected molecular mass of the isolated Ent5(249-287) peptide is only about 4-5 kDa, whereas the peptide-only sample gives a strong signal at around 90-100 kDa. This could reflect peptide aggregation, contamination, measurement background, or an effect of the peptide concentration used. The peptide-protein mixture appears to shift toward approximately 120-130 kDa, but this increase is much larger than expected from the binding of a single peptide and therefore does not demonstrate the formation of a defined peptide-protein complex. The different sample concentrations and total event counts also make direct comparison of the distributions difficult. I don't know what happens using the helix peptide. But I am curious as to why this result appears inconsistent with the LLPS assay, in which addition of the helix peptide was sufficient to restore phase separation of Ent5Δ249-287. One possible explanation is that the helix acts as a sticker only when its local concentration increases within the condensed phase, whereas the corresponding interactions are too weak or transient to be detected in the aqueous dilute phase. It would be helpful if the authors could comment on this possibility.
I do not think that another experiment is essential for publication, as long as the mass photometry result is clearly described as inconclusive and is not used to support a specific oligomeric state or binding stoichiometry. If peptide-induced oligomerization remains an important part of the proposed mechanism, it should be confirmed using an independent and appropriately controlled method. Otherwise, the wording throughout the manuscript should be toned down to make clear that peptide-

induced oligomerization remains a possible explanation rather than a conclusion directly supported by the current data.

We thank the reviewer for this careful assessment and agree that the mass photometry experiment does not provide conclusive evidence for a defined peptide-induced oligomeric state or binding stoichiometry. As noted in our previous response, the isolated Ent5 249–287 peptide generated substantial interferometric contrast/background under the conditions used, which prevented reliable interpretation of the peptide–protein mixture. Importantly, similar signals in the mass photometer can also arise from certain buffer components or additives. The origin of the signal observed for the peptide cannot be established from the mass photometry experiment itself and could reflect peptide aggregation, peptide-dependent effects on the measurement, or assay background. We therefore do not interpret the apparent shift in the mixed sample as evidence for the formation of a defined peptide–protein complex. These mass photometry data were provided only in the rebuttal for completeness and have not been included in the revised manuscript.

We nevertheless appreciate the reviewer's point regarding how the peptide-complementation experiment should be interpreted. We have revised the manuscript to clarify that the ability of the Ent5 249–287 peptide to restore condensation of Ent5Δ249–287 is consistent with the helix contributing weak or transient intermolecular interactions that become productive when a sufficient local concentration is reached. We therefore describe the 249–287 region as a sticker-like element that promotes condensation rather than as a stable oligomerization interface. Importantly, we now explicitly state that the molecular nature of these interactions remains unresolved and may involve homotypic helix–helix contacts, heterotypic interactions with other regions of Ent5, or both.

In the manuscript:

Results pages 6-7:

"Together, these results support a model in which residues 249–287 function as a sticker-like element within Ent5. Although neither the isolated Ent5 helix (249–287) peptide nor Ent5 Δ249–287 undergoes efficient phase separation individually, combining both components restores condensate formation. This activity in trans is consistent with a role for the helical region in increasing the effective interaction valency of Ent5 through transient intermolecular contacts that become productive when a sufficient local concentration is reached. However, the molecular identity of these contacts remains to be determined, and both heterotypic interactions with other regions of Ent5 and homotypic helix–helix interactions remain possible."

Discussion page 14:

"Our results further underscore the importance of discrete structural elements in driving phase separation. In Ent5, we identified a dynamic helix within the IDR that functions as a sticker. Although the isolated 249–287 peptide does not undergo phase separation on its own, it can restore condensation when added to Ent5 Δ249–287, indicating that the helix acts cooperatively with the surrounding disordered region and the ENTH domain. This behaviour is consistent with the helix contributing weak or

transient interactions that become productive at sufficient local concentrations, supporting its role as a sticker-like element rather than as an autonomous condensation module. The molecular nature of these interactions remains unresolved and may involve homotypic helix–helix contacts, heterotypic interactions with other regions of Ent5, or both. Membrane recruitment is likely to further enhance local protein concentration and facilitate condensate formation. The synergy between inducible secondary structure and flexible spacer regions appears to be a recurring principle in endocytic adaptors."

4. The presentation of Supplementary Fig. S2g could be improved.

We agree with the reviewer that the presentation of Supplementary Fig. S2g is visually not ideal. This arises because NaCl concentration was varied by dilution, which also resulted in small changes in the final protein concentration. We therefore plot the corrected final concentrations rather than nominal values, resulting in data points that do not form a uniformly aligned grid but accurately represent the concentrations used in the experiments. We have now clarified this point in the figure legend.

In (g), the NaCl concentration was varied by dilution. The plotted values represent the corrected final protein and NaCl concentrations after dilution; consequently, the protein concentrations vary slightly across the NaCl series and do not form a uniformly aligned grid.

5. The manuscript would benefit from one final round of proofreading. I noticed a few duplicated words, spacing problems, and other minor formatting issues.

We thank the reviewer for pointing this out. We have carefully proofread the manuscript and corrected duplicated words, spacing problems, and other minor typographical and formatting errors throughout. Corrections are marked in red.

Overall, the authors have addressed my main scientific concerns. The remaining points are minor and do not change my positive view of the study. I therefore support publication after minor revision.
